# Stochastic Emergence of Two Distinct Self-Replicators from a Dynamic Combinatorial Library

**DOI:** 10.1021/jacs.1c12591

**Published:** 2022-03-31

**Authors:** Gaël Schaeffer, Marcel J. Eleveld, Jim Ottelé, Peter C. Kroon, Pim W. J. M. Frederix, Shuo Yang, Sijbren Otto

**Affiliations:** † Centre for Systems Chemistry, Stratingh Institute, University of Groningen, Nijenborgh 4, 9747 AG Groningen, The Netherlands; ‡ Groningen Biomolecular Sciences and Biotechnology Institute & Zernike Institute for Advanced Materials, University of Groningen, Groningen 9747 AG, The Netherlands

## Abstract

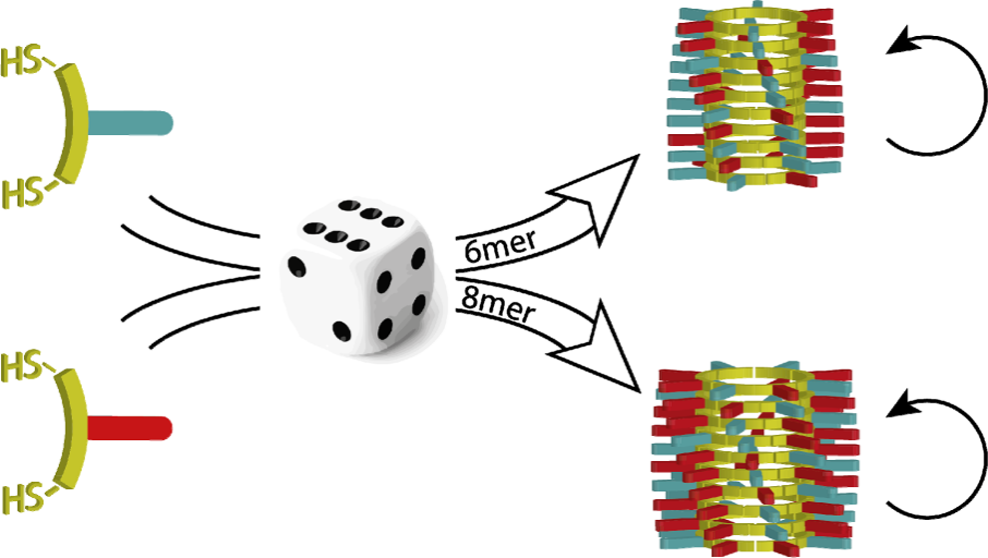

Unraveling how chemistry can give rise to biology is one of the greatest challenges of contemporary science. Achieving life-like properties in chemical systems is therefore a popular topic of research. Synthetic chemical systems are usually deterministic: the outcome is determined by the experimental conditions. In contrast, many phenomena that occur in nature are not deterministic but caused by random fluctuations (stochastic). Here, we report on how, from a mixture of two synthetic molecules, two different self-replicators emerge in a stochastic fashion. Under the same experimental conditions, the two self-replicators are formed in various ratios over several repeats of the experiment. We show that this variation is caused by a stochastic nucleation process and that this stochasticity is more pronounced close to a phase boundary. While stochastic nucleation processes are common in crystal growth and chiral symmetry breaking, it is unprecedented for systems of synthetic self-replicators.

## Introduction

Stochasticity plays an important role in numerous processes in biology. Random fluctuations in environmental conditions (environmental stochasticity) greatly influence evolutionary processes on the scale of populations.^1^ On the cell level, fluctuations in transcription and translation processes can cause genetically identical cells to have different protein expressions and growth rates, which is thought to be one of the major drivers of phenotypic heterogeneity.^2,3^

On the molecular scale, stochastic processes are also found to play a prominent role in the nucleation of crystallizations.^4,5^ Coupled to an autocatalytic propagation step, this can even enable chiral symmetry breaking.^6^ Complete chiral purity can be obtained from a mixture containing both enantiomers of an (organic) molecule when a stochastic nucleation event is coupled to autocatalytic secondary nucleation, a recycling mechanism, and a racemization process of the single molecule.^7−9^ This combination of stochastic emergence and autocatalysis is thought to be a possible scenario for the origin of homochirality in nature.^10−12^

A stochastic nucleation step is also found in supramolecular polymerizations that often follow a nucleation–elongation mechanism.^13^ The characteristic lag phase in the formation of these polymers is a result of this stochasticity.^14^ The final structure of these assemblies is, however, deterministic: the nature of the building blocks that constitute the polymer determines what assembly is formed.

The same can be said for self-replicating molecules. Self-replicating molecules have the ability to autonomously catalyze their copying, where information of the system components is transferred to the next generation.^15,16^ Most self-replicators operate by a duplex formation mechanism. Many examples of this have been reported based on DNA,^17^ RNA,^18,19^ peptides,^20,21^ as well as completely synthetic molecules.^22,23^ Based on the nature of the replication mechanism and the availability of a single type of building block, there is usually only a single outcome possible: making more exact copies of the template molecule. There are also self-replicating systems that are driven by supramolecular polymerization.^24−26^

We have previously reported pseudopeptide^27^ building blocks that are composed of an aromatic dithiol core connected to a pentapeptide. When a dynamic combinatorial library (DCL) is prepared from one of these building blocks that is left to oxidize by atmospheric oxygen in an aqueous borate buffer, initially, an interconverting mixture of macrocycles with various ring sizes is formed. This mixture contains predominantly three- and four-membered macrocycles. When the DCL is not agitated, the final composition remains dominated by these small macrocycles. However, upon mechanical agitation through stirring, a larger, self-replicating macrocycle can emerge that, during its replication process, consumes most of the smaller macrocycles. This larger macrocycle becomes the main species when the DCL is fully oxidized to disulfides. The formation of the larger macrocycle is autocatalytic and driven by its assembly into fibers held together by a combination of hydrophobic and β-sheet interactions. These fibers, once nucleated, elongate by consuming smaller macrocycles from the solution.^28,29^ By physical agitation, self-replication is facilitated through fiber breakage, increasing the number of growing fiber ends. In these systems, it is possible to obtain different replicators (with various macrocycle sizes) by changing the peptide sequence^30^ or the experimental conditions, for example, the mode of agitation or the solvent composition.^26,31^

However, all aforementioned systems are still deterministic: the outcome is controlled by the structure of the molecules in the system and the reaction conditions. Here, we report a supramolecular self-replicating system, where the nature of the replicator that emerges is not deterministic but determined stochastically. We also show that stochasticity is most pronounced closest to a phase boundary.

## Results and Discussion

**Figure 1:**
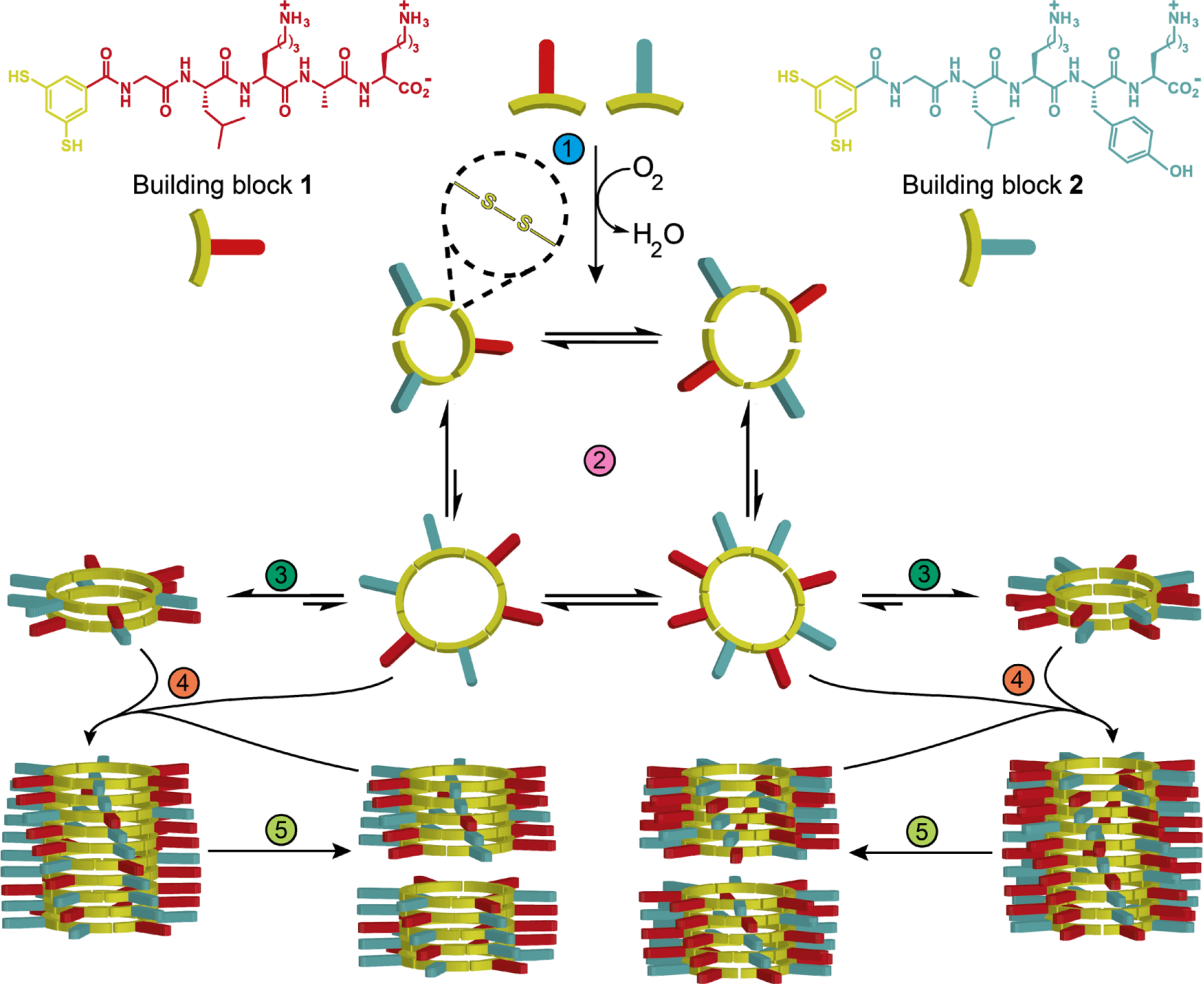
Molecular structures of building blocks **1** and **2** and schematic representation of the self-replication mechanism. Dithiol-building blocks, **1** and **2**, are oxidized (1) to form a mixture of macrocycles with various ring sizes (2) that interconvert using thiol–disulfide chemistry. Two different nucleation steps can occur (3), leading to the formation of stacks of macrocycles containing six or eight monomer units. Both nuclei can elongate (4) to form fibers by consuming smaller macrocycles from the solution. Fragmentation of the fibers by mechanical agitation when the stack is sufficiently long (5) leads to an elongation/fragmentation regime, enabling exponential growth.

Mixing structurally similar replicators in a DCL can lead to often unexpected emergent properties such as spontaneous diversification of replicators^32^ or parasitism.^33^ This work focuses on mixtures of building blocks **1** and **2** (see Figure 1), which are composed of an aromatic dithiol core connected to pentapeptides that differ from each other in the fourth amino acid in the sequence: alanine in **1** and tyrosine in **2**. When a DCL is prepared containing only **1** (3.8, 50 mM borate buffer, pH 8.2), a self-replicating eight-membered macrocycle (octamer **1**_**8**_) emerges.^30^ Similarly, in a DCL containing only **2**, a self-replicating three-membered macrocycle (trimer **2_3_**) emerges.^34^ From previous work, we know that with increasing hydrophobicity in the peptide side chain, the ring size of the self-replicating macrocycle becomes smaller.^30^ The same effect is observed here as the more hydrophobic building block containing a Tyr-residue (**2**) assembles into a three-membered macrocycle, where the less hydrophobic building block containing an Ala-residue (**1**) assembles into an eight-membered macrocycle.

Because **1** and **2** form self-replicating macrocycles of different ring sizes (octamer **1**_**8**_ and trimer **2**_**3**_) in a stirred DCL, we wanted to investigate the behavior of these building blocks when combined in a single system.

**Figure 2:**
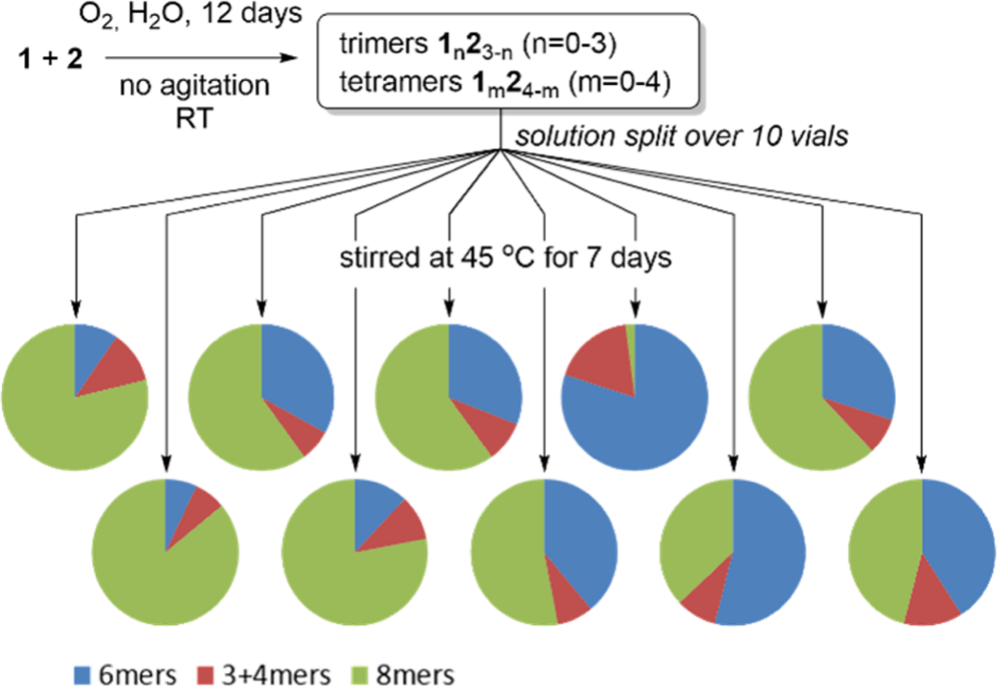
Final compositions of a DCL made from equimolar amounts of **1** and **2**, split into 10 smaller aliquots. Different outcomes with different ratios between hexamer, octamer, and trimer + tetramer were found.

A DCL was made from equimolar amounts of **1** and **2** (total concentration 1.0 mM) in aqueous borate buffer (50 mM, pH 8.12) and left unstirred at room temperature until 85% of the thiols were oxidized to disulfides by atmospheric oxygen, forming mostly trimer and tetramer macrocycles (see Figure S5). This mother solution was deliberately not agitated and kept at room temperature, as under such conditions replicator emergence is sluggish. At this point, the DCL was split into 10 samples of equal volume and composition that were stirred at 1200 rpm at 45 °C to speed up the replication process. After 7 days, essentially all of the thiols were oxidized to disulfides, and the system was no longer able to exchange efficiently. The composition of the DCLs was determined based on the relative peak areas obtained from reverse-phase ultra-performance liquid chromatography (RP-UPLC) analysis (see Figures S1–S14). Even though RP-UPLC is an indirect measurement of fiber formation, the data correlates well with the direct measurement of β-sheet formation using ThT fluorescence (Figure S56). All DCLs contained a residual amount of trimer and tetramer macrocycles, as well as both the mixed hexamer and octamer macrocycles. We observed a large variety in the ratio between these differently sized macrocycles (see Figure 2). Some DCLs would be dominated by octamer macrocycles, some by hexamer macrocycles, and others contained similar amounts of both the hexamer and octamer macrocycles.

**Figure 3:**
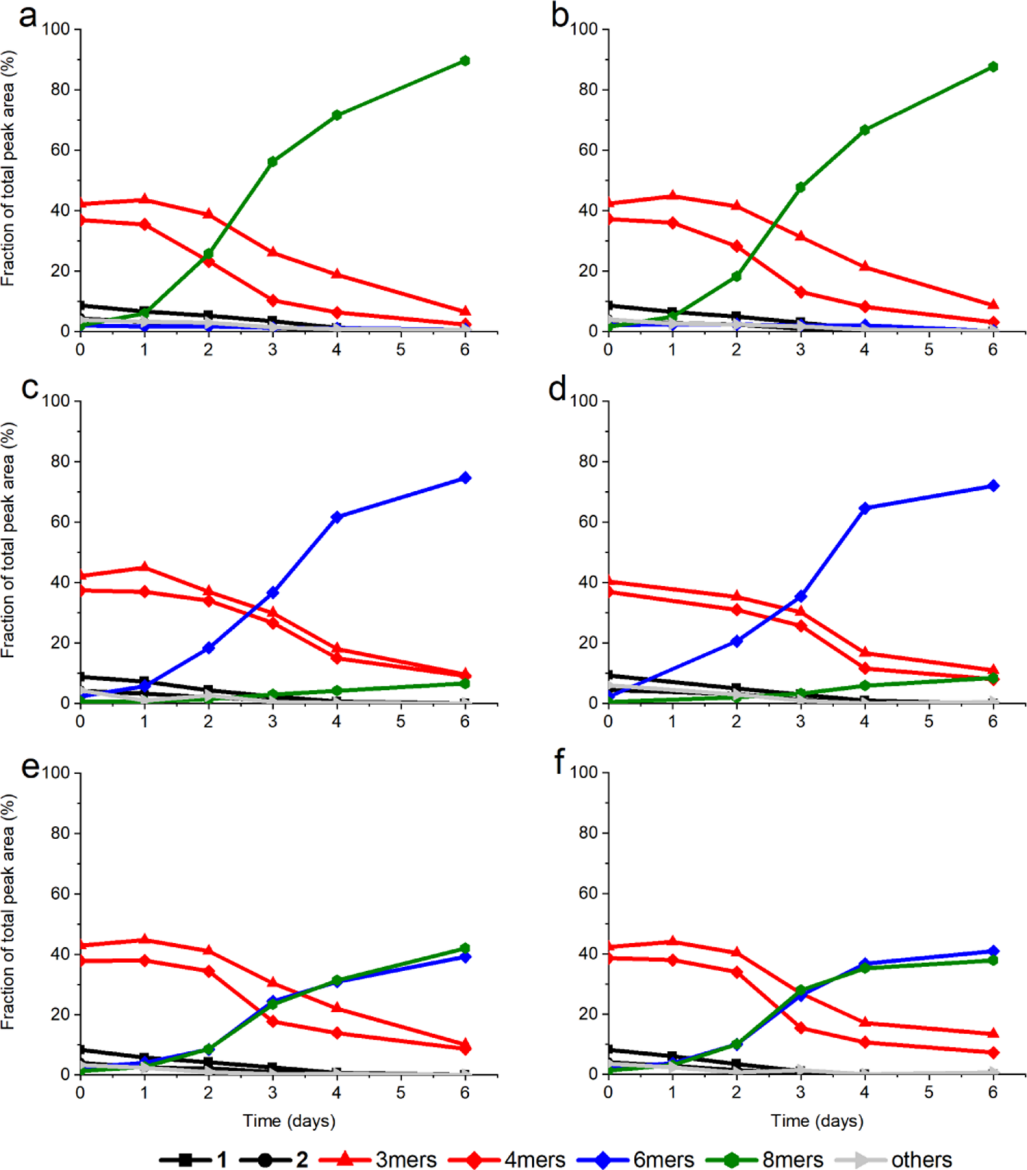
Kinetic traces of DCLs seeded with preformed self-replicators. (a, b) Seeded with 10 mol % preformed mixed octamer, (c, d) seeded with 10 mol % preformed mixed hexamer, and (e, f) seeded with 5 mol % each of preformed mixed hexamer as well as 5 mol % preformed mixed octamer.

Both the hexamer- and octamer-mixed macrocycles were found to self-assemble into supramolecular fibrous structures (see Figure S55) and exhibit self-replication upon agitation at elevated temperatures (see Figure 3). Circular dichroism (CD) spectra of samples dominated by hexamers or octamers show signatures similar to previously reported peptide replicators that replicate using β-sheet formation (see Figure S54). A thioflavin T (ThT) fluorescence assay confirmed that both the hexamer and octamer replicators form β-sheets (see Figure S53). In contrast to the previously reported case,^32^ these replicators do not seem to show a strong preference for incorporation of either of the building blocks and therefore incorporate both in similar amounts.

Several repeats of this experiment (see Figures S46–S48) resulted in widely varying amounts of hexamer and octamer replicators. Histograms with the amounts of trimers + tetramers, hexamers, and octamers obtained in these experiments are shown in Figure S49. Stochasticity showed no discernable dependence on overall building block concentration in the range tested (50 μM to 2.0 mM; Figure S51). We envisaged that the variation in product distribution might be caused by a stochastic nature of the nucleation process. To confirm this hypothesis, experiments were performed where the nucleation step was bypassed by the addition of preformed replicators. Again, a single DCL was prepared by mixing equimolar amounts of **1** and **2** and left unstirred at room temperature until 85% of the thiols were converted to disulfides, forming trimer and tetramer macrocycles. This time the DCL was split into six smaller DCLs of which two were seeded with 10 mol % preformed hexamer replicators, two with 10 mol % preformed octamer replicators, and two with 5 mol % each of both hexamer and octamer replicators (see Figure 3a–f).

These seeded DCLs were stirred at room temperature for 6 days and monitored over time with ultra-performance liquid chromatography-mass spectrometry (UPLC-MS). In the DCLs that were seeded with preformed hexamers, replicators would consume most of the trimer and tetramer macrocycles to form 70–75% of mixed hexamer replicators and only up to 10% of octamer replicators. This shows that the mixed hexamer macrocycles are self-replicators with (very little or) no cross-catalysis to the octamer macrocycles. Similarly, in the DCLs that were seeded with preformed octamer replicators, most of the trimer and tetramer macrocycles were converted into octamer replicators, reaching 85–90% of the final library composition. In these DCLs, only small amounts of hexamer replicators could be detected, indicating that also the octamer macrocycles are replicators with (very little or) no cross-catalysis toward the hexamer macrocycles. We attribute the dominance of autocatalysis over cross-catalysis to the relative inefficiency of templating a six-membered ring by a stack of eight-membered rings and vice versa. Interestingly, in the DCLs that were seeded with both types of preformed replicators, both the hexamer and octamer replicators would replicate to reach ∼40% each in the final library composition at the expense of the trimer and tetramer macrocycles. This data confirms that the two sets of replicators have comparable growth kinetics, which allows them to coexist in a single DCL when the nucleation of both the self-replicating macrocycles, which is mimicked here by adding preformed replicators, occurs at the same time. When only the nucleation of one of the replicators is artificially facilitated (by adding only preformed replicators of one macrocycle size), that replicator will grow to dominate the final composition of the DCL. The other (nonseeded) replicator still has the possibility to nucleate spontaneously, but by the time that happens the seeded replicator is already present in such a large amount that the newly formed replicator is not able to efficiently compete for the building blocks that they both require for growth. In principle, an octamer replicator could also grow at the expense of the hexamer replicator and vice versa. However, the interconversion between two replicator assemblies tends to be slower than the growth of replicators from small-ring precursors.^35^

We envisage that the differences in final library composition observed in the experiment, where all nucleation events occur spontaneously (Figure 2), are due to different nucleation times for the hexamer and octamer replicators. In some DCLs, the hexamer replicators nucleate first, resulting in a final library composition dominated by hexamer replicators. In other DCLs, the octamer replicator nucleates first, resulting in the octamer replicator dominating the final library composition. There are also cases, where the nucleation events for both replicators closely follow each other, allowing both sets of replicators to grow simultaneously and coexist in the final DCL. Random fluctuations in the reaction mixture can lead to the spontaneous formation of nuclei for either of the self-replicating macrocycles. The nucleus that is formed first will give the corresponding self-replicator a head start. We therefore believe that the nucleation events are of a stochastic nature. However, we cannot strictly rule out the influence of variables that cannot readily be controlled in parallel experiments, which include small variations in the shape and movement of the stirring bars and resulting small differences in fluid dynamics and variations in the surface microstructure of the vials and stirring bars.

To obtain an estimate of nucleation times for the different replicators, the RP-UPLC traces were fit to a simplified model. Stochastic nucleation times for hexamer (*t*_0,hex_) and octamer (*t*_0,oct_) replicators were fitted based on the experimental data, and subsequent replicator growth was described using ordinary differential equations (ODEs, Scheme S1). Molecules were defined to be either hexamers, octamers, or precursors (*i.e.*, monomers, trimers, and tetramers). All concentrations were normalized to be between 0 and 100. The nucleation process was simulated using a sigmoidal function (*f*), which steeply switched from 0 (before nucleation) to 1 (after nucleation). For every experiment, a system of three coupled ODEs was fit. This system has four free parameters: *t*_0,hex_, *t*_0,oct_, *k*_hex_, and *k*_oct_. Since the self-replication rate constants *k*_hex_ and *k*_oct_ should be identical for all systems, they were shared between the ODE systems. This results in 2 × *n* + 2 free parameters for *n* experiments. This revealed a high covariance between parameters *t*_0,hex_ and *t*_0,oct_. Because of this, the nucleation times were redefined as stated in Scheme S1. Time *t* = 0 was defined as the moment the agitation of the DCL was started (which is also when the first RP-UPLC measurement was taken). Since the mixtures were prepared well before that, integration of the ODEs was started at *t* = −2.

**Figure 4:**
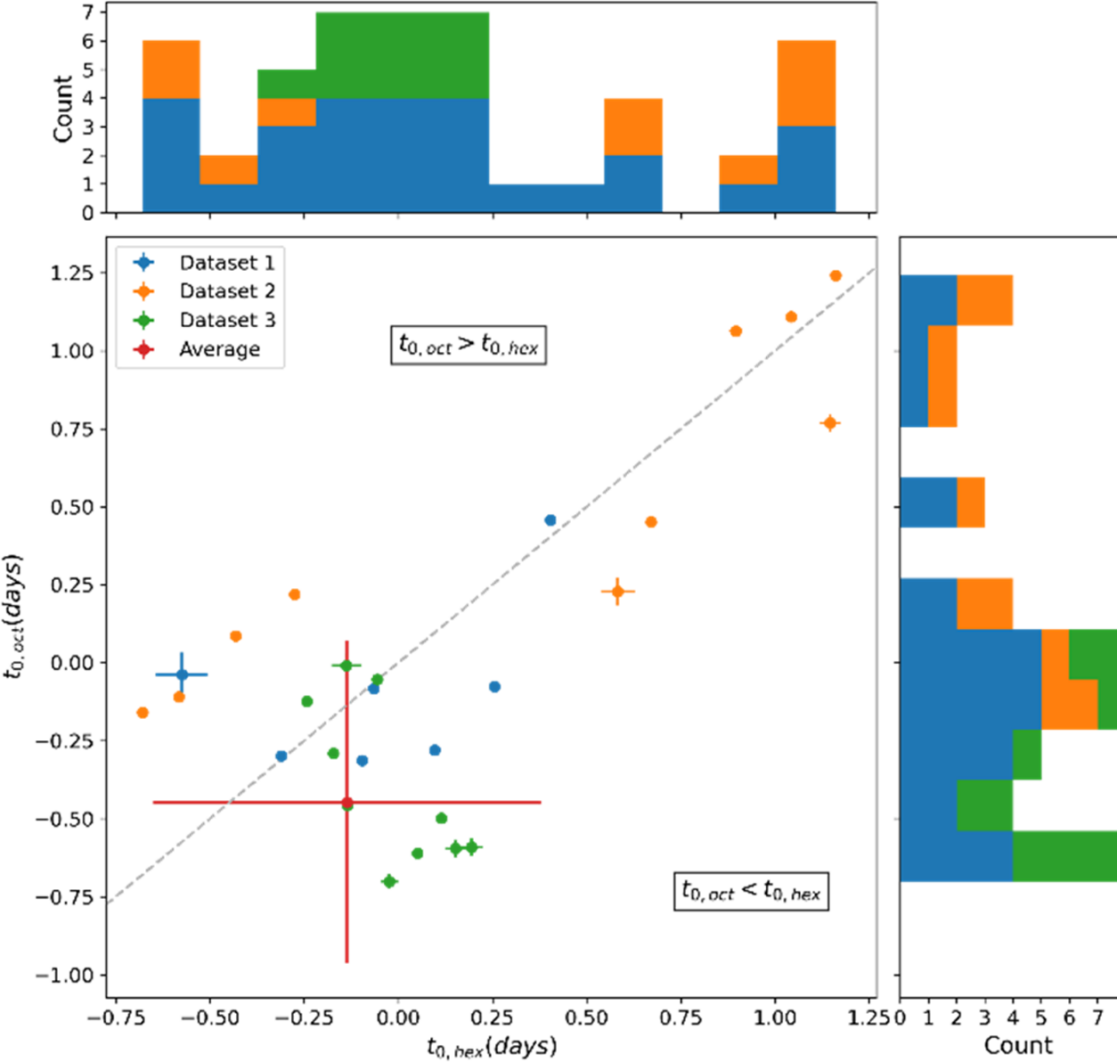
Resulting fit parameters for three repeats, with 10 aliquots each, of the emergence experiments described in Figure 2. *X* axis depicts nucleation time of the hexamer replicators, *Y* axis of the octamer replicators. If a data point is found above the diagonal (dashed line) hexamers nucleated before octamers, and vice versa, showing the spread in nucleation times. Error bars indicate standard deviations in the fitting parameters, but these are too small to be observed for most points (*R*^2^ = 0.933). The weighted average nucleation time is indicated in red, showing that on average, octamers nucleate before hexamers. This observation is consistent with the fact that octamers are the dominant species in the majority of the samples. Above the axes, histograms of the found nucleation times are plotted.

The resulting fit is plotted in Figure S57. The corresponding best fit parameters for the nucleation times of the hexamer and octamer macrocycles in the various experiments are shown in Figure 4. The fact that the observed data can be fitted using a model featuring stochastic nucleation lends support to the notion that the different ratios in which the replicators are formed results from stochastic variations in the time interval between the nucleation events for each replicator. Some of the experiments showed behavior not described by the model, where the growth of both hexamer and octamer replicators stopped while there was still sufficient precursor left (presumably, all monomer was oxidized, stalling the replication). In these cases, data points that do not correspond to an exponential growth regime were not included in the fitting process.

**Figure 5:**
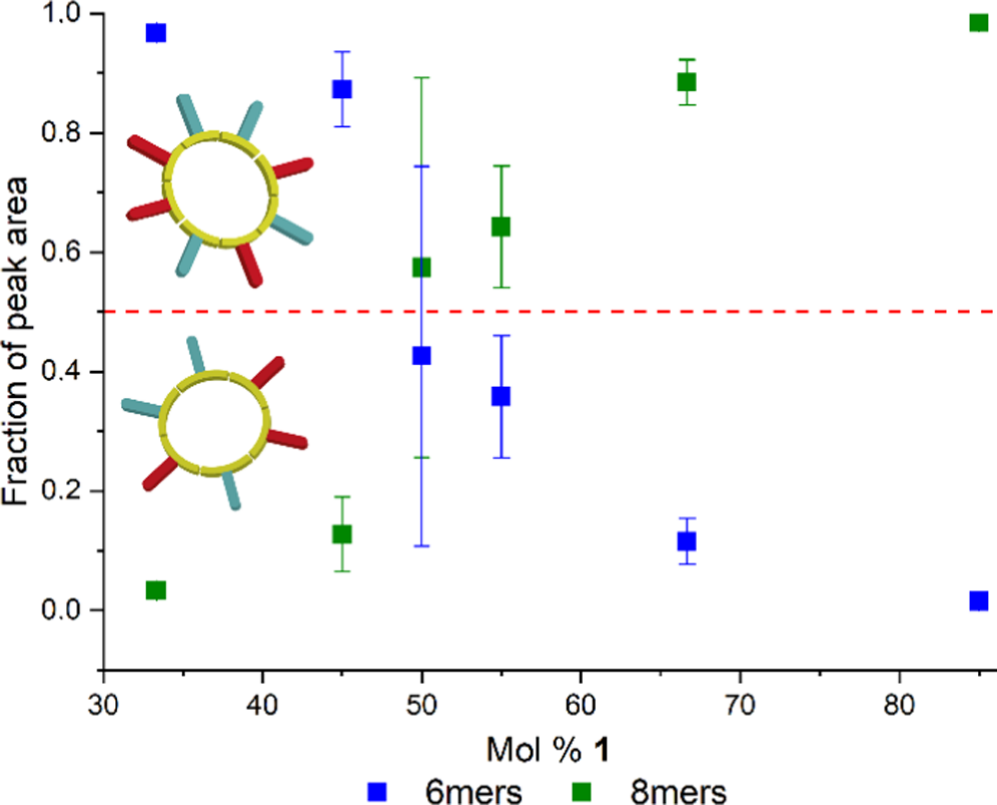
Variation in the fraction of the hexamer (blue) and octamer (green) replicators in the final library composition (determined by RP-UPLC) as a function of the amount of **1**. The fraction is defined as the amount of observed replicators divided by the total amount of replicators (hexamers + octamers). The data points show the average and the error bars the standard deviation. The data points at 33, 45, 55, and 67% **1** are averaged over two repeats with five samples each and the data point at 85% **1** is averaged over five samples. The data point at 50% **1** is averaged over five repeats with a total of 45 samples. The dotted red line indicates the boundary between DCLs that are rich in hexamers (below the line) and rich in octamers (above the line).

In our experience, it is rare that there is stochasticity at play in mixtures of dithiol-building blocks that form replicators. It is logical to assume that such behavior requires comparable nucleation probabilities of both replicators that are formed. To probe this hypothesis, additional experiments were performed, where the ratio between **1** and **2** was varied, which could potentially change the nucleation probabilities of the replicators. We expected that **1**-rich samples would be biased toward octamer nucleation, while **2**-rich samples would show the preferential nucleation of hexamers, in line with the trend in ring sizes of the replicators formed from these building blocks in isolation.^30,34^ These experiments were performed in a similar fashion as the initial experiments (see Figure 2). However, the relative amount of **1** in the initial DCLs was varied from 50% to 15, 33, 45, 55, 67, and 85%. The total concentration of **1 + 2** was kept constant at 1.0 mM. These DCLs were oxidized by ambient oxygen without agitation until a disulfide content of approximately 85% was reached, after which each DCL was split into five aliquots that were agitated at 45 ^o^C for 7 days. After all monomers had been consumed, the final library composition was analyzed by RP-UPLC. The fraction of octamer and hexamer replicators as a function of the total amount of replicators (hexamers + octamers) was determined for every library as well as the variation (standard deviation) of this fraction between the different DCLs with the same building block ratio (see Figure 5 and Tables S5–S12). This variation is a measure of stochasticity.

In the libraries with a large bias toward **1** (85 and 67%) predominantly, octamer replicators are formed with a small standard deviation. When the bias is smaller (55% **1**), the octamer replicators are still formed preferentially, but there is significantly more hexamer replicator produced. The ratio between hexamer and octamer replicators also varies more compared to the libraries with a larger bias, as indicated by the larger standard deviation. A similar, but opposite, effect is observed when the libraries are biased toward **2**. When the bias is strong (33% **1**), the hexamer replicators are formed almost exclusively, and when the bias is smaller (45% **1**), the hexamer replicators become less dominant. Also, in this case, the standard deviation decreased with increasing building block bias. When the building block ratio has an even larger bias toward **2** (15% **1**), the system loses its preference for hexamer and/or octamer macrocycles and, instead, produces a larger range of different sized macrocycles (Figure S7 and Table S5).

The largest standard deviation was observed for the libraries containing 50% **1**. In these libraries, the average fraction of octamer is approximately 0.5, which indicates that hexamer and octamer replicators have a similar chance of emerging under these conditions. These results show that the stochastic emergence behavior that is observed in this system finds its origin in the fact that at 50% **1**, the system resides close to the boundary between two phases: the **1**-rich phase in which hexamer replicators are formed preferentially and the **2**-rich phase in which octamer replicators are preferred.

## Conclusions

We have found a two-building block system from which two distinct self-replicators can emerge. Unlike previous reports, where different outcomes could only be achieved by changing the experimental conditions,^26,31,36^ here, different self-replicators emerge in a stochastic fashion. Starting from equimolar amounts of **1** and **2**, both self-replicators incorporate the two building blocks (**1** and **2**) in similar amounts, have a similar chance of nucleating, comparable growth kinetics, and very little (or no) cross-catalysis toward each other.

The nucleation event that takes place first dictates which self-replicator will be dominant in the system. Depending on the time taken by the competing replicator to nucleate, that self-replicator will also be present to a greater or lesser extent. When the time interval between the nucleation events is short, both replicators will be present in similar amounts, and with increasing time intervals between nucleation events, the final fraction of the self-replicator that nucleated first increases.

Stochasticity was most pronounced when the system was at the boundary between two different phases: one in which hexamer self-replicators are the preferred species and another where octamer self-replicators are favored. At this boundary, the chance of nucleation is similar for each self-replicator.

We believe that the minimal criteria to observe stochasticity in the nucleation process are the absence of cross-catalysis between the different replicators, similar probabilities of nucleation for both replicators, and similar growth rates after nucleation. Here, we reported one example of such a system, but we envisage that this could be generalized to other examples as long as they meet these criteria.

Stochastic events are known to play an important role in chiral symmetry breaking in crystallization processes as well as various processes in biology but lack precedent in systems of synthetic self-replicators. While these results show stochasticity during the process of replicator emergence, the challenge is now to also obtain similar stochastic behavior in replicator mutation.

## Supporting Information Available

Methods of DCL and sample preparation; RP-UPLC and LC-MS methods; RP-UPLC chromatograms; mass spectra; repeats of experiments; ThT fluorescence assay; CD spectra; transmission electron microscopy (TEM) micrographs; parameters for data fitting, and model system of ODEs (PDF)

## Abbreviation

DCL: dynamic combinatorial library
rpm: revolutions per minute
RP-UPLC: reverse-phase ultra-performance liquid chromatography
ODE: ordinary differential equation
*t*_0,hex_: nucleation time hexamer replicator
*t*_0,oct_: nucleation time octamer replicator
ThT: thioflavin T
TEM: transmission electron microscopy
CD: circular dichroism
