# Supplementary material for: Stochastic Emergence of Two Distinct Self-Replicators from a Dynamic Combinatorial Library
Source: J Am Chem Soc. 2022 Mar 31;144(14):6291–7. doi: 10.1021/jacs.1c12591 (PMC9011346; doi:10.1021/jacs.1c12591)
Supplement: Supplementary file 1 — ja1c12591_si_001.pdf [file ja1c12591_si_001.pdf]

Supplementary Information for

Stochastic emergence of two distinct self-replicators from a dynamic combinatorial library

Gaël Schaeffer,[‡°] Marcel J. Eleveld,[‡°] Jim Ottelé,[°] Peter C. Kroon,[°] Pim W.J.M. Frederix,[°] Shuo Yang,[°] and Sijbren Otto*[°]

[°] Centre for Systems Chemistry, Stratingh Institute, University of Groningen, Nijenborgh 4, 9747 AG Groningen, The Netherlands

Contents

1. Preparation of dynamic combinatorial libraries 3
2. UPLC Method 4
3. Analysis and characterization of dynamic combinatorial libraries 5
4. Quantitative RP-UPLC analysis of dynamic combinatorial libraries 8
5. Mass spectrometry 11
6. Repeats of emergence experiments 28
7. Data for DCLs with varying building block ratios 32
8. Changing experimental conditions 34
9. Thioflavin T (ThT) fluorescence assay 38
10. Circular dichroism (CD) spectroscopy 39
11. Negative Staining Transmission Electron Microscopy 40
12. Correlation between UPLC and ThT data 41
13. Data Fitting 42
14. References 44

1. Preparation of dynamic combinatorial libraries

Building blocks **1** and **2** were purchased from Cambridge Peptide (Birmingham). All the libraries were prepared by dissolving the building blocks (1.0 mM) in borate buffer (25 mM B_2O_3 in water, pH 8.2), and stirring the solution in the presence of oxygen from the air. The pH of the solution was adjusted by the addition of 1 M KOH solution such that the final pH was 8.2.

All library experiments were performed at ambient temperature. A small aliquot of each sample was moved to another vial and diluted five or ten times with doubly distilled water prior to HPLC or UPLC(-MS) analysis.

The buffer was prepared from boric acid (Merck Chemicals) dissolved in doubly distilled water from in-house double distillation facilities. Sodium perborate used for the oxidation of the thiols was purchased from Sigma Aldrich. Acetonitrile (ULC-MS grade), water (ULC-MS grade) and trifluoroacetic acid (ULC-MS grade) were obtained from Biosolve BV. Libraries were prepared in clear HPLC glass vials (12 × 32 mm) closed with Teflon-lined snap caps purchased from Jaytee. Library solutions were stirred using Teflon coated micro-stirrer bars (2 × 2 × 5 mm) obtained from VWR. Samples were stirred on an Heidolph MR Hi-Mix D magnetic stirrer at 1200 rpm.

In the experiments with 10 parallel repeats, a library of 6 mL ([**1**] = [**2**] = 0.5 mM) was set up and allowed to oxidize at room temperature by the oxygen from the atmosphere until 70% of the monomers were oxidized (no agitation – ca. 12 days). This library was then split into 10 fractions (of 500 µL each). These 10 libraries were stirred at 45 ^{o}C and their compositions were monitored by RP-UPLC.

2. UPLC Method

UPLC analyses were performed on Waters Acquity UPLC I-class, H-class or H+class systems equipped with a PDA detector. All analyses were performed using a reversed-phase UPLC column (Aeris Peptide 1.7 μm XB-C18 150 × 2.10 mm, purchased from Phenomenex). UV absorbance was monitored at 254 nm. Column temperature was kept at 35 °C. UPLC-MS was performed using a Waters Acquity UPLC H-class system coupled to a Waters Xevo- G2 TOF. The mass spectrometer was operated in the positive electrospray ionization mode. Capillary, sampling cone, and extraction cone voltages were kept at 2.5 kV, 30 V, and 4 V, respectively. Source and desolvation temperatures were set at 150°C.

Solutions containing peptides **1** and/or **2** and their oxidation products were prepared by diluting a small aliquot of a DCL in water. These diluted samples were analyzed using the following methods (all gradients are linear):

Method A

5 μL of DCL in 45 μL water

Solvent A: double distilled water (0.05 V/V% trifluoroacetic acid), Solvent B: 80% MeOH + 20% iPrOh (0.05 V/V% trifluoroacetic acid)

time (min)	flowrate (mL/min)	% A	% B
0.0	0.2	60	40
11.0	0.2	30	70
11.5	0.2	5	95
12.0	0.2	5	95
12.5	0.2	90	10
17.0	0.2	90	10

Method B

10 μL of DCL in 40 μL water

Solvent A: ULC-MS grade water (0.1 V/V% trifluoroactic acid), Solvent B: ULC-MS grade acetonitrile (0.1% V/V% trifluoroactic acid)

time (min)	flowrate (mL/min)	% A	% B
0.0	0.3	90	10
1.0	0.3	90	10
1.3	0.3	75	25
3.0	0.3	73	27
11.0	0.3	70	30
11.5	0.3	5	95
12.0	0.3	5	95
12.5	0.3	90	10
17.0	0.3	90	10

3. Analysis and characterization of dynamic combinatorial libraries

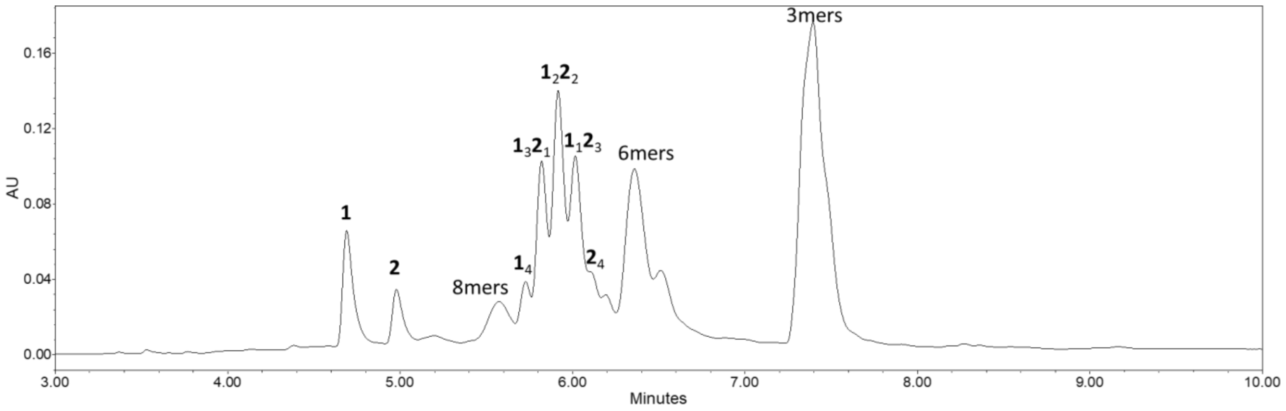

Figure S1. RP-UPLC trace (monitored at 254 nm using method A) of the product mixture obtained by oxidation of peptides **1** and **2** for 11 days after which it was stirred at 1200 rpm for 3 days. In this sample, one can observe the presence of both replicators (hexamers and octamers).

No replication when quiescent:

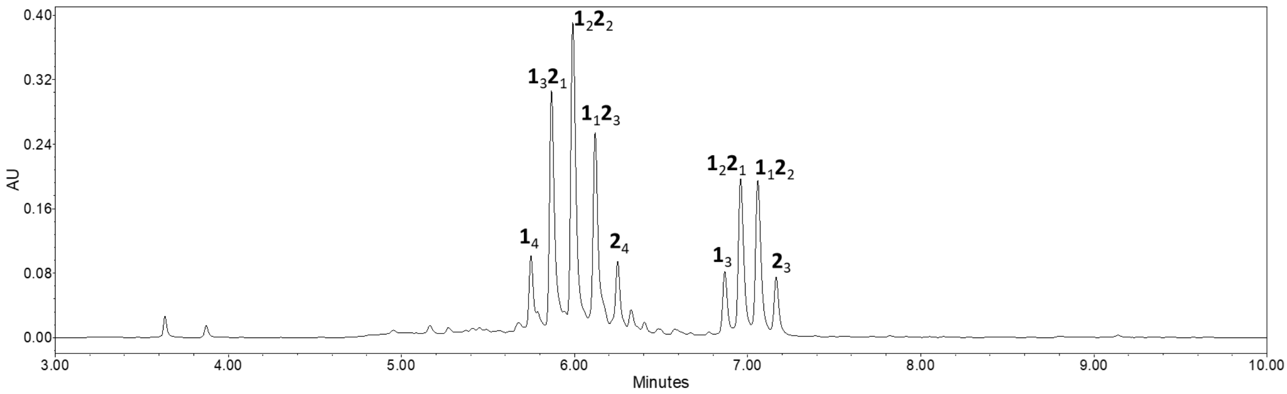

Figure S2. RP-UPLC trace (monitored at 254 nm using method A) of the product mixture obtained by oxidation of peptides **1** and **2** without agitation for 5 weeks. In this sample trimers and tetramers dominate.

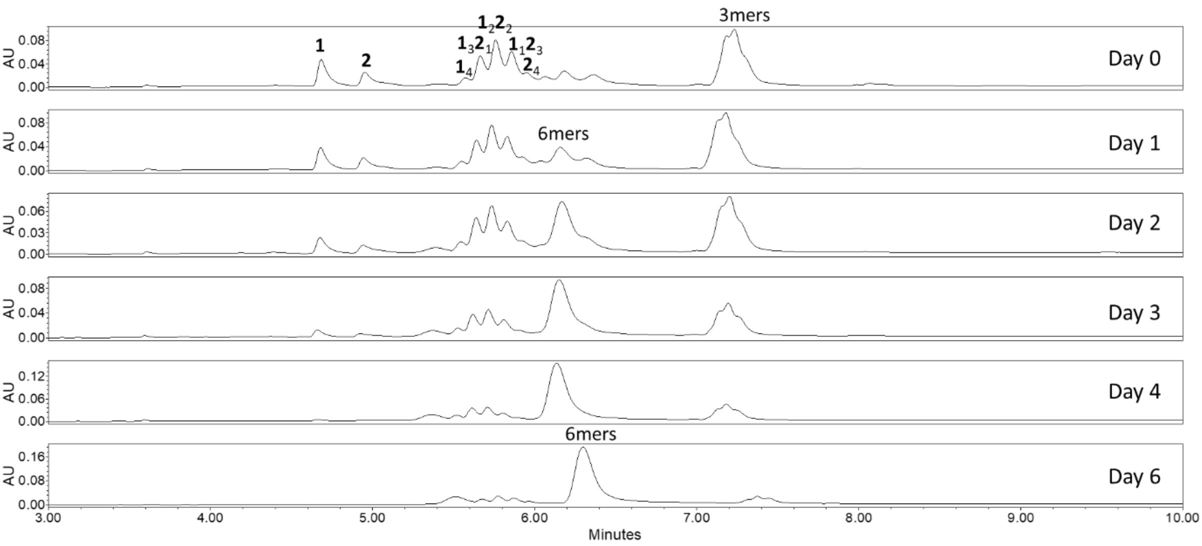

Figure S3. Time evolution: RP-UPLC traces (monitored at 254 nm using method A) of the product mixture obtained by oxidation of peptides **1** and **2** for 11 days after which it was stirred at 1200 rpm for 6 days. In this DCL, one can observe the emergence and growth of the 6mer replicators. Small shifts in retention times are observed.

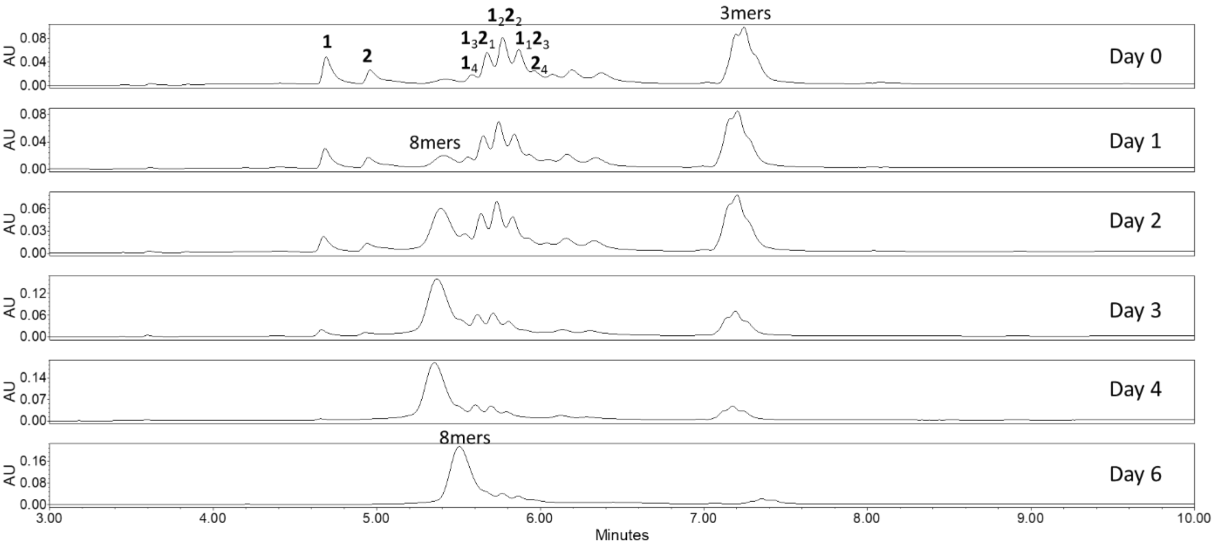

Figure S4. Time evolution: RP-UPLC traces (monitored at 254 nm using method A) of the product mixture obtained by oxidation of peptides **1** and **2** for 11 days after stirring at 1200 rpm for 6 days. In this sample, one can observe the emergence and growth of the 8mer replicators. Small shifts in retention times are observed.

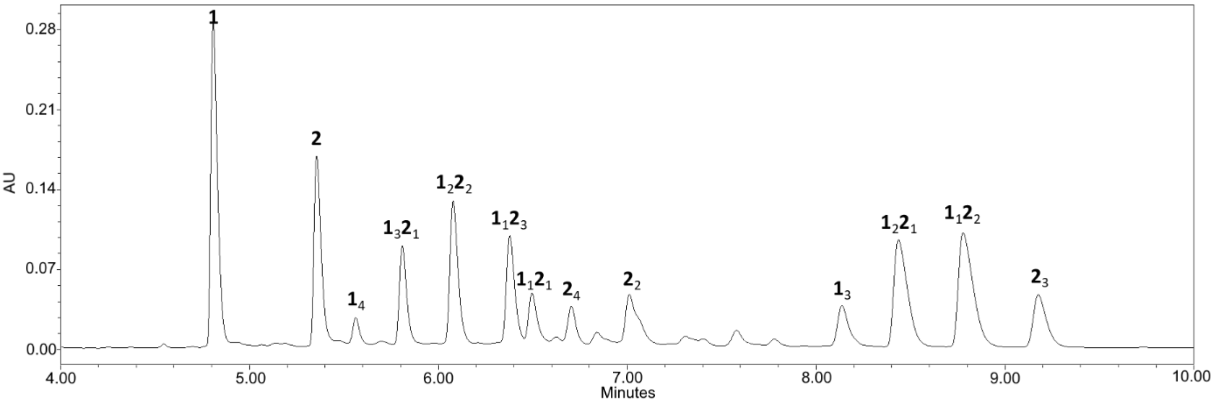

Figure S5. RP-UPLC trace (monitored at 254 nm using method B) of the product mixture dominated by trimers and tetramers obtained by oxidation of peptides **1** and **2** without agitation for 7 days.

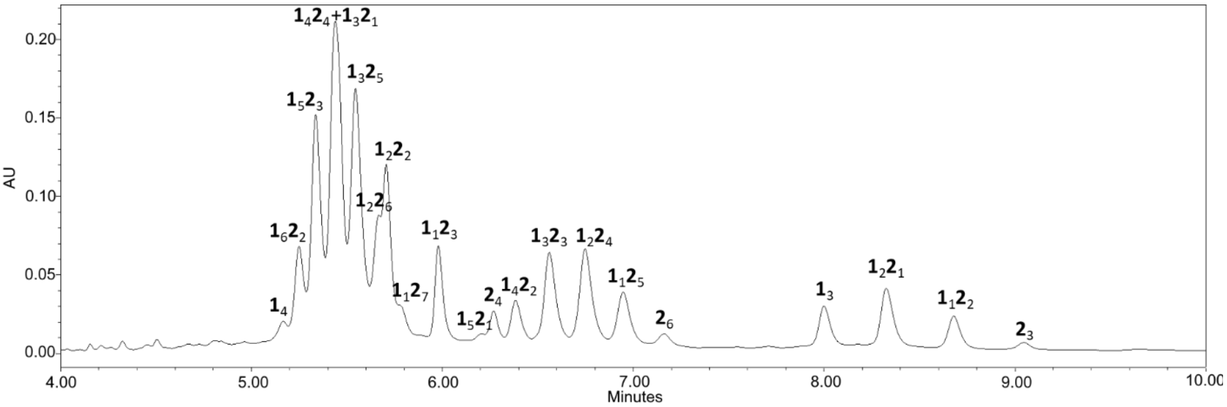

Figure S6. RP-UPLC trace (monitored at 254 nm using method B) of the product mixture obtained by oxidation of peptides **1** and **2** for 7 days after which it was stirred at 1200 rpm for 7 days. In this sample, one can observe the presence of both replicators (hexamers and octamers). Using method B the 6mers and 8mers with different compositions can be separated from each other.

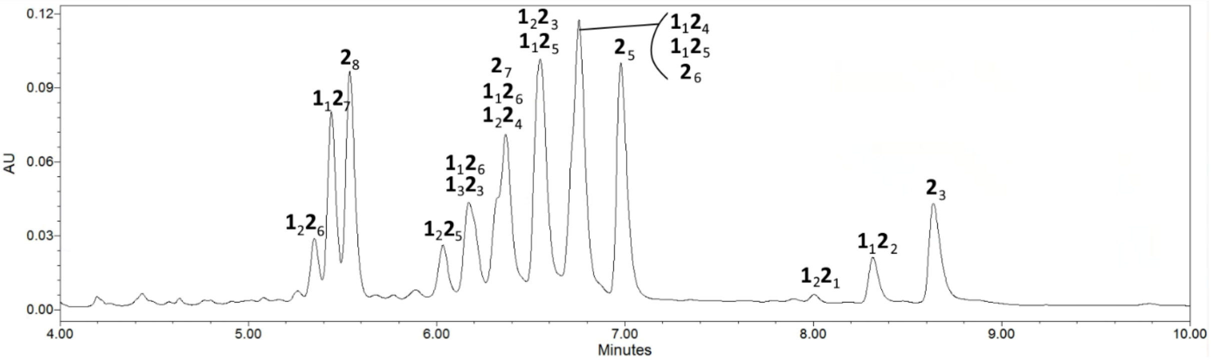

Figure S7. RP-UPLC trace (monitored at 254 nm using method B) of the product mixture obtained by oxidation of peptides **1** (15 mol%) and **2** (85 mol%) for 7 days after which it was stirred at 1200 rpm for 7 days. In this sample, one can observe the presence of many different macrocycle sizes (trimers, pentamers, hexamers, heptamers and octamers).

4. Quantitative RP-UPLC analysis of dynamic combinatorial libraries

Quantitative analysis of the library composition was done based on the total peak area that was obtained from RP-UPLC analysis monitored at a wavelength of 254 nm. This method is only valid if the molar absorption of **1** and **2** at 254 nm is the same and independent of the environment (i.e. type of ring) in which these building blocks reside. **Figures S8-S12** show that the total peak area that is obtained at 254 nm is constant throughout the experiment. This is shown for a DCL that is dominated by hexamers as well as one that is dominated by octamers at the end of the experiment. The total peak area also remains constant when the ratio between **1** and **2** is varied (**Figures S13+S14**).

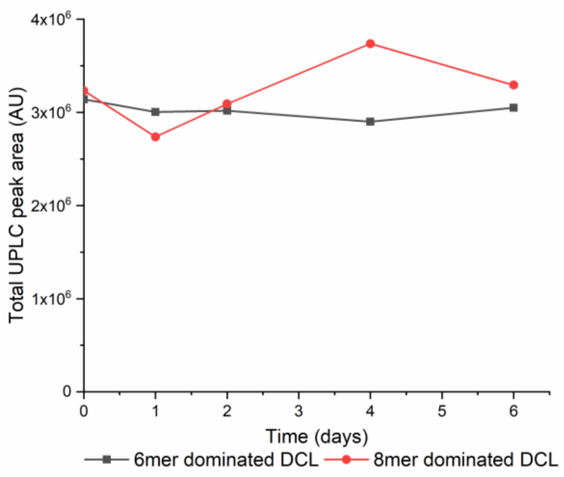

Figure S8. Time evolution of the total peak area obtained from RP-UPLC analysis (monitored at 254 nm using method A) of two product mixtures obtained by oxidation of peptides **1** and **2** for 11 days after which they were stirred at 1200 rpm for 6 days. The total peak area remains fairly constant throughout the experiment. Fluctuations can be caused by sample preparation.

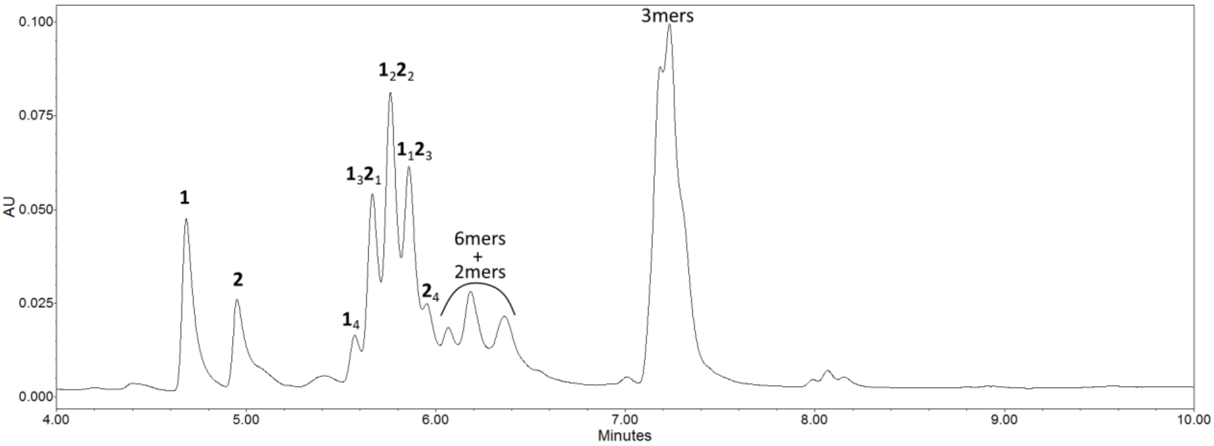

Figure S9. RP-UPLC trace (monitored at 254 nm using method A) of the product mixture obtained by oxidation of peptides **1** and **2** for 11 days after which it was stirred at 1200 rpm for 6 days. This trace corresponds to the data point of the 6mer dominated library after 0 days in **Figure S8**. The total peak area obtained is 3.14*10^{6} AU.

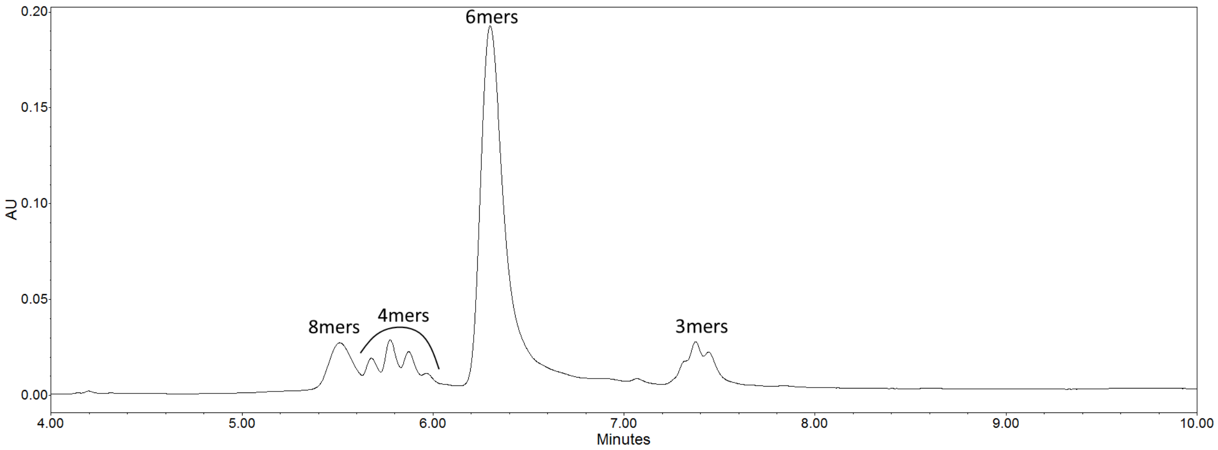

Figure S10. RP-UPLC trace (monitored at 254 nm using method A) of the product mixture obtained by oxidation of peptides **1** and **2** for 11 days after which it was stirred at 1200 rpm for 6 days. This trace corresponds to the data point of the 6mer dominated library after 6 days in **Figure S8**. The total peak area obtained is 3.01*10⁶ AU.

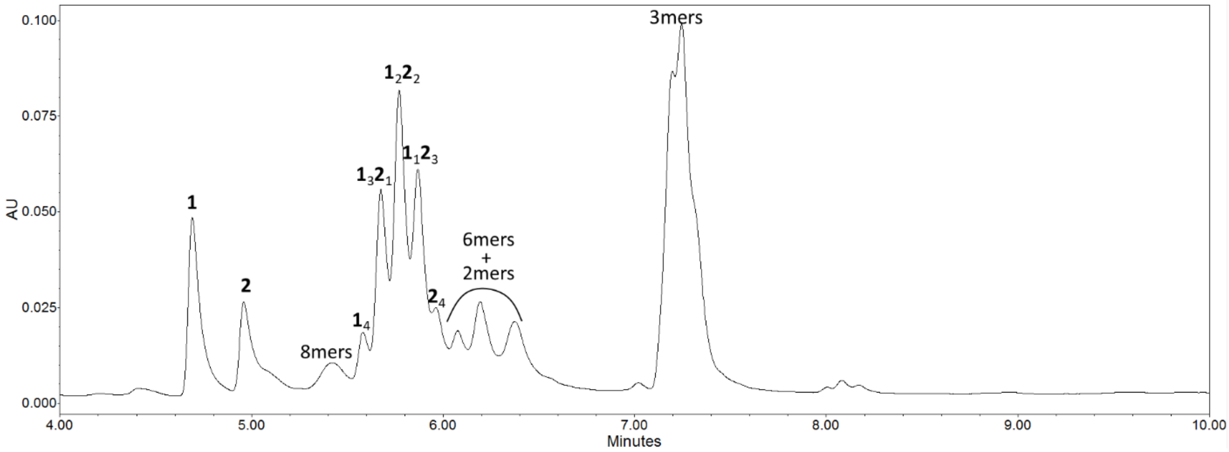

Figure S11. RP-UPLC trace (monitored at 254 nm using method A) of the product mixture obtained by oxidation of peptides **1** and **2** for 11 days after which it was stirred at 1200 rpm for 6 days. This trace corresponds to the data point of the 8mer dominated library after 0 days in **Figure S8**. The total peak area obtained is 3.21*10⁶ AU.

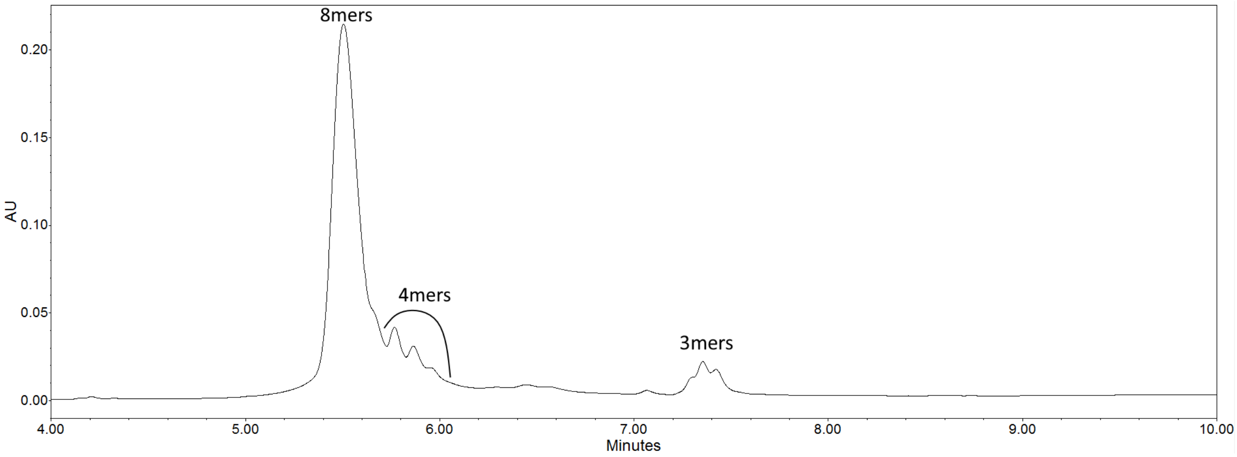

Figure S12. RP-UPLC trace (monitored at 254 nm using method A) of the product mixture obtained by oxidation of peptides **1** and **2** for 11 days after which it was stirred at 1200 rpm for 6 days. This trace corresponds to the data point of the 8mer dominated library after 6 days in **Figure S8**. The total peak area obtained is 3.26*10⁶ AU.

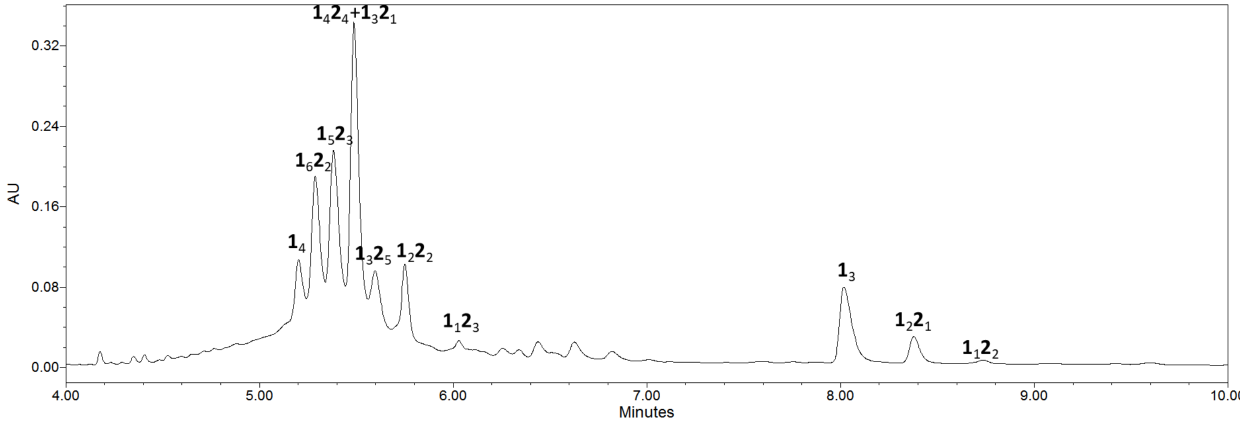

Figure S13. RP-UPLC trace (monitored at 254 nm using method B) of the product mixture obtained by oxidation of 67% peptide **1** and 33% peptide **2** for 12 days after which it was stirred at 1200 rpm for 6 days. The total peak area obtained is 6.88*10^6 AU. Note that when method B was used the DCL was only diluted only fivefold, where a tenfold dilution was used for sample preparation when method A was used.

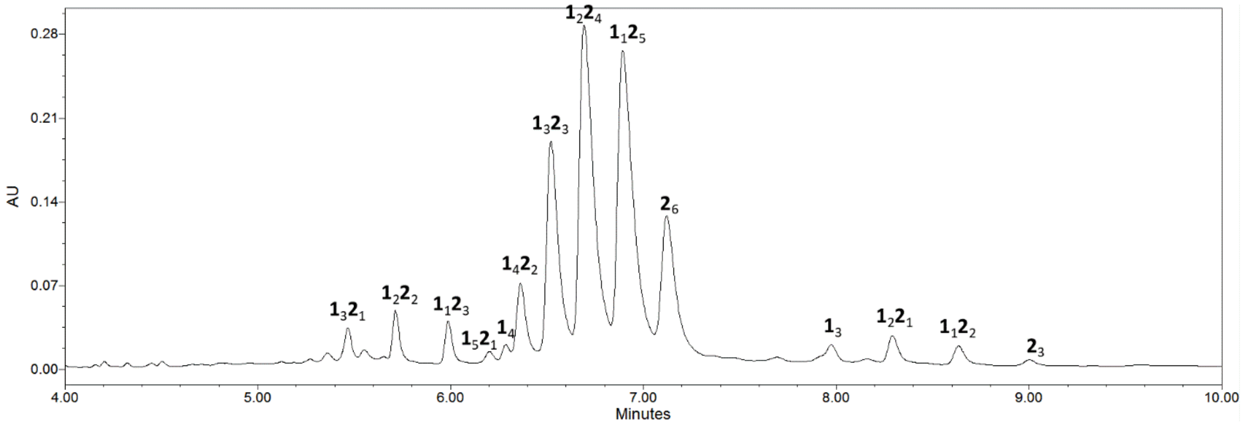

Figure S14. RP-UPLC trace (monitored at 254 nm using method B) of the product mixture obtained by oxidation of 33% peptide **1** and 67% peptide **2** for 12 days after which it was stirred at 1200 rpm for 6 days. The total peak area obtained is 7.06*10^6 AU. Note that when method B was used the DCL was only diluted only fivefold, where a tenfold dilution was used for sample preparation when method A was used.

5. Mass spectrometry

Monomers

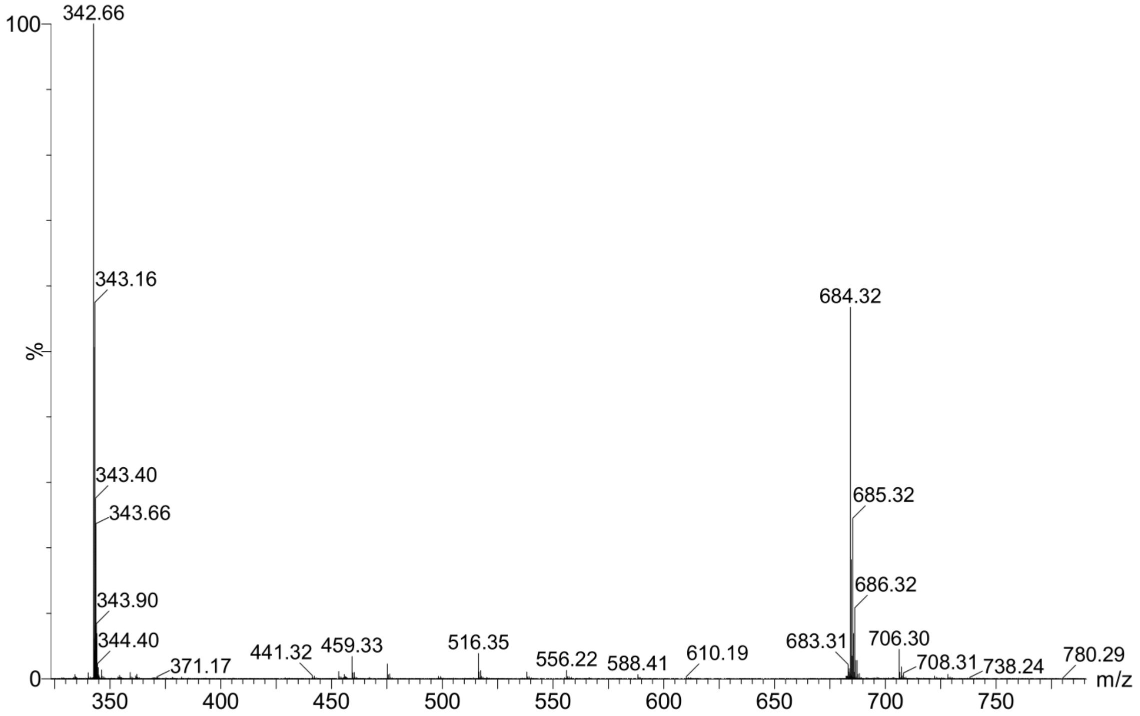

Figure S15. Mass spectrum of monomer **1** from the LC-MS analysis of a stirred library made from peptides **1** and **2** (corresponding to Figure S5). Calculated m/z: 684.31 $[M+1H]^{1+}$, 342.66 $[M+2H]^{2+}$; observed m/z: 684.32 $[M+1H]^{1+}$, 342.66 $[M+2H]^{2+}$

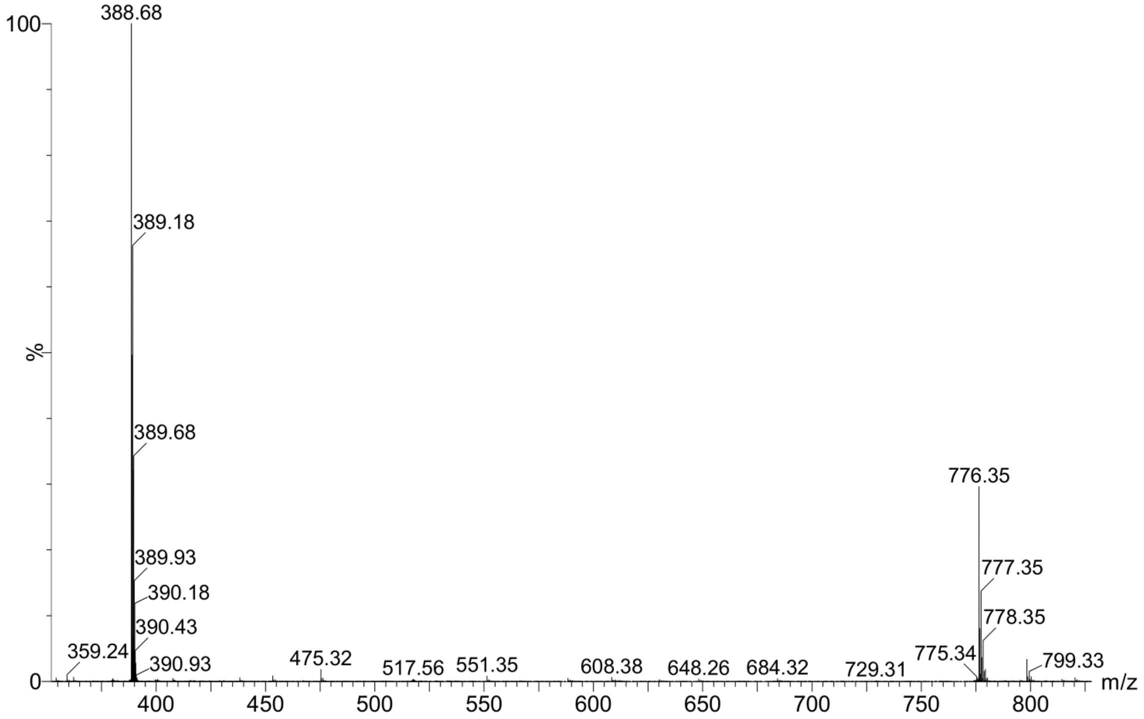

Figure S16. Mass spectrum of monomer **2** from the LC-MS analysis of a stirred library made from peptides **1** and **2** (corresponding to Figure S5). Calculated m/z: 776.34 $[M+1H]^{1+}$, 388.67 $[M+2H]^{2+}$; observed m/z: 776.35 $[M+1H]^{1+}$, 388.68 $[M+2H]^{2+}$

Dimers

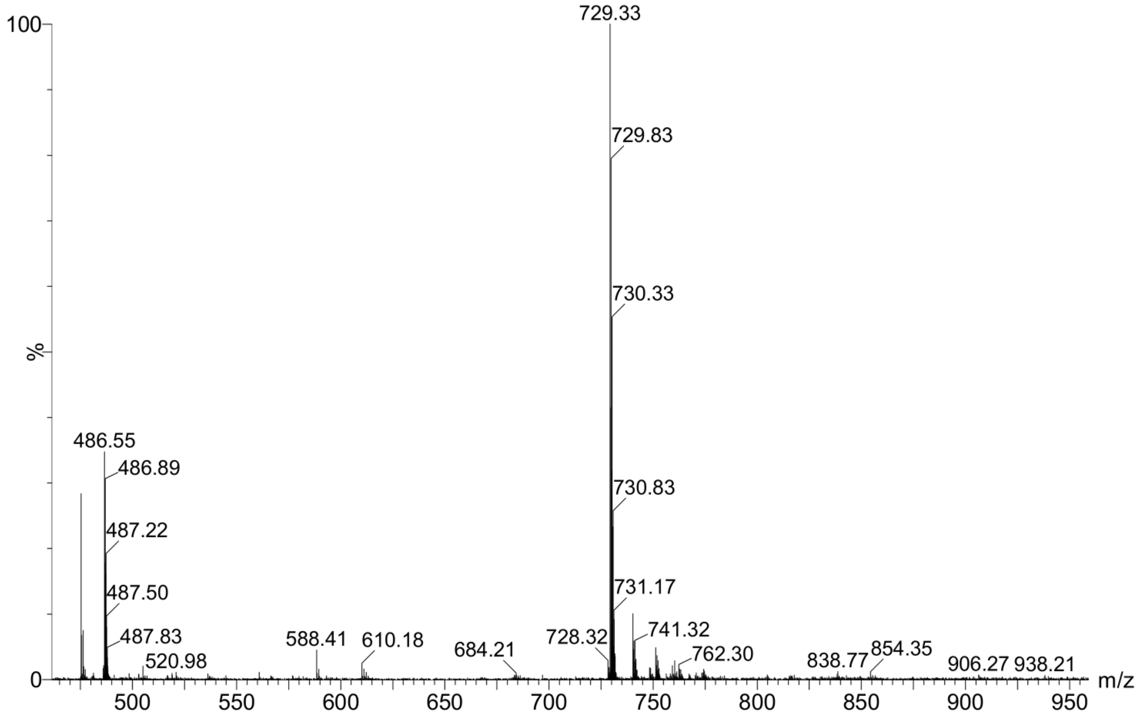

Figure S17. Mass spectrum of dimer $\mathbf{1_1 2_1}$ from the LC-MS analysis of a stirred library made from peptides **1** and **2** (corresponding to Figure S5). Calculated m/z: 729.33 $[M+2H]^{2+}$, 486.55 $[M+3H]^{3+}$; observed m/z: 729.33 $[M+2H]^{2+}$, 486.55 $[M+3H]^{3+}$

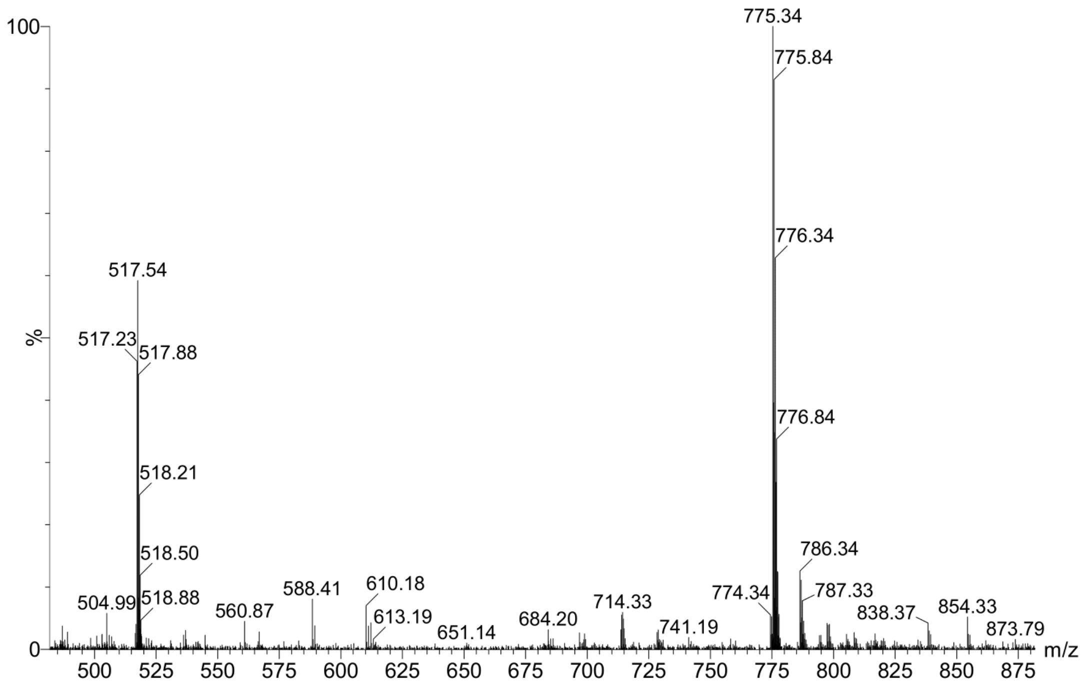

Figure S18. Mass spectrum of dimer $\mathbf{2_2}$ from the LC-MS analysis of a stirred library made from peptides **1** and **2** (corresponding to Figure S5). Calculated m/z: 775.34 $[M+2H]^{2+}$, 517.23 $[M+3H]^{3+}$; observed m/z: 775.34 $[M+2H]^{2+}$, 517.23 $[M+3H]^{3+}$

Trimers

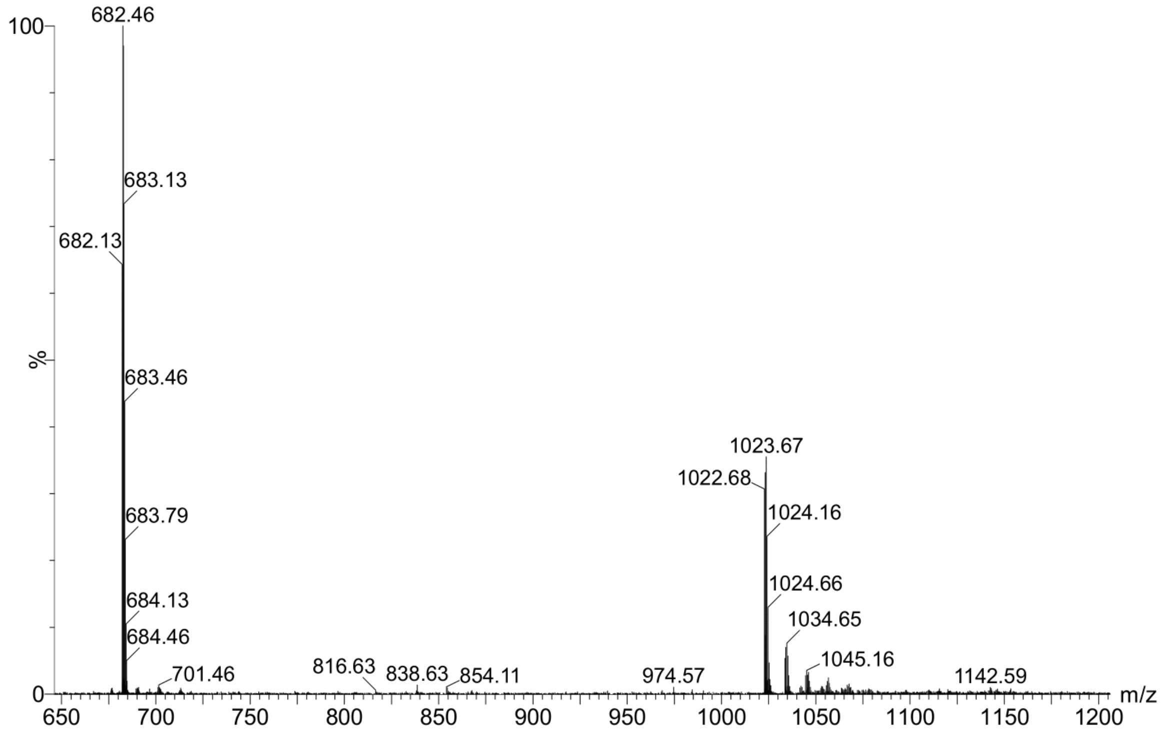

Figure S19. Mass spectrum of trimer $\mathbf{1}_3$ from the LC-MS analysis of a stirred library made from peptides **1** and **2** (corresponding to Figure S5). Calculated m/z: 1022.97 $[M+2H]^{2+}$, 682.31 $[M+3H]^{3+}$; observed m/z: 1022.68 $[M+2H]^{2+}$, 682.13 $[M+3H]^{3+}$

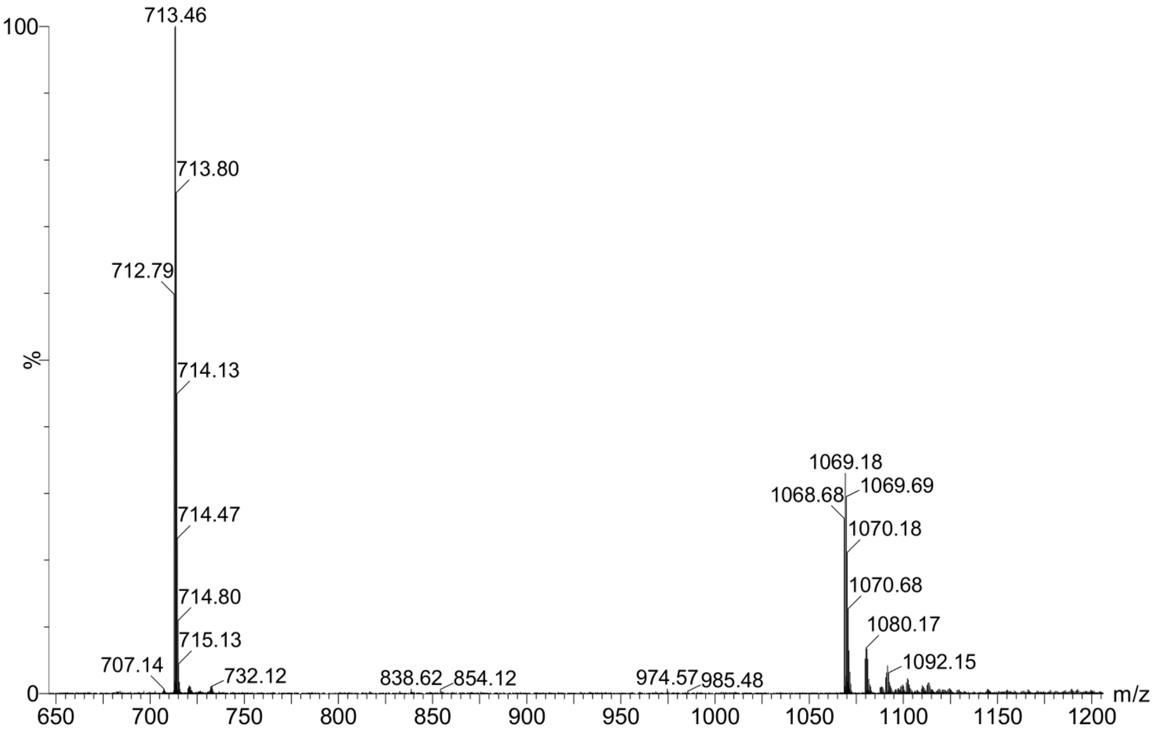

Figure S20. Mass spectrum of trimer $\mathbf{1}_2\mathbf{2}_1$ from the LC-MS analysis of a stirred library made from peptides **1** and **2** (corresponding to Figure S5). Calculated m/z: 1068.98 $[M+2H]^{2+}$, 712.99 $[M+3H]^{3+}$; observed m/z: 1068.68 $[M+2H]^{2+}$, 712.79 $[M+3H]^{3+}$

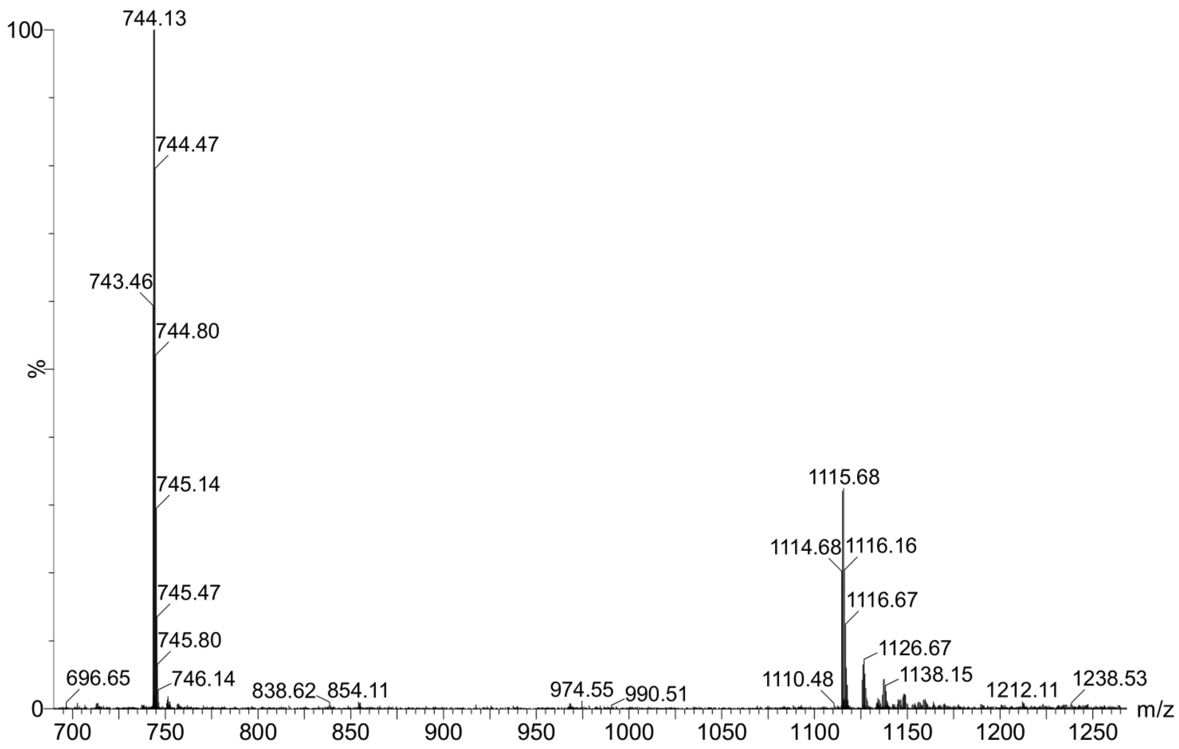

Figure S21. Mass spectrum of trimer $\mathbf{1_1 2_2}$ from the LC-MS analysis of a stirred library made from peptides **1** and **2** (corresponding to Figure S5). Calculated m/z: 1115.00 $[M+2H]^{2+}$, 743.67 $[M+3H]^{3+}$; observed m/z: 1114.68 $[M+2H]^{2+}$, 743.46 $[M+3H]^{3+}$

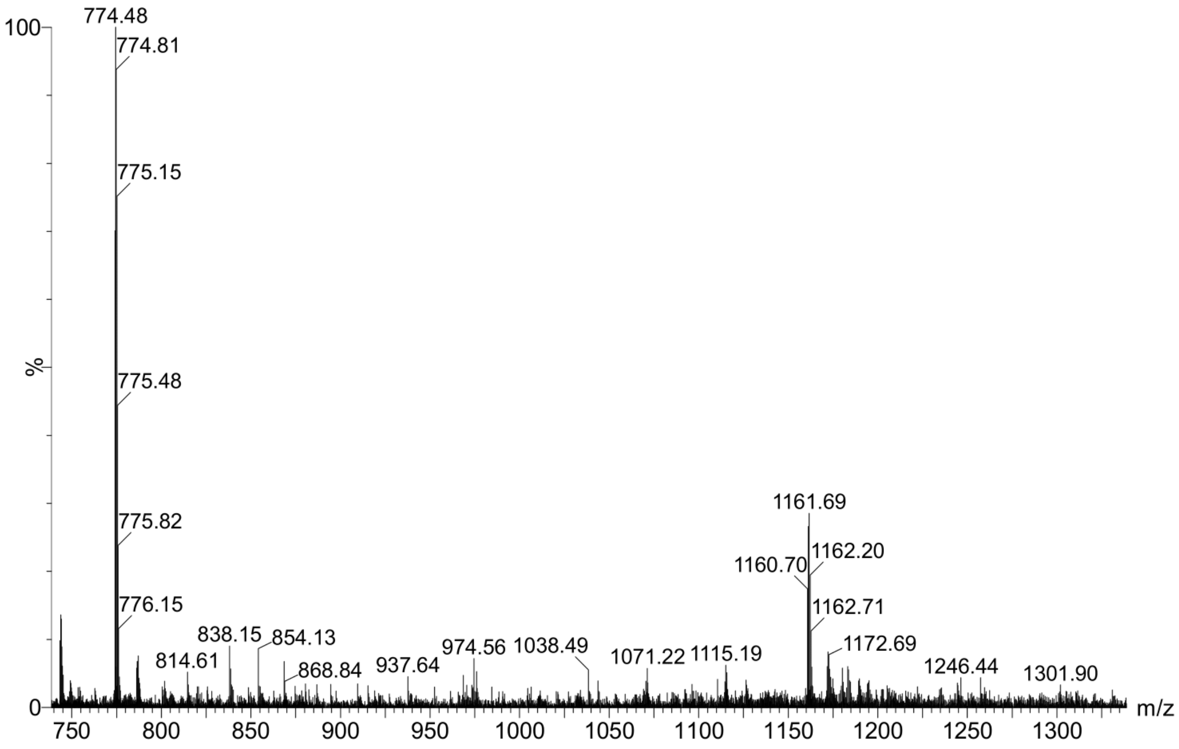

Figure S22. Mass spectrum of trimer $\mathbf{2_3}$ from the LC-MS analysis of a stirred library made from peptides **1** and **2** (corresponding to Figure S5). Calculated m/z: 1161.01 $[M+2H]^{2+}$, 774.34 $[M+3H]^{3+}$; observed m/z: 1160.70 $[M+2H]^{2+}$, 774.14 $[M+3H]^{3+}$

Tetramers

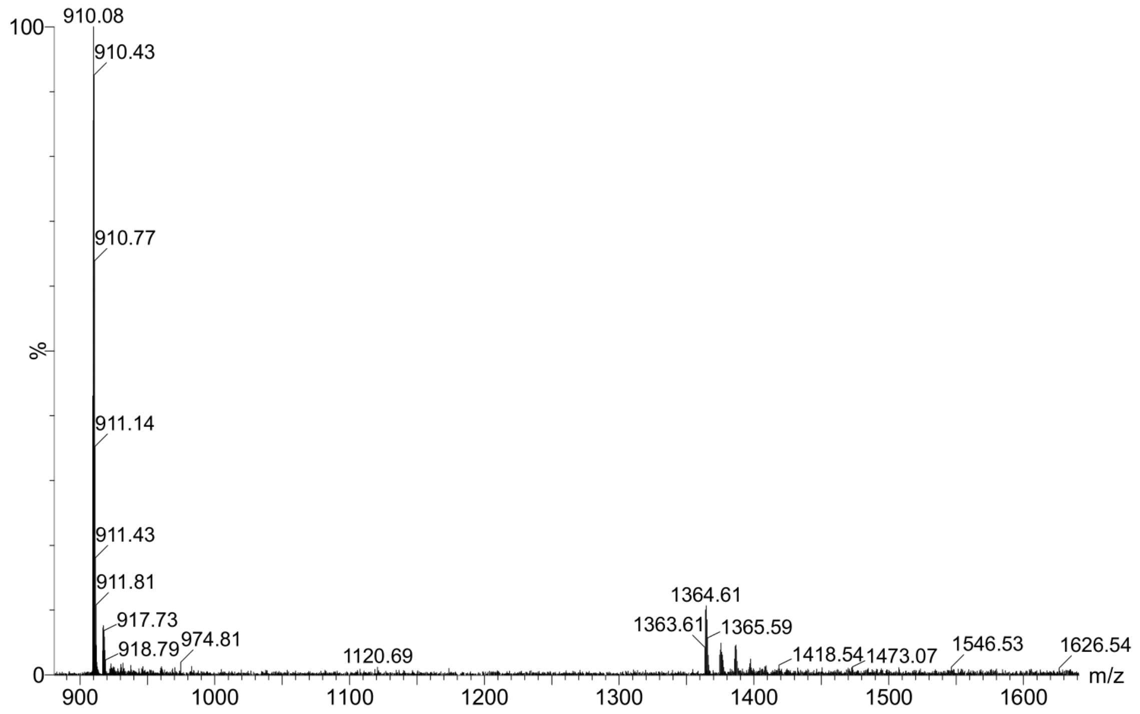

Figure S23. Mass spectrum of tetramer $\mathbf{1_4}$ from the LC-MS analysis of a stirred library made from peptides **1** and **2** (corresponding to Figure S5). Calculated m/z: 1363.62 $[M+2H]^{2+}$, 909.41 $[M+3H]^{3+}$; observed m/z: 1363.61 $[M+2H]^{2+}$, 909.41 $[M+3H]^{3+}$

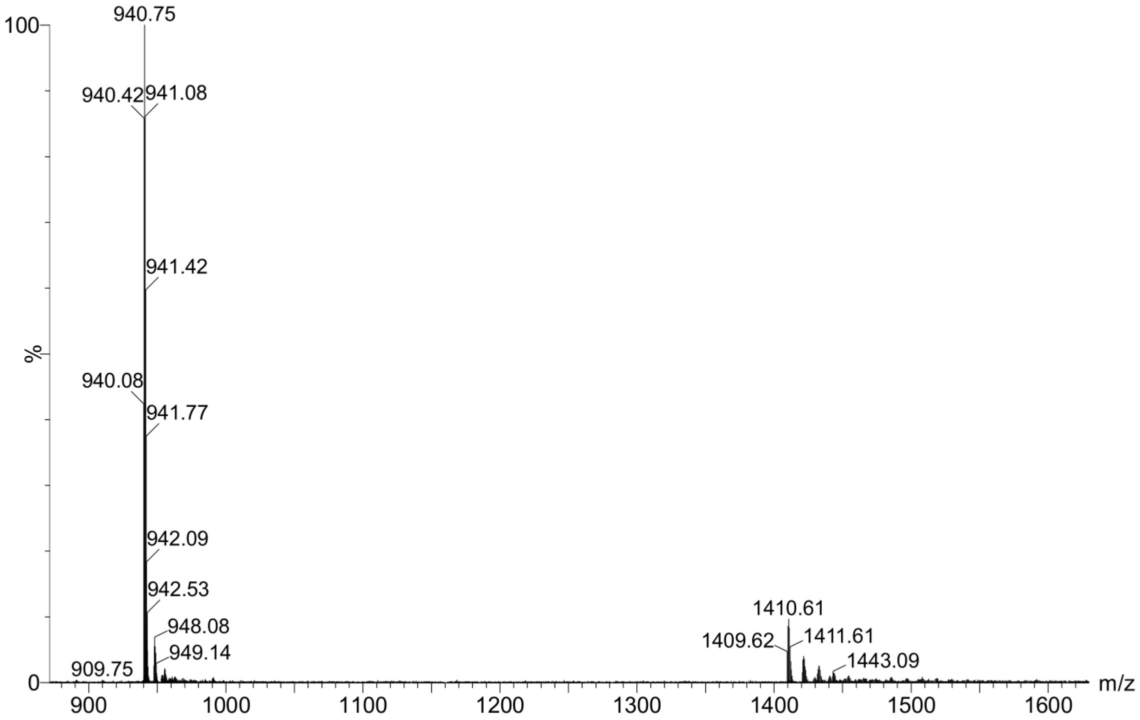

Figure S24. Mass spectrum of tetramer $\mathbf{1_3 2_1}$ from the LC-MS analysis of a stirred library made from peptides **1** and **2** (corresponding to Figure S5). Calculated m/z: 1409.64 $[M+2H]^{2+}$, 940.09 $[M+3H]^{3+}$; observed m/z: 1409.62 $[M+2H]^{2+}$, 940.08 $[M+3H]^{3+}$

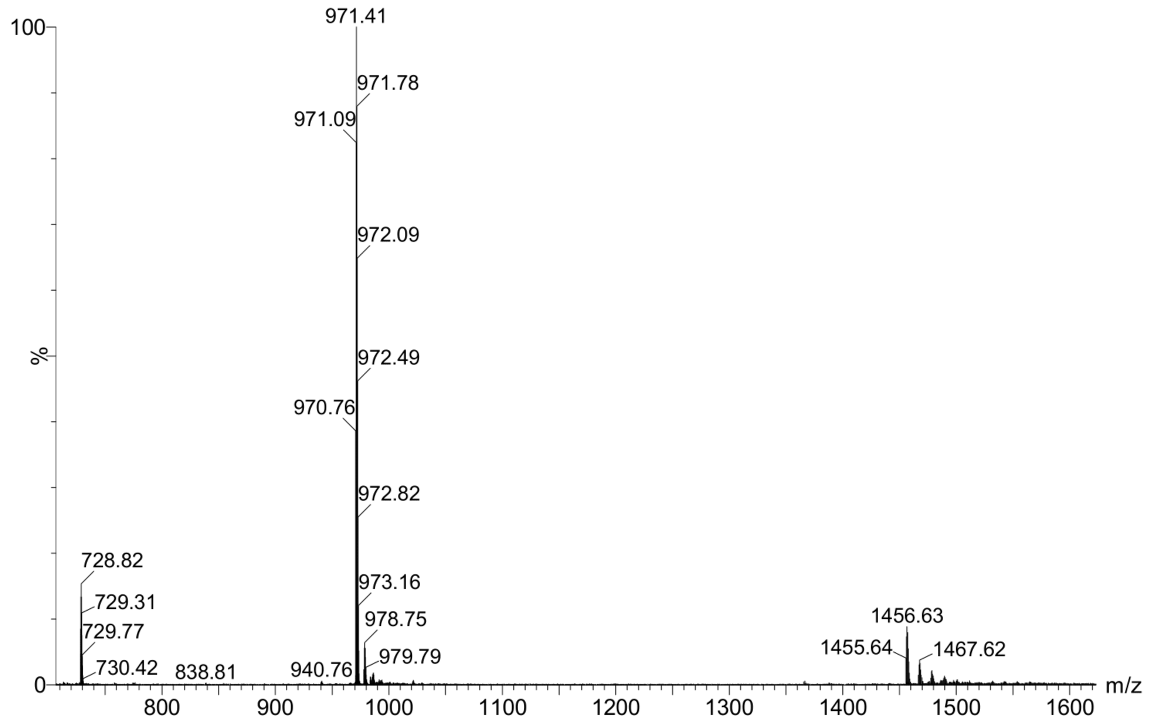

Figure S25. Mass spectrum of tetramer $\mathbf{1}_2\mathbf{2}_2$ from the LC-MS analysis of a stirred library made from peptides **1** and **2** (corresponding to Figure S5). Calculated m/z: 1455.65 $[M+2H]^{2+}$, 970.77 $[M+3H]^{3+}$, 728.33 $[M+4H]^{4+}$; observed m/z: 1455.64 $[M+2H]^{2+}$, 970.76 $[M+3H]^{3+}$, 728.32 $[M+4H]^{4+}$

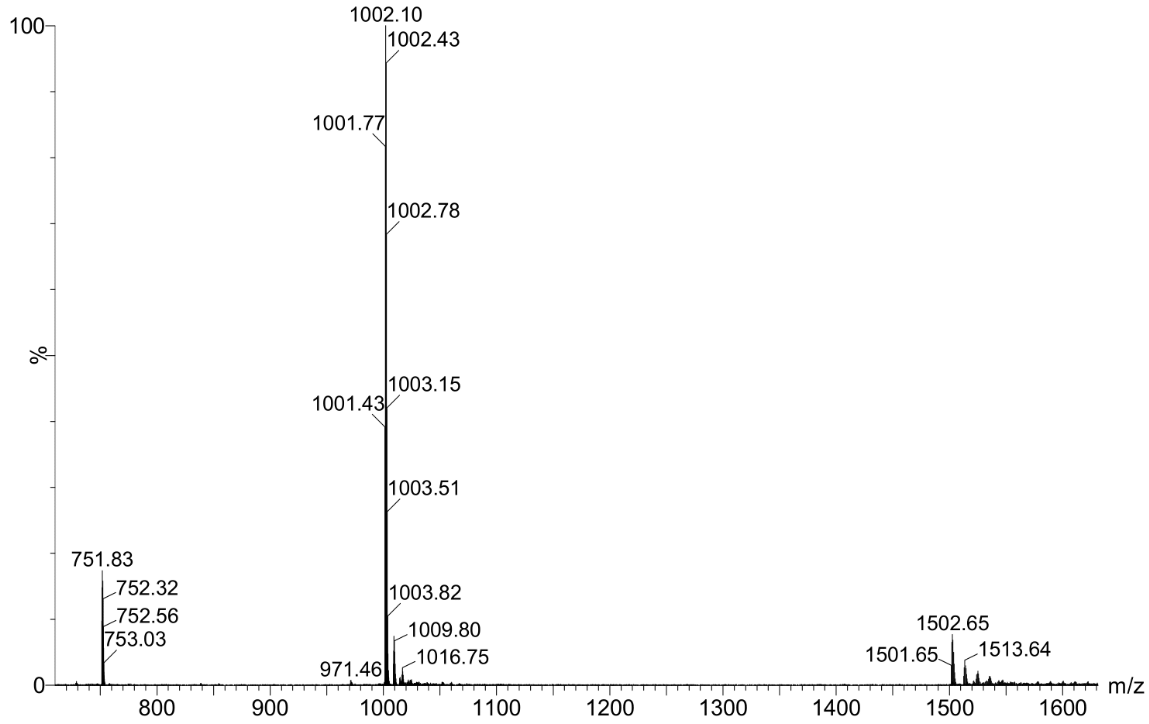

Figure S26. Mass spectrum of tetramer $\mathbf{1}_1\mathbf{2}_3$ from the LC-MS analysis of a stirred library made from peptides **1** and **2** (corresponding to Figure S5). Calculated m/z: 1501.65 $[M+2H]^{2+}$, 1001.43 $[M+3H]^{3+}$, 751.33 $[M+4H]^{4+}$; observed m/z: 1501.67 $[M+2H]^{2+}$, 1001.44 $[M+3H]^{3+}$, 751.33 $[M+4H]^{4+}$

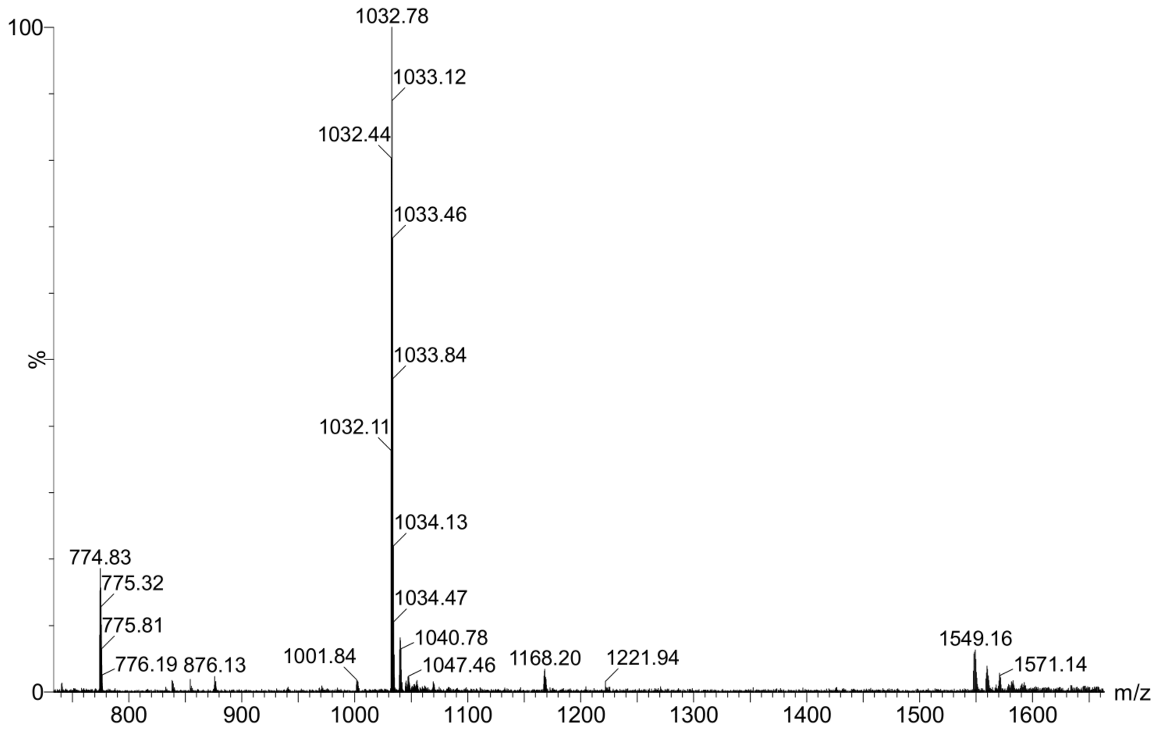

Figure S27. Mass spectrum of tetramer **2**$_4$ from the LC-MS analysis of a stirred library made from peptides **1** and **2** (corresponding to Figure S5). Calculated m/z: 1547.68 $[M+2H]^{2+}$, 1032.12 $[M+3H]^{3+}$, 774.34 $[M+4H]^{4+}$; observed m/z: 1547.65 $[M+2H]^{2+}$, 1032.11 $[M+3H]^{3+}$, 774.33 $[M+4H]^{4+}$

Pentamers

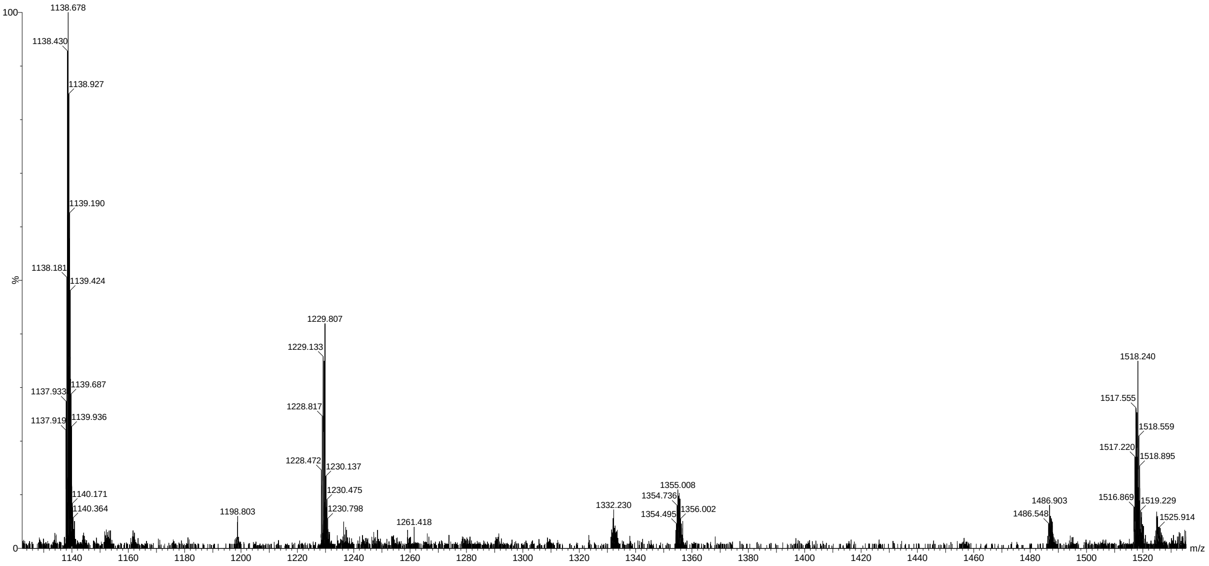

Figure S28. Mass spectrum of pentamer $\mathbf{1}_2\mathbf{2}_3$ and hexamer $\mathbf{1}_1\mathbf{2}_5$ from the LC-MS analysis of a stirred library made from 15 mol% **1** and 85 mol% **2** (corresponding to Figure S7).

$\mathbf{1}_1\mathbf{2}_5$: Calculated m/z: 1517.00 $[M+3H]^{3+}$, 1138.00 $[M+4H]^{4+}$;
observed m/z: 1516.87 $[M+3H]^{3+}$, 1137.92 $[M+4H]^{4+}$

$\mathbf{1}_2\mathbf{2}_3$: Calculated m/z: 1228.55 $[M+4H]^{4+}$; observed m/z: 1228.47 $[M+4H]^{4+}$

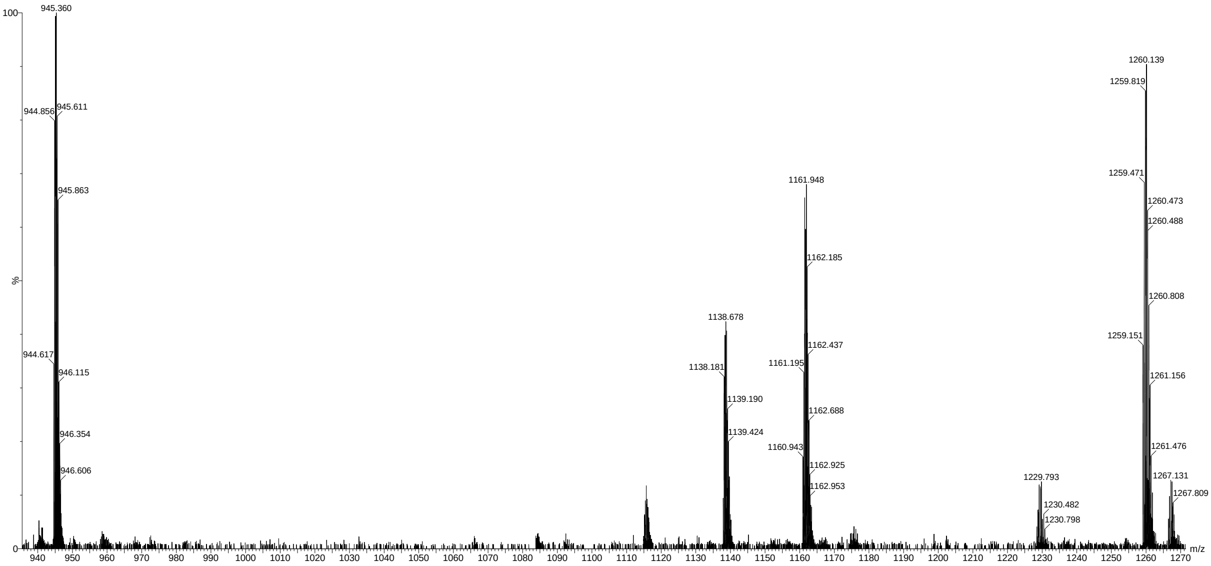

Figure S29. Mass spectrum of pentamer $\mathbf{1}_1\mathbf{2}_4$ and hexamers $\mathbf{1}_1\mathbf{2}_5$ and $\mathbf{2}_6$ from the LC-MS analysis of a stirred library made from 15 mol% **1** and 85 mol% **2** (corresponding to Figure S7).

$\mathbf{1}_1\mathbf{2}_4$: Calculated m/z: 1259.22 $[M+3H]^{3+}$, 944.67 $[M+4H]^{4+}$; observed m/z: 1259.15 $[M+3H]^{3+}$, 944.62 $[M+4H]^{4+}$

$\mathbf{1}_1\mathbf{2}_5$: Calculated m/z: 1138.00 $[M+4H]^{4+}$; observed m/z: 1138.18 $[M+4H]^{4+}$

$\mathbf{2}_6$: Calculated m/z: 1161.01 $[M+4H]^{4+}$; observed m/z: 1160.94 $[M+4H]^{4+}$

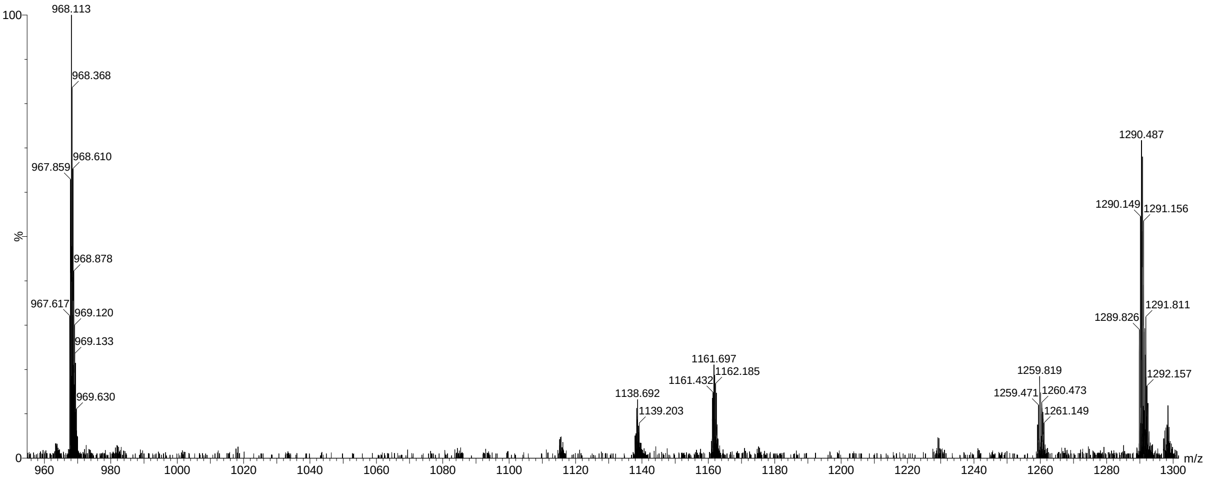

Figure S30. Mass spectrum of pentamer $\mathbf{2}_5$ from the LC-MS analysis of a stirred library made from 15 mol% **1** and 85 mol% **2** (corresponding to Figure S7). Calculated m/z: 1289.90 $[M+3H]^{3+}$, 967.68 $[M+4H]^{4+}$; observed m/z: 1289.83 $[M+3H]^{3+}$, 967.62 $[M+4H]^{4+}$

Hexamers

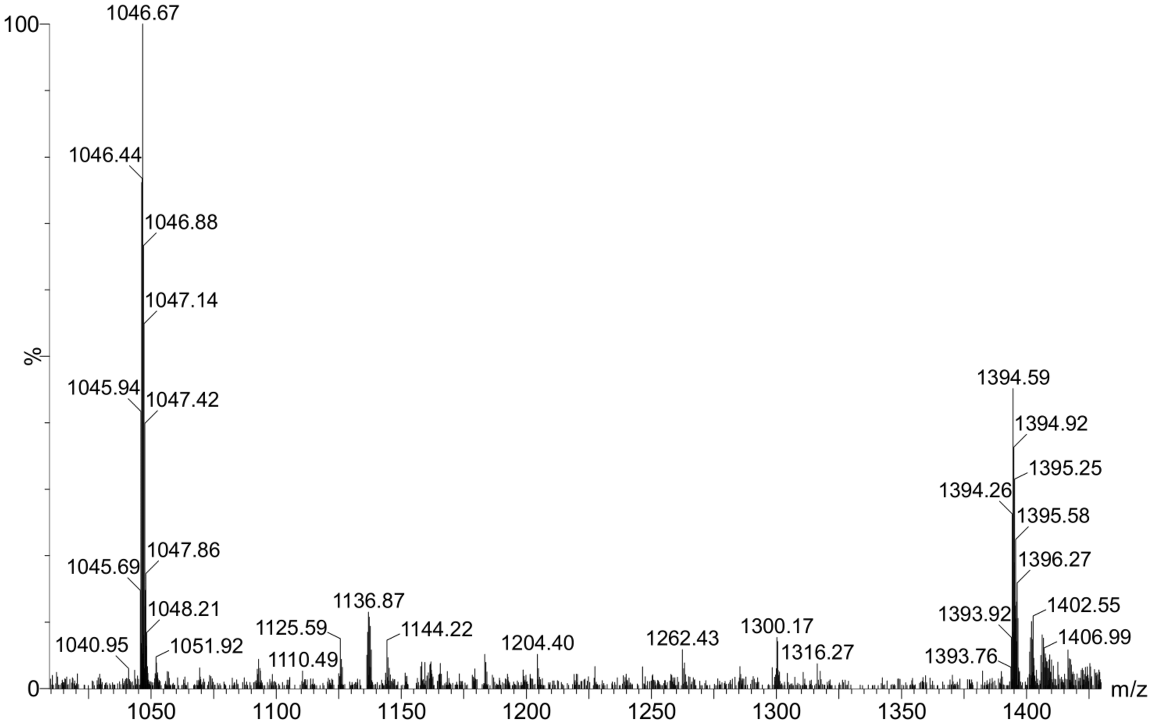

Figure S31. Mass spectrum of hexamer $\mathbf{1}_5\mathbf{2}_1$ from the LC-MS analysis of a stirred library made from peptides **1** and **2** (corresponding to Figure S6). Calculated m/z: 1394.30 $[M+3H]^{3+}$, 1045.97 $[M+4H]^{4+}$; observed m/z: 1394.26 $[M+3H]^{3+}$, 1045.69 $[M+4H]^{4+}$

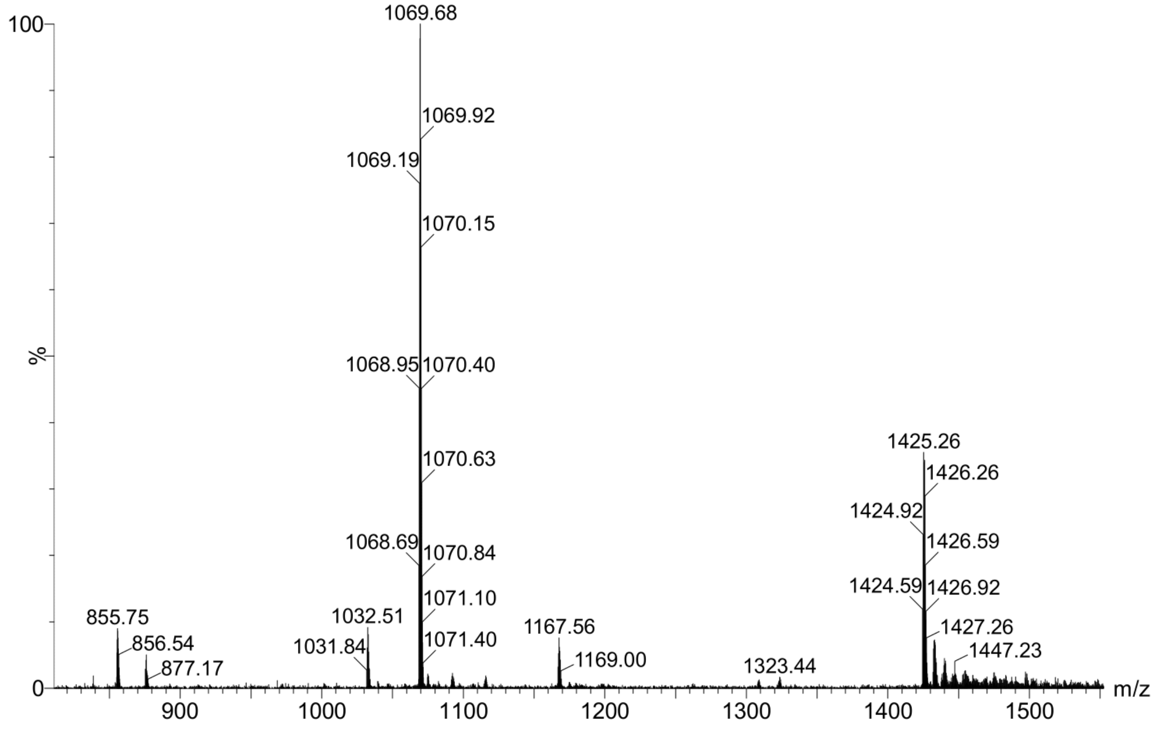

Figure S32. Mass spectrum of hexamer $\mathbf{1}_4\mathbf{2}_2$ from the LC-MS analysis of a stirred library made from peptides **1** and **2** (corresponding to Figure S6). Calculated m/z: 1424.97 $[M+3H]^{3+}$, 1068.98 $[M+4H]^{4+}$, 855.38 $[M+5H]^{5+}$; observed m/z: 1424.59 $[M+3H]^{3+}$, 1068.69 $[M+4H]^{4+}$, 855.36 $[M+5H]^{5+}$

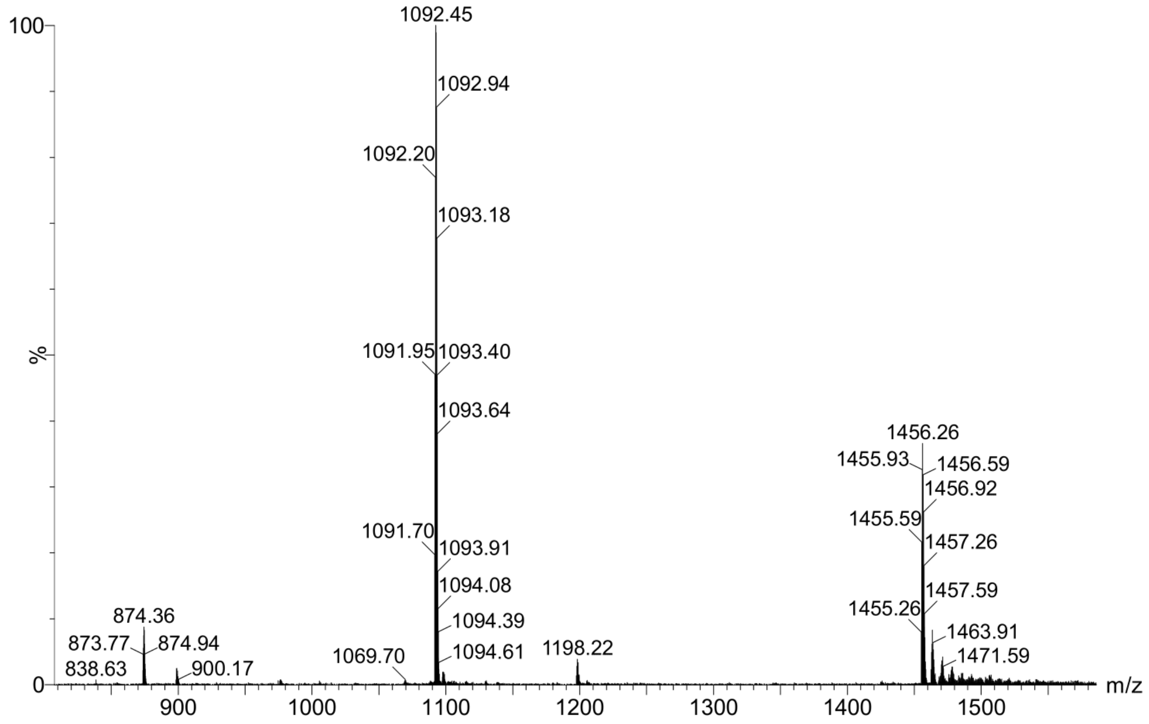

Figure S33. Mass spectrum of hexamer $\mathbf{1}_3\mathbf{2}_3$ from the LC-MS analysis of a stirred library made from peptides **1** and **2** (corresponding to Figure S6). Calculated m/z: 1455.65 $[M+3H]^{3+}$, 1091.99 $[M+4H]^{4+}$, 873.79 $[M+5H]^{5+}$; observed m/z: 1455.26 $[M+3H]^{3+}$, 1091.70 $[M+4H]^{4+}$, 873.56 $[M+5H]^{5+}$

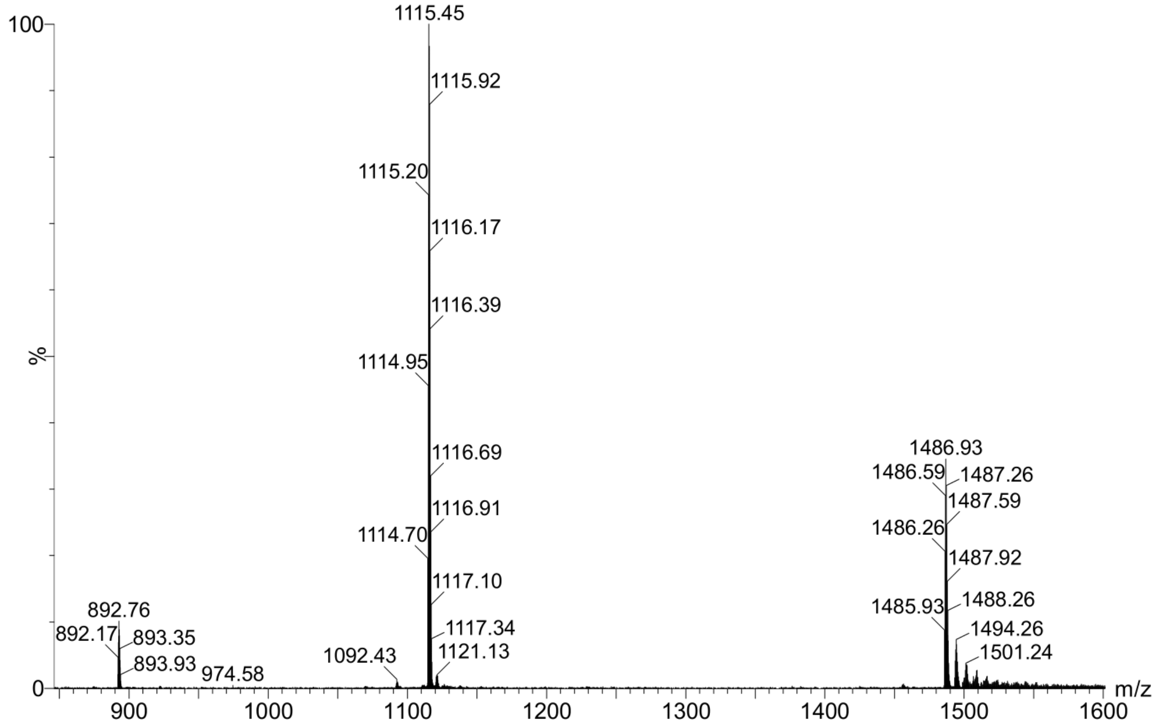

Figure S34. Mass spectrum of hexamer $\mathbf{1}_2\mathbf{2}_4$ from the LC-MS analysis of a stirred library made from peptides **1** and **2** (corresponding to Figure S6). Calculated m/z: 1486.33 $[M+3H]^{3+}$, 1115.00 $[M+4H]^{4+}$, 892.20 $[M+5H]^{5+}$; observed m/z: 1485.93 $[M+3H]^{3+}$, 1114.70 $[M+4H]^{4+}$, 891.96 $[M+5H]^{5+}$

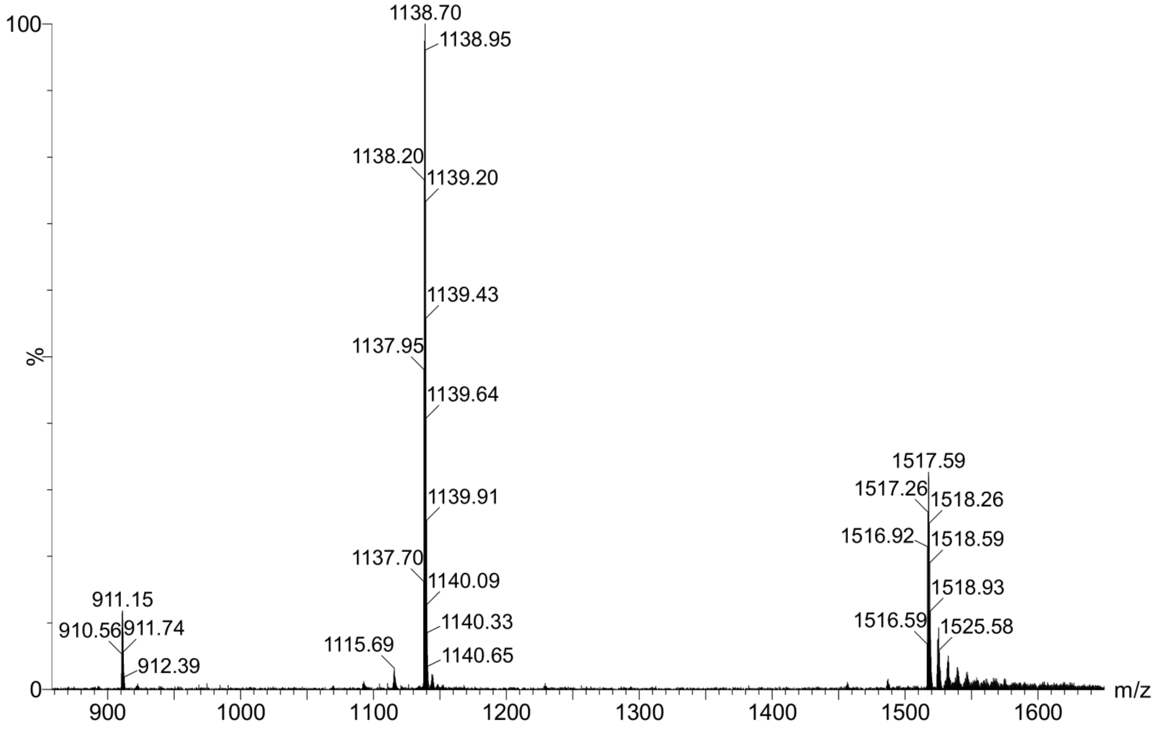

Figure S35. Mass spectrum of hexamer $\mathbf{1}_1\mathbf{2}_5$ from the LC-MS analysis of a stirred library made from peptides **1** and **2** (corresponding to Figure S6). Calculated m/z: 1517.00 $[M+3H]^{3+}$, 1138.00 $[M+4H]^{4+}$, 910.60 $[M+5H]^{5+}$; observed m/z: 1516.59 $[M+3H]^{3+}$, 1137.70 $[M+4H]^{4+}$, 910.35 $[M+5H]^{5+}$

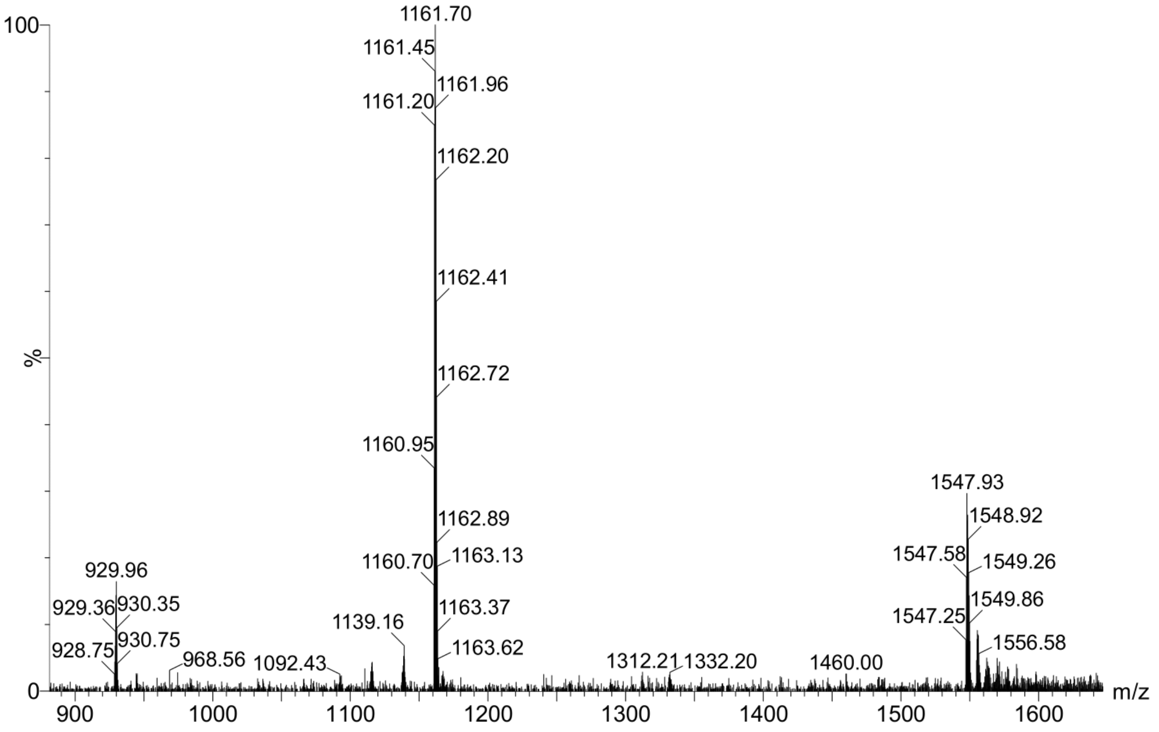

Figure S36. Mass spectrum of hexamer $\mathbf{2}_6$ from the LC-MS analysis of a stirred library made from peptides **1** and **2** (corresponding to Figure S6). Calculated m/z: 1547.68 $[M+3H]^{3+}$, 1161.01 $[M+4H]^{4+}$, 929.01 $[M+5H]^{5+}$; observed m/z: 1547.25 $[M+3H]^{3+}$, 1160.70 $[M+4H]^{4+}$, 928.75 $[M+5H]^{5+}$

Heptamers

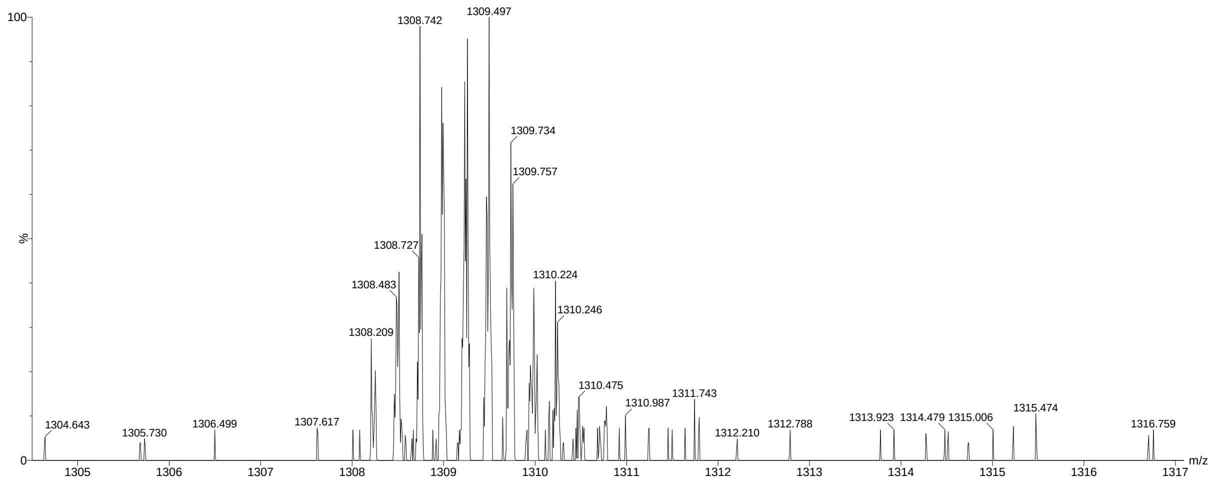

Figure S37. Mass spectrum of heptamer $\mathbf{1}_2\mathbf{2}_5$ from the LC-MS analysis of a stirred library made from 15 mol% **1** and 85 mol% **2** (corresponding to Figure S7). $\mathbf{1}_2\mathbf{2}_5$: Calculated m/z: 1308.33 $[M+4H]^{4+}$; observed m/z: 1308.21 $[M+4H]^{4+}$

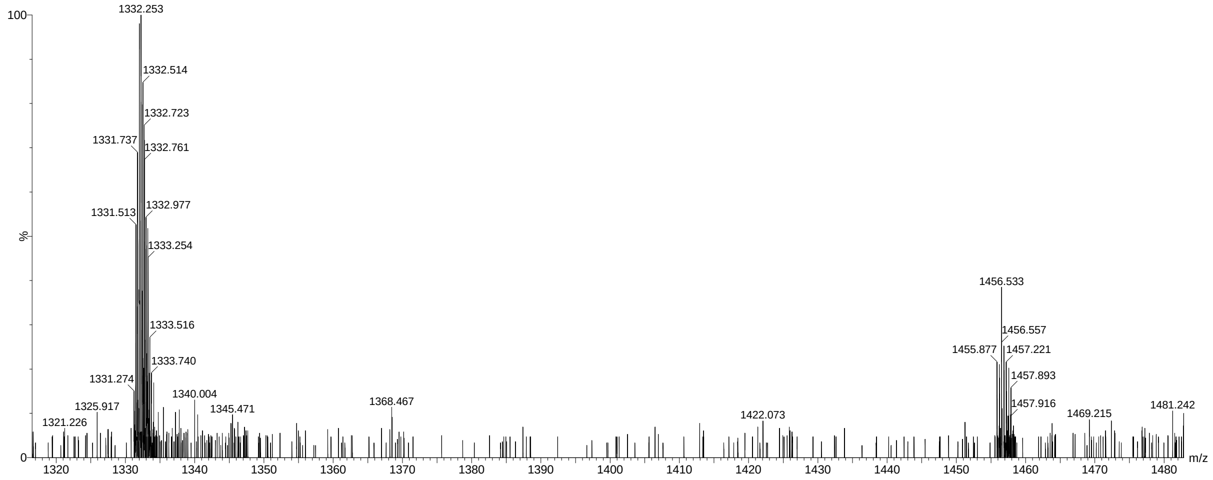

Figure S38. Mass spectrum of hexamer $\mathbf{1}_3\mathbf{2}_3$ and heptamer $\mathbf{1}_1\mathbf{2}_6$ from the LC-MS analysis of a stirred library made from 15 mol% **1** and 85 mol% **2** (corresponding to Figure S7).

$\mathbf{1}_3\mathbf{2}_3$: Calculated m/z: 1455.65 $[M+4H]^{4+}$; observed m/z: 1455.88 $[M+4H]^{4+}$

$\mathbf{1}_1\mathbf{2}_6$: Calculated m/z: 1331.34 $[M+4H]^{4+}$; observed m/z: 1331.27 $[M+4H]^{4+}$

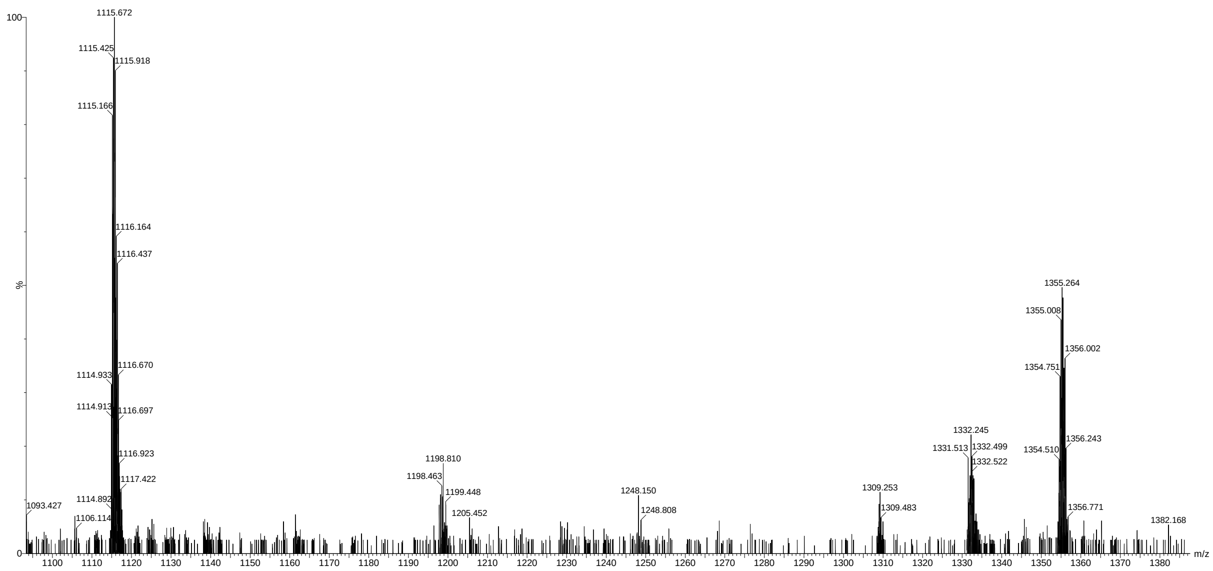

Figure S39. Mass spectrum of hexamer $\mathbf{1}_2\mathbf{2}_4$ and heptamers $\mathbf{1}_1\mathbf{2}_6$ and $\mathbf{2}_7$ from the LC-MS analysis of a stirred library made from 15 mol% **1** and 85 mol% **2** (corresponding to Figure S7).
$\mathbf{1}_2\mathbf{2}_4$: Calculated m/z: 1115.00 [M+4H]$^{4+}$; observed m/z: 1114.91 [M+4H]$^{4+}$
$\mathbf{1}_1\mathbf{2}_6$: Calculated m/z: 1331.34 [M+4H]$^{4+}$; observed m/z: 1331.51 [M+4H]$^{4+}$
$\mathbf{2}_7$: Calculated m/z: 1354.35 [M+4H]$^{4+}$; observed m/z: 1354.51 [M+4H]$^{4+}$

Octamers

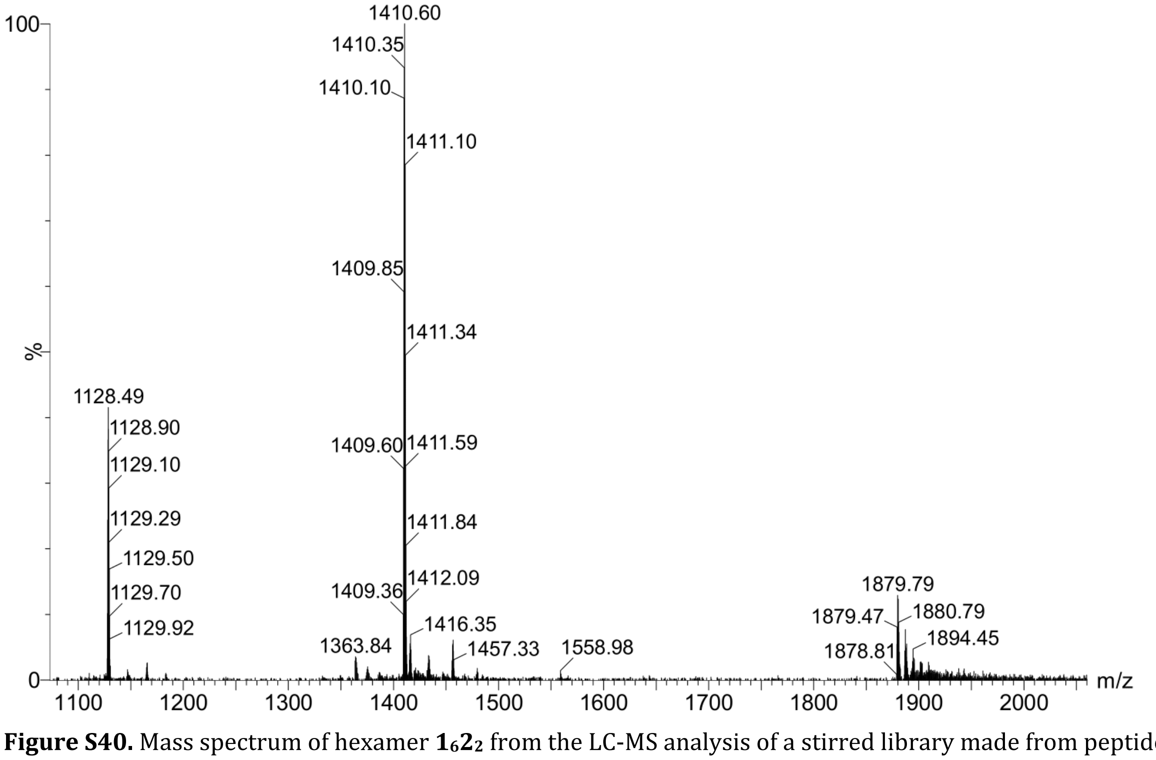

Figure S40. Mass spectrum of hexamer $\mathbf{1}_6\mathbf{2}_2$ from the LC-MS analysis of a stirred library made from peptides **1** and **2** (corresponding to Figure S6). Calculated m/z: 1879.18 [M+3H]$^{3+}$, 1409.64 [M+4H]$^{4+}$, 1127.91 [M+5H]$^{5+}$; observed m/z: 1879.13 [M+3H]$^{3+}$, 1409.36 [M+4H]$^{4+}$, 1127.88 [M+5H]$^{5+}$

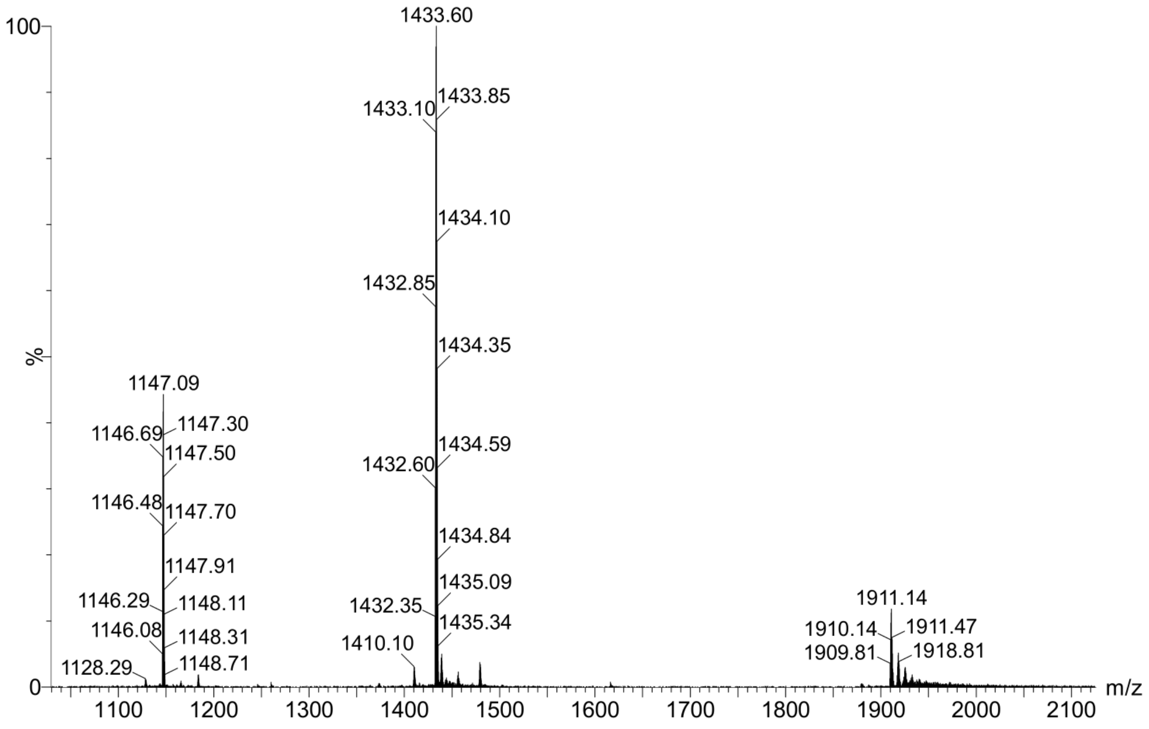

Figure S41. Mass spectrum of hexamer $\mathbf{1}_5\mathbf{2}_3$ from the LC-MS analysis of a stirred library made from peptides **1** and **2** (corresponding to Figure S6). Calculated m/z: 1909.86 $[M+3H]^{3+}$, 1432.64 $[M+4H]^{4+}$, 1146.31 $[M+5H]^{5+}$; observed m/z: 1909.81 $[M+3H]^{3+}$, 1432.60 $[M+4H]^{4+}$, 1146.29 $[M+5H]^{5+}$

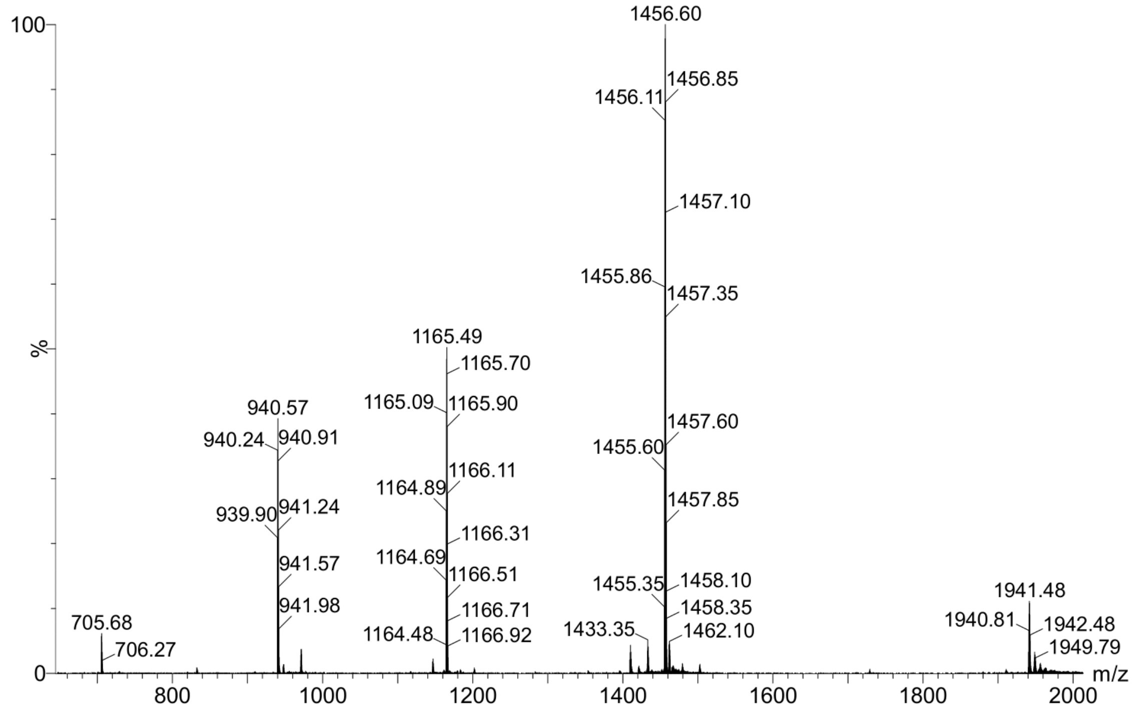

Figure S42. Mass spectrum of hexamer $\mathbf{1}_4\mathbf{2}_4$ from the LC-MS analysis of a stirred library made from peptides **1** and **2** (corresponding to Figure S6). Calculated m/z: 1940.53 $[M+3H]^{3+}$, 1455.65 $[M+4H]^{4+}$, 1164.72 $[M+5H]^{5+}$; observed m/z: 1940.48 $[M+3H]^{3+}$, 1455.60 $[M+4H]^{4+}$, 1164.69 $[M+5H]^{5+}$. The peaks at 705 m/z and 940 m/z correspond the tetramer $\mathbf{1}_3\mathbf{2}_1$. Calculated m/z: 940.09 $[M+3H]^{3+}$, 705.32 $[M+4H]^{4+}$; observed m/z: 939.90 $[M+3H]^{3+}$, 705.18 $[M+4H]^{4+}$

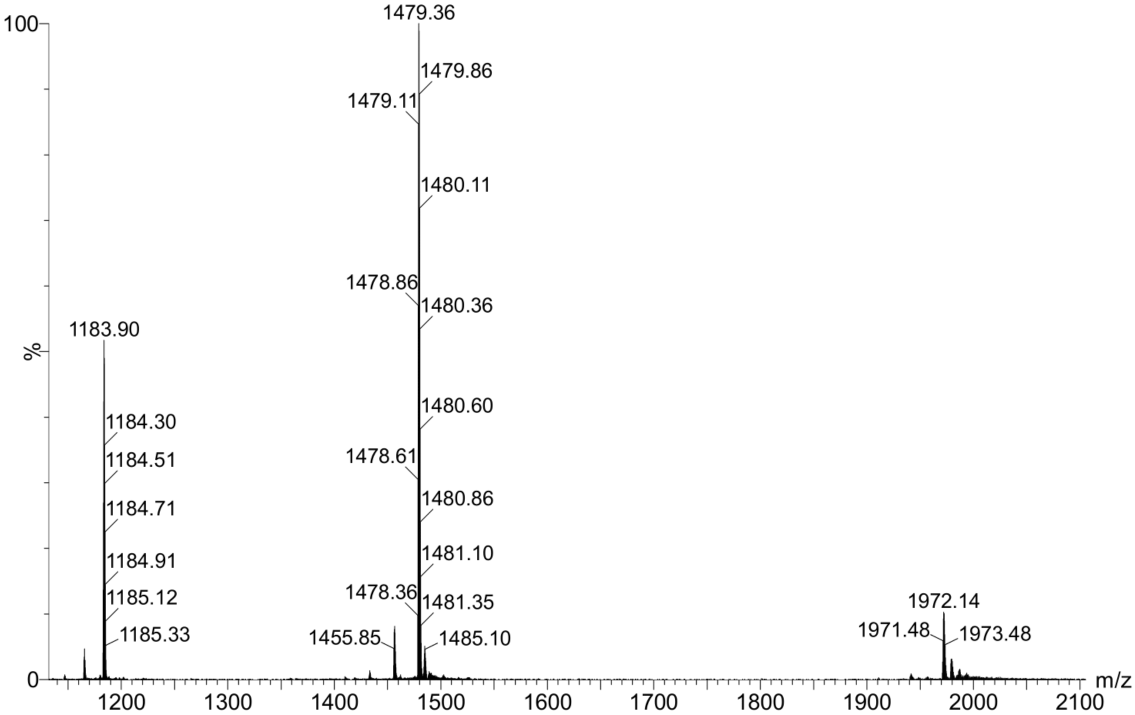

Figure S43. Mass spectrum of hexamer $\mathbf{1}_3\mathbf{2}_5$ from the LC-MS analysis of a stirred library made from peptides **1** and **2** (corresponding to Figure S6). Calculated m/z: 1971.21 [M+3H]$^{3+}$, 1478.66 [M+4H]$^{4+}$, 1183.13 [M+5H]$^{5+}$; observed m/z: 1971.15 [M+3H]$^{3+}$, 1478.61 [M+4H]$^{4+}$, 1183.09 [M+5H]$^{5+}$

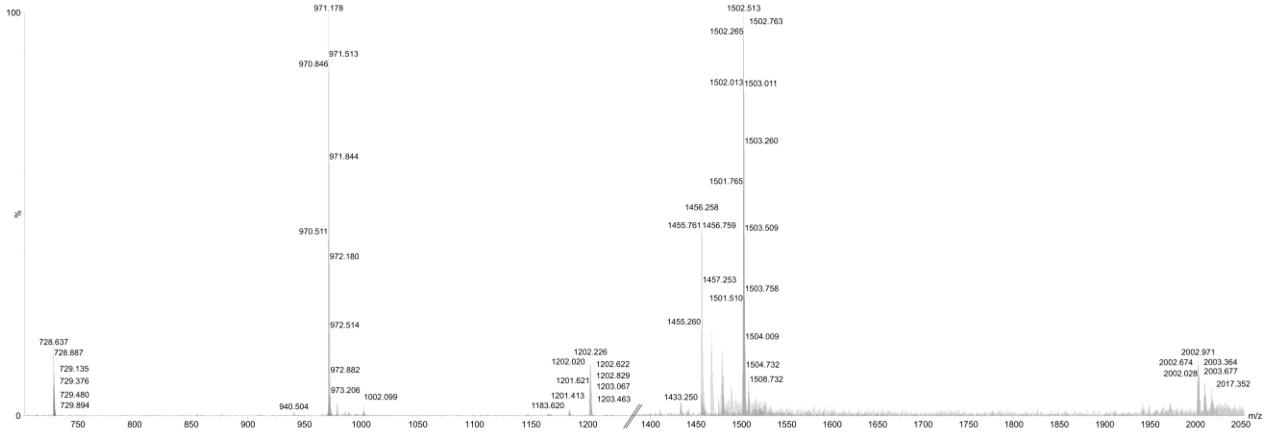

Figure S44. Mass spectrum of hexamer $\mathbf{1}_2\mathbf{2}_6$ from the LC-MS analysis of a stirred library made from peptides **1** and **2** (corresponding to Figure S6). Calculated m/z: 2001.89 [M+3H]$^{3+}$, 1501.67 [M+4H]$^{4+}$, 1201.53 [M+5H]$^{5+}$; observed m/z: 2002.03 [M+3H]$^{3+}$, 1501.51 [M+4H]$^{4+}$, 1201.41 [M+5H]$^{5+}$.
The peaks at 728 m/z, 970 m/z and 1456 m/z correspond the tetramer $\mathbf{1}_2\mathbf{2}_2$. Calculated m/z: 1455.65 [M+2H]$^{2+}$, 970.77 [M+3H]$^{3+}$, 728.33 [M+4H]$^{4+}$; observed m/z: 1455.26 [M+2H]$^{2+}$, 970.51 [M+3H]$^{3+}$, 728.64 [M+4H]$^{4+}$

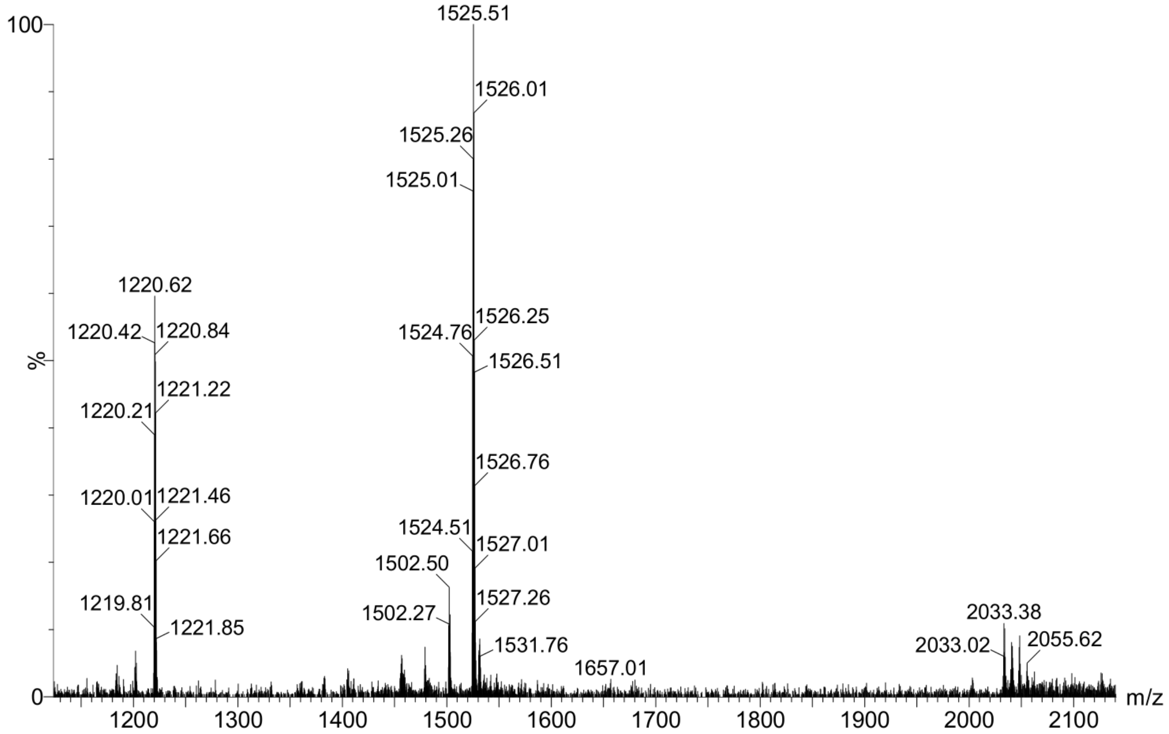

Figure S45. Mass spectrum of hexamer $\mathbf{1_12_7}$ from the LC-MS analysis of a stirred library made from peptides **1** and **2** (corresponding to Figure S6). Calculated m/z: 2032.56 $[M+3H]^{3+}$, 1524.67 $[M+4H]^{4+}$, 1219.94 $[M+5H]^{5+}$; observed m/z: 2032.65 $[M+3H]^{3+}$, 1524.51 $[M+4H]^{4+}$, 1219.81 $[M+5H]^{5+}$

6. Repeats of emergence experiments

A library of 6 mL ([**1**] = [**2**] = 0.5mM) was set up and allowed to oxidize at room temperature by the oxygen of the atmosphere until 70% of the monomers were oxidized (no agitation – ca. 12 days). This library was then split into 10 fractions (of 500 µL each). These 10 libraries were stirred at 45 °C and their compositions were monitored by UPLC. Three repeats of this experiment were performed: repeat A, B and C. The final composition of the libraries are shown below (after 7 days of stirring at 45 °C).

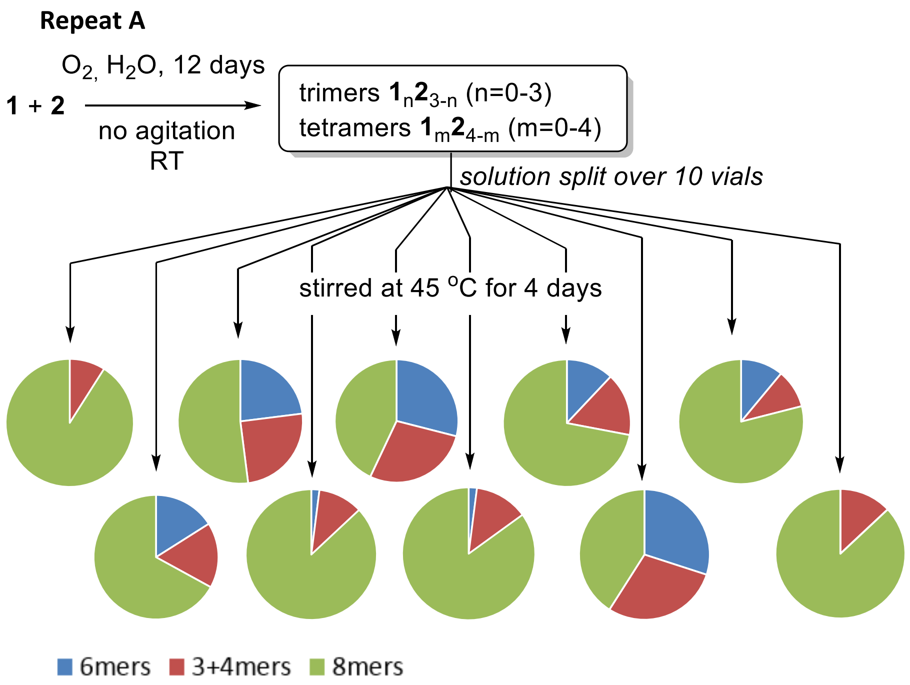

Figure S46. Final composition of the 10 libraries from repeat A: ([**1**] = [**2**] = 0.5 mM; oxidized at room temperature by the oxygen from the atmosphere until 70% of the monomers were oxidized, then stirred at 45 °C for 4 days).

Table S1. Final library composition of the 10 libraries corresponding to **Figure S46**, expressed as percentage of total observed peak area in the RP-UPLC data at 254 nm.

Entry	A	B	C	D	E	F	G	H	I	J
3+4mers	9	13	17	25	28	29	10	11	13	10
6mers	0	0	16	23	29	30	11	2	2	2
8mers	91	87	67	52	43	41	79	87	85	88

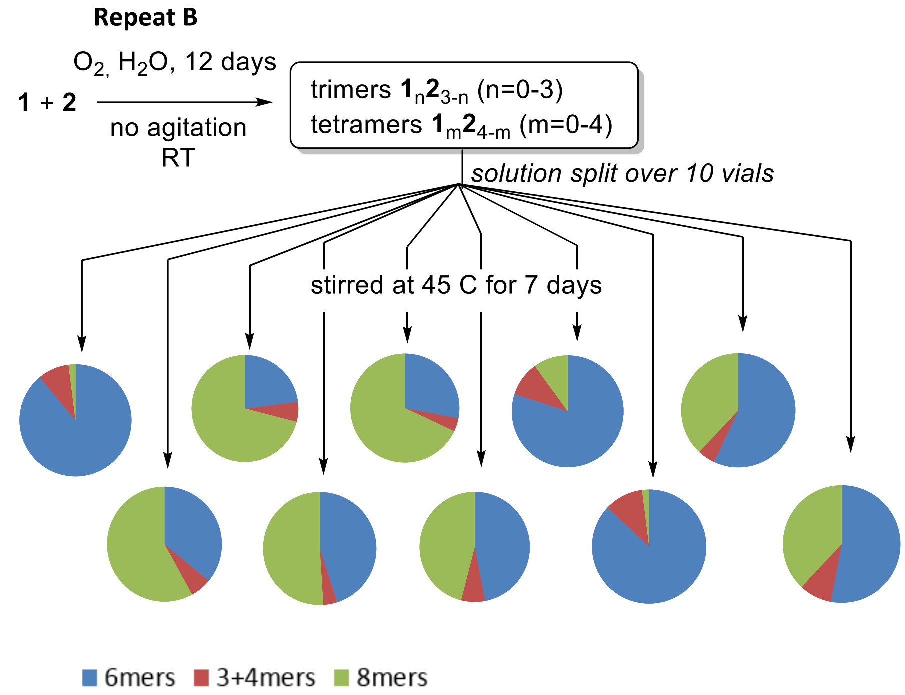

Figure S47. Final composition of the 10 libraries from repeat B: ([**1**] = [**2**] = 0.5 mM; oxidized at room temperature by the oxygen from the atmosphere until 70% of the monomers were oxidized, then stirred at 45°C for 7 days).

Table S2. Final library composition of the 10 libraries corresponding to **Figure S47**, expressed as percentage of total observed peak area in the RP-UPLC data at 254 nm.

Entry	A	B	C	D	E	F	G	H	I	J
3+4mers	6	6	9	4	4	7	10	11	5	9
6mers	23	36	89	45	28	47	80	87	57	53
8mers	71	58	2	51	68	46	10	2	38	38

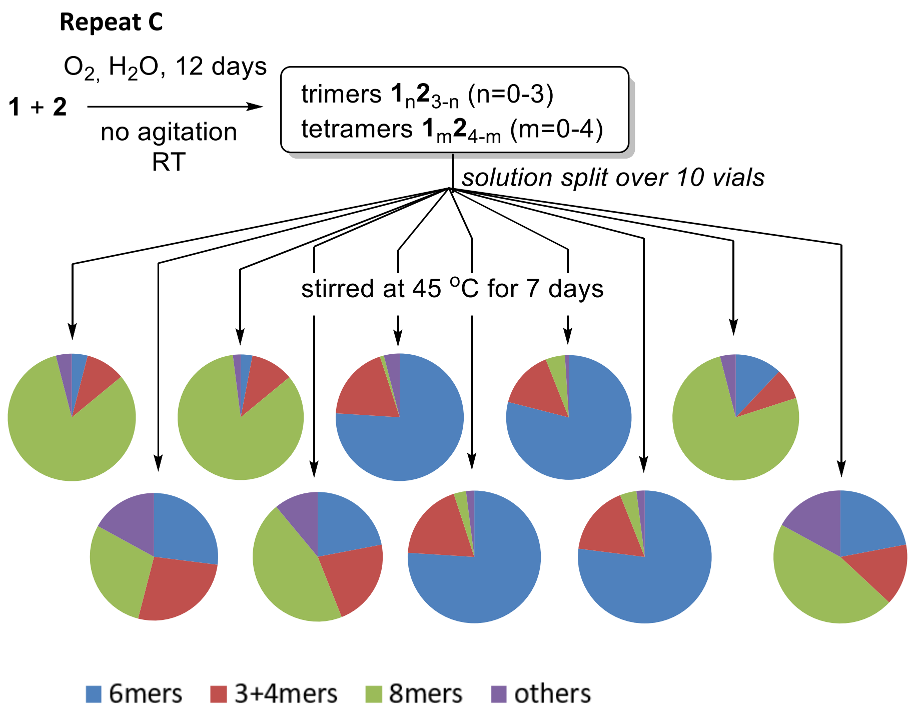

Figure S48. Final composition of the 10 libraries from repeat C: ([**1**] = [**2**] = 0.5 mM; oxidized at room temperature by the oxygen from the atmosphere until 70% of the monomers were oxidized, then stirred at 45°C for 7 days). Others indicate the peaks in the RP-UPLC chromatograms that could not be assigned with confidence using LC-MS.

Table S3. Final library composition of the 10 libraries corresponding to **Figure S48**, expressed as percentage of total observed peak area in the RP-UPLC data at 254 nm.

Entry	A	B	C	D	E	F	G	H	I	J
3+4mers	10	11	19	15	17	8	22	15	27	19
6mers	4	3	76	79	77	12	22	22	27	76
8mers	82	84	1	5	4	76	45	46	29	3
others	4	2	4	1	2	4	11	17	17	2

Table S4. Final library composition of the 10 libraries corresponding to **Figure 2**, expressed as percentage of total observed peak area in the RP-UPLC data at 254nm.

Entry	A	B	C	D	E	F	G	H	I	J
3+4mers	7	8	8	9	10	8	7	9	18	13
6mers	7	10	39	31	12	30	33	54	80	41
8mers	86	82	53	60	78	62	60	37	2	46

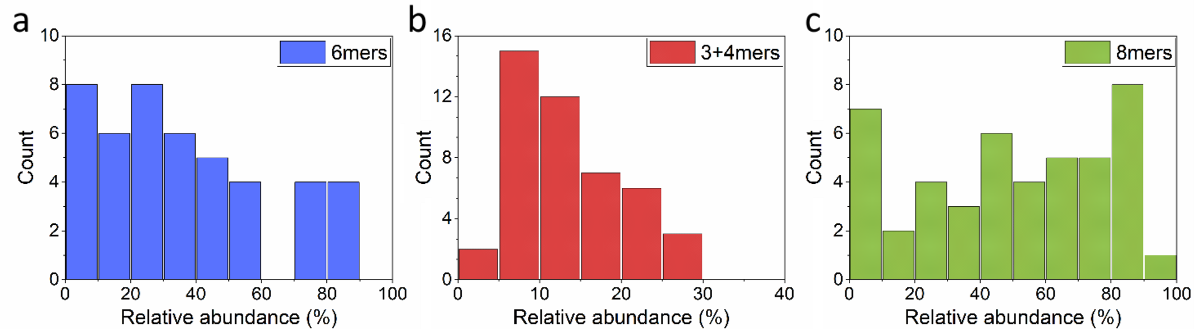

Figure S49. Histograms showing the spread in observed relative abundances of hexamers (a), trimers+tetramers (b) and octamers (c) for 45 DCLs (corresponding to **Tables S1-4** and **Table S7)** made from equimolar amounts of **1** and **2**.

7. Data for DCLs with varying building block ratios

Table S5. Final library composition of libraries (1 set of 5 libraries) containing 15 mol% **1**, expressed in percentage of total observed peak area in the RP-UPLC measurements at 254nm. "Others" indicate the peaks in the RP-UPLC chromatograms that could not be assigned with confidence using LC-MS. The average and corresponding standard deviations are also shown for each macrocycle size.

Entry	A	B	C	D	E	Average	St. Dev.
3+4mers	17.8	11.1	8.5	10.3	10.4	11.6	3.6
5mers	31.2	39.1	26.4	19.7	26.5	28.6	7.2
6mers	16.6	21.5	17.8	13.7	18.2	17.6	2.8
7mers	8.8	10.1	11.5	6.9	7.7	9.0	1.9
8mers	15.6	5.7	19.3	39.3	29.8	21.9	13.0
Others	10.0	12.6	16.4	10.1	7.5	11.3	3.4

Table S6. Final library composition of libraries (2 repeats of 5 libraries each) containing 33 mol% **1**, expressed in percentage of total observed peak area in the RP-UPLC measurements at 254nm. Fraction 8mer is determined by dividing the peak area for the 8mer by the total peak area of replicators (6mers+8mers). Others indicate the peaks in the RP-UPLC chromatograms that could not be assigned with confidence using LC-MS.

Entry	A	B	C	D	E	F	G	H	I	J
3+4mers	14.7	13.1	14.3	14.3	13.4	13.3	13.3	13.1	12.3	11.8
6mers	70.2	74.8	71.1	71.2	74.8	74.1	74.0	75.1	76.1	74.3
8mers	2.8	2.4	2.8	2.4	2.4	2.6	3.2	1.8	1.0	3.8
others	12.3	9.7	11.8	12.1	9.4	9.9	9.6	10.0	10.6	10.1
Fraction 8mer	0.04	0.03	0.04	0.03	0.03	0.03	0.04	0.02	0.01	0.05

Table S7. Final library composition of libraries (2 repeats of 5 libraries each) containing 45 mol% **1**, expressed in percentage of total observed peak area in the RP-UPLC measurements at 254nm. Fraction 8mer is determined by dividing the peak area for the 8mer by the total peak area of replicators (6mers+8mers). Others indicate the peaks in the RP-UPLC chromatograms that could not be assigned with confidence using LC-MS.

Entry	A	B	C	D	E	F	G	H	I	J
3+4mers	23.2	17.5	23.4	22.7	21.6	23.0	23.2	21.1	22.3	22.5
6mers	50.4	53.6	56.7	56.7	60.5	58.3	56.5	56.7	56.7	60.9
8mers	5.5	12.8	1.9	5.0	1.5	11.9	12.5	14.0	12.1	8.2
others	21.0	16.1	18.0	15.6	16.4	6.8	7.9	8.3	8.9	8.4
Fraction 8mer	0.10	0.19	0.03	0.08	0.02	0.17	0.18	0.20	0.18	0.12

Table S8. Final library composition of 5 libraries containing 50 mol% **1**, expressed in percentage of total observed peak area in the RP-UPLC measurements at 254nm. Fraction 8mer is determined by dividing the peak area for the 8mer by the total peak area of replicators (6mers+8mers). Others indicate the peaks in the RP-UPLC chromatograms that could not be assigned with confidence using LC-MS.

Entry	A	B	C	D	E
3+4mers	23.9	23.8	11.6	21.6	23.6
6mers	39.6	42.1	10.4	54.5	45.6
8mers	29.4	28.5	70.2	14.9	23.8
others	7.2	5.5	7.8	9.0	7.0
Fraction 8mer	0.43	0.40	0.87	0.21	0.34

Table S9. Final library composition of libraries (2 repeats of 5 libraries each) containing 55 mol% **1**, expressed in percentage of total observed peak area in the RP-UPLC measurements at 254nm. Fraction 8mer is determined by dividing the peak area for the 8mer by the total peak area of replicators (6mers+8mers). Others indicate the peaks in the RP-UPLC chromatograms that could not be assigned with confidence using LC-MS.

Entry	A	B	C	D	E	F	G	H	I	J
3+4mers	21.0	29.7	20.5	22.2	23.1	23.0	27.5	29.0	24.5	26.4
6mers	32.3	15.1	28.5	22.1	20.0	22.3	11.5	20.3	35.9	21.1
8mers	33.6	50.0	38.3	43.6	46.0	47.9	54.4	43.4	33.3	47.0
others	13.2	5.2	12.8	12.1	10.8	6.8	6.7	7.3	6.3	5.6
Fraction 8mer	0.51	0.77	0.57	0.66	0.70	0.68	0.83	0.68	0.48	0.69

Table S10. Final library composition of libraries (2 repeats of 5 libraries each) containing 67 mol% **1**, expressed in percentage of total observed peak area in the RP-UPLC measurements at 254nm. Fraction 8mer is determined by dividing the peak area for the 8mer by the total peak area of replicators (6mers+8mers). Others indicate the peaks in the RP-UPLC chromatograms that could not be assigned with confidence using LC-MS.

Entry	A	B	C	D	E	F	G	H	I	J
3+4mers	25.2	24.5	25.0	29.6	27.3	26.4	29.8	28.4	27.9	28.3
6mers	10.0	8.7	8.8	9.6	7.1	4.4	7.3	7.3	4.3	3.5
8mers	51.2	53.5	52.6	50.4	56.2	56.5	51.9	55.1	56.8	57.7
others	13.6	13.3	13.6	10.5	9.5	12.7	11.0	9.2	11.0	10.5
Fraction 8mer	0.84	0.86	0.86	0.84	0.89	0.93	0.88	0.88	0.93	0.94

Table S11. Final library composition of libraries (1 set of 5 libraries) containing 85 mol% **1**, expressed in percentage of total observed peak area in the RP-UPLC measurements at 254nm. The fraction 8mer is determined by dividing the peak area for the 8mer by the total peak area of replicators (6mers+8mers). "Others" indicate the peaks in the RP-UPLC chromatograms that could not be assigned with confidence using LC-MS.

Entry	A	B	C	D	E
3+4mers	15.4	13.4	11.4	14.3	15.6
6mers	0.5	1.5	1.3	1.3	0.8
8mers	71.7	67.0	72.9	69.6	70.6
Others	12.4	18.1	14.5	14.8	12.9
Fraction 8mer	0.99	0.98	0.98	0.98	0.99

Table S12. Average fraction of 8mer and 6mer and corresponding standard deviation for the final library compositions of libraries with varying ratios between **1** and **2**, expressed in percentage of total observed peak area in the RP-UPLC measurements at 254nm. All data in **Tables S1-S11** was used to calculate these values, resulting in 10 entries for each ratio, except for 50 mol% **1** which has 45 entries (including also the data in **Figure 2** and **Figures S46-S48**) and 85 mol% **1** which has 5 entries.

Mol% **1**	85	67	55	50	45	33
Average fraction 8mer	0.98	0.88	0.64	0.57	0.13	0.03
Average fraction 6mer	0.02	0.12	0.36	0.43	0.87	0.97
Standard deviation	0.01	0.04	0.10	0.32	0.06	0.01

8. Changing experimental conditions

Additional experiments were performed to study the influence of different parameters on the observed stochasticity in the nucleation of the different replicators (6mers and 8mers).

Effect of stirring the mother solution. The reason that in our experimental design the mother solution was not stirred and kept at room temperature prior to being split over separate vials, is that, in our experience, replicator emergence tends to be suppressed at low temperature and in the absence of stirring. Indeed, when the mother solution was immediately stirred at 45 °C for 7 days the observed final distribution is enriched in 8mers (see **Table S13** and **Figure S50**).

Table S13. Final composition of 10 libraries containing 50 mol% **1** where the mother solution was stirred at 45 °C, expressed in percentage of total observed peak area in the RP-UPLC measurements at 254nm. The fraction 8mer is determined by dividing the peak area for the 8mers by the total peak area of all replicators (6mers+8mers). "Others" indicate the peaks in the RP-UPLC chromatograms that could not be assigned with confidence using LC-MS.

Entry	A	B	C	D	E	F	G	H	I	J
3+4mers	3,8	3,3	2,0	4,6	4,0	3,1	3,5	4,3	4,2	4,2
6mers	27,6	34,7	19,1	38,3	37,4	17,9	13,4	19,0	12,9	28,4
8mers	58,7	52,2	69,7	51,1	48,0	69,2	75,6	67,9	75,1	61,4
Others	9,9	9,7	9,3	6,0	10,6	9,8	7,5	8,8	7,8	6,0
Fraction 8mer	0,68	0,60	0,78	0,57	0,56	0,79	0,85	0,78	0,85	0,68

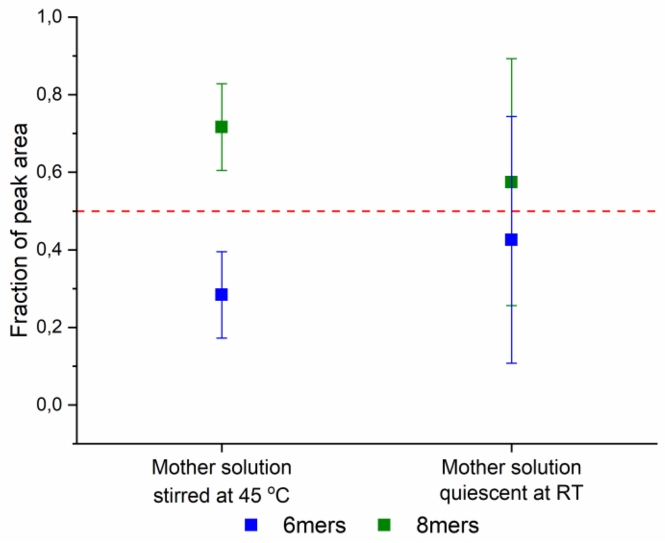

Figure S50. Variation in the fraction of the hexamer (blue) and octamer (green) replicators in the final library composition (determined by RP-UPLC) where the mother solution (50 mol% in **1**) was either stirred at 45 °C or kept at room temperature without agitation. The fraction is defined as the amount of observed replicator divided by the total amount of replicators (hexamers+octamers). The datapoints show the average and the error bars the standard deviation. The data point corresponding to the stirred mother solution represents the average over a total of 10 samples (**Table S13**). The data point for the quiescent mother solution was taken from the data shown in **Figure 5**). The dotted red line indicates the boundary between DCLs that are rich in hexamers (below the line) and rich in octamers (above the line).

Varying total building block concentration. We also investigated the influence of the total building block concentration on the stochastic nature of the emergence process. We therefore prepared a mother solution of 2.0 mM in building block with equimolar amounts of **1** and **2** (in 25 mM B_2O_3 buffer, pH 8.2). After incubation at room temperature without agitation until 80% of the material had been converted to disulfides the mother solution was used to prepare five DCLs each at five different concentrations: 2.0 mM, 1.0 mM, 500 μM, 100 μM and 50 μM. The observed relative abundances for each DCL after stirring for 7 days at 45 °C are shown in **Tables S14-16**. The variation in the observed fractions for the 8mer and 6mer replicators is shown in **Figure S51** and the total amount of observed replicators in **Figure S52**.

In all of these DCLs the 8mers were the dominant replicators. This could be a result of the 8mer already nucleating in the mother solution prior to its division over different vials. Nevertheless the degree in variability in replicator composition remained substantial, far exceeding that observed at most other building block ratios (see Figure 5). Note that at 50 μM and 100 μM concentrations the conversion of building block to replicator was less efficient than at higher building block concentrations (**Figure S52**). We believe this drop in total replicator formation can be explained by the reaction rates slowing down at lower concentrations. In addition, these concentrations start to approach the critical aggregation concentration of the mixture of monomers, trimers and tetramers (estimated previously to be in the 10-100 μM).[1]

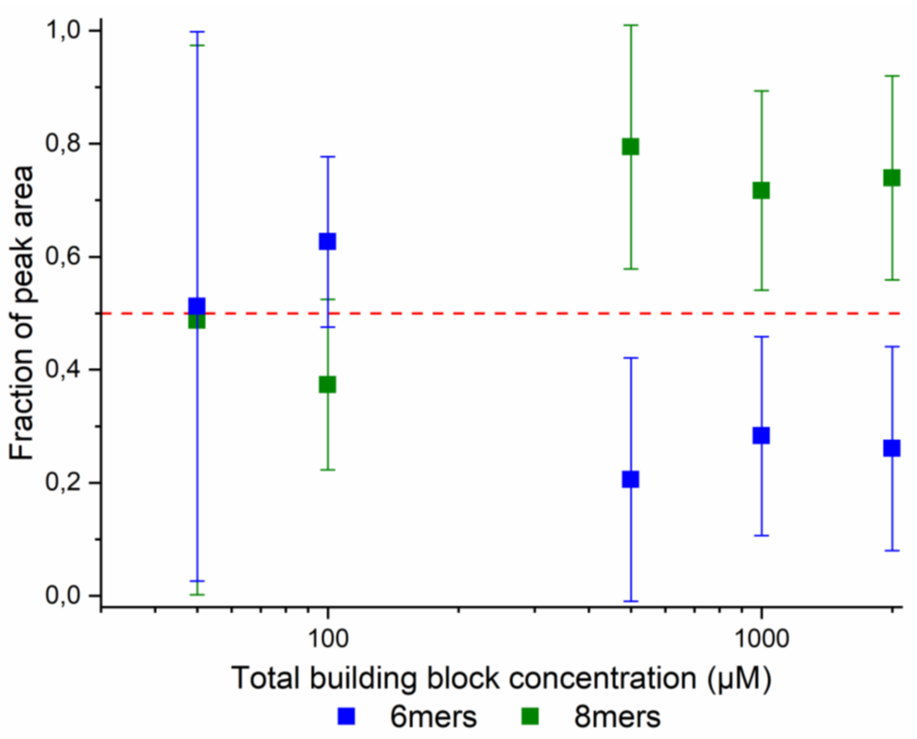

Figure S51. Variation in the fraction of the hexamer (blue) and octamer (green) replicators in the final library composition (determined by RP-UPLC) as a function of the total building block concentration. The fraction is defined as the amount of observed replicator divided by in the total amount of replicators (hexamers+octamers). The datapoints show the average and the error bars the standard deviation. The dotted red line indicates the boundary between DCLs that are rich in hexamers (below the line) and rich in octamers (above the line).

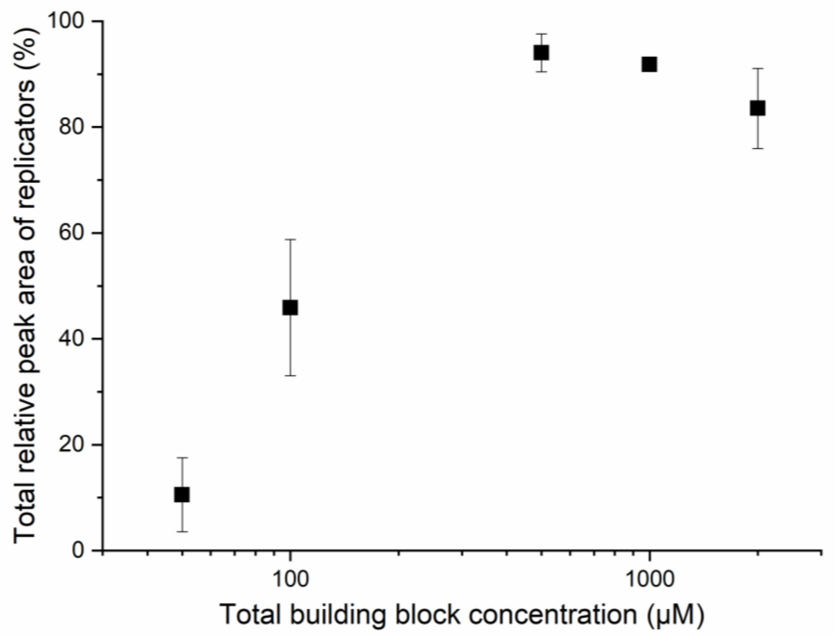

Figure S52. Variation in the conversion of building block into replicators (6mers + 8mers) in the final library composition (determined by RP-UPLC) as a function of the total building block concentration. The conversion is defined as the amount of observed replicator (6mers + 8mers) divided by the total peak area. The datapoints show the average and the error bars the standard deviation. Each data point is the averaged of a total of 5 samples (**Tables S14-18**).

Table S14. Final library composition of 5 libraries containing 50 mol% **1** (2.0 mM total concentration) , expressed in percentage of total observed peak area in the RP-UPLC measurements at 254nm. The fraction 8mer is determined by dividing the peak area for the 8mer by the total peak area of replicators (6mers+8mers). "Others" indicate the peaks in the RP-UPLC chromatograms that could not be assigned with confidence using LC-MS.

Entry	A	B	C	D	E
3+4mers	16,0	7,8	27,1	12,9	13,4
6mers	13,0	9,7	36,5	28,9	10,4
8mers	59,1	77,4	31,7	53,4	71,8
Others	11,9	5,2	4,7	4,8	4,5
Fraction 8mer	0,82	0,89	0,47	0,65	0,87

Table S15. Final library composition of 5 libraries containing 50 mol% **1** (1.0 mM total concentration) , expressed in percentage of total observed peak area in the RP-UPLC measurements at 254nm. The fraction 8mer is determined by dividing the peak area for the 8mer by the total peak area of replicators (6mers+8mers). "Others" indicate the peaks in the RP-UPLC chromatograms that could not be assigned with confidence using LC-MS.

Entry	A	B	C	D	E
3+4mers	8,5	6,8	7,9	9,7	6,2
6mers	0,0	32,5	34,8	19,3	36,5
8mers	87,9	49,0	54,0	68,5	54,0
Others	3,6	11,7	3,3	2,6	3,3
Fraction 8mer	1,00	0,60	0,61	0,78	0,60

Table S16. Final library composition of 5 libraries containing 50 mol% **1** (500 µM total concentration) , expressed in percentage of total observed peak area in the RP-UPLC measurements at 254nm. The fraction

8mer is determined by dividing the peak area for the 8mer by the total peak area of replicators (6mers+8mers). "Others" indicate the peaks in the RP-UPLC chromatograms that could not be assigned with confidence using LC-MS.

Entry	A	B	C	D	E
3+4mers	11,6	2,7	5,1	4,1	5,1
6mers	30,0	0,0	0,0	17,7	43,3
8mers	54,1	93,1	90,2	76,3	46,1
Others	4,2	4,2	4,6	2,0	5,5
Fraction 8mer	0,64	1,00	1,00	0,81	0,52

Table S17. Final library composition of 5 libraries containing 50 mol% **1** (100 µM total concentration) , expressed in percentage of total observed peak area in the RP-UPLC measurements at 254nm. The fraction 8mer is determined by dividing the peak area for the 8mer by the total peak area of replicators (6mers+8mers). "Others" indicate the peaks in the RP-UPLC chromatograms that could not be assigned with confidence using LC-MS.

Entry	A	B	C	D	E
3+4mers	33,3	56,4	61,4	34,8	51,9
6mers	17,9	26,0	22,5	37,8	19,1
8mers	21,7	10,7	4,6	22,2	18,2
Others	27,2	6,9	11,5	5,3	10,9
Fraction 8mer	0,55	0,29	0,17	0,37	0,49

Table S18. Final library composition of 5 libraries containing 50 mol% **1** (50 µM total concentration) , expressed in percentage of total observed peak area in the RP-UPLC measurements at 254nm. The fraction 8mer is determined by dividing the peak area for the 8mer by the total peak area of replicators (6mers+8mers). "Others" indicate the peaks in the RP-UPLC chromatograms that could not be assigned with confidence using LC-MS.

Entry	A	B	C	D	E
3+4mers	89,8	74,8	94,7	94,5	84,8
6mers	5,9	17,8	0,0	0,0	9,8
8mers	0,0	1,6	5,3	5,5	5,4
Others	4,3	5,9	0,0	0,0	0,0
Fraction 8mer	0,00	0,08	1,00	1,00	0,36

9. Thioflavin T (ThT) fluorescence assay

A ThT stock solution (2.2 mM) was prepared in 10 mL phosphate buffer (50 mM phosphate, pH 8.2) and filtered through a 0.2 µm syringe filter. On the day of analysis, 50µL of the stock solution was diluted into 5 mL phosphate buffer (50 mM phosphate, pH 8.2) to generate the working solution of 22 µM. The fluorescence intensity of 450 µL ThT solution was measured by excitation at 440 nm (slit width 5 nm) and emission between 480-700 nm (slit width 5 nm), averaging 3 accumulations. An aliquot of 80µL of peptide solution (100µL in borate buffer) was added to the HELMA 10*2 mm quartz cuvette, incubated for 2 min, and the intensity was measured over 3 accumulations. All fluorescence measurements were performed on a JASCO FP6200 fluorimeter equipped with a 480 nm high pass cut-off filter on the emission channel to avoid high order diffractions coming from the excitation.

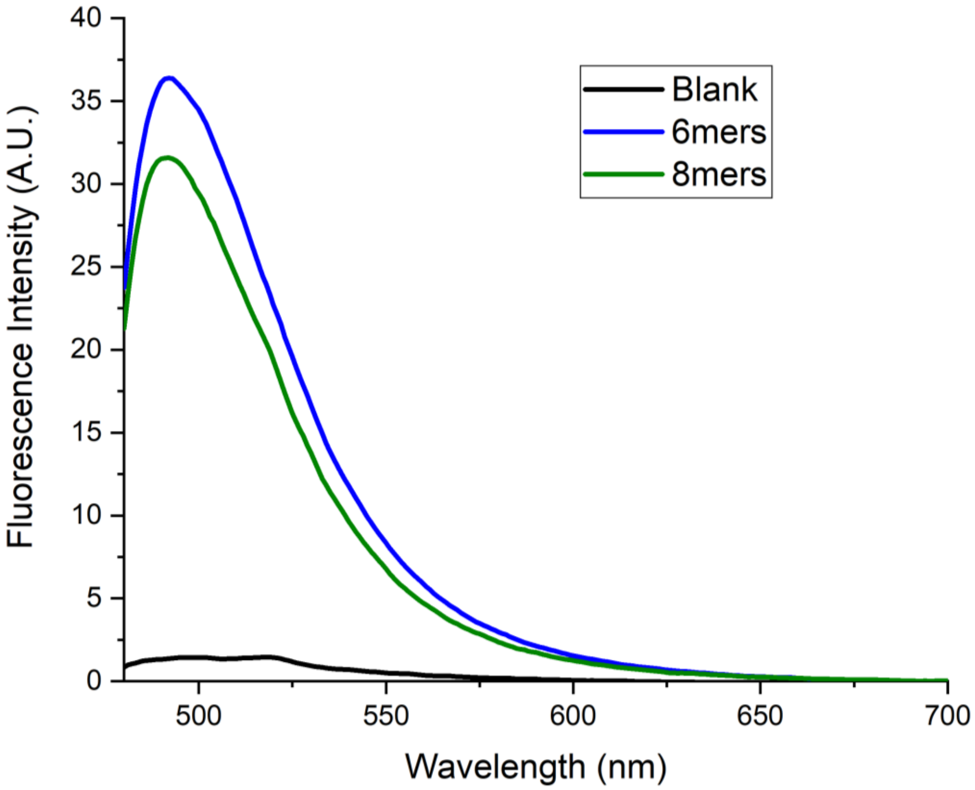

Figure S53. Thioflavin T fluorescence emission of DCLs made from building block **1** and **2** (50 mol% each). Blue: library containing mostly 6mers. Green: library containing mostly 8mers.

10. Circular dichroism (CD) spectroscopy

All CD spectra were recorded using a JASCO J715 spectrophotometer and HELMA quartz cuvettes with a path length of 1 mm. Spectra were recorded at room temperature from 190 nm to 350 nm, with a 1nm step interval and averaged over 3 scans using a scanning speed of 200nm/min. Solvent spectra were subtracted from all reported spectra. Samples were diluted using borate buffer (50 mM, pH 8.2) to a concentration of 0.15 mM. The concentration is expressed in monomer units.

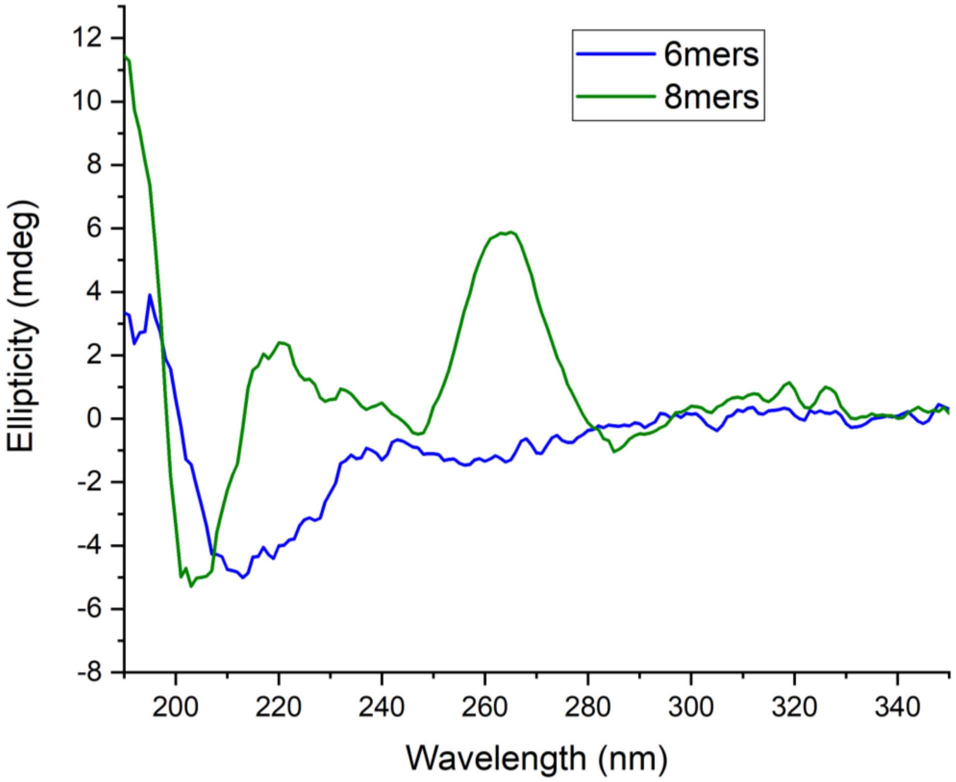

Figure S54. CD spectra of DCLs made from building block **1** and **2** (50 mol% each). Blue: library containing mostly 6mers. Green: library containing mostly 8mers.

11. Negative Staining Transmission Electron Microscopy

Sample Preparation

An aliquot (5.0 μL) of the sample was deposited on a 400 mesh copper grid covered with a thin carbon film (Van Loenen Instruments). After 60 seconds, the droplet was blotted on filter paper. The sample was then stained twice (5.0 μL each time) with a solution of 2% uranyl acetate deposited on the grid and blotted on filter paper after 30 seconds each time. The grids were observed in a Philips CM120 cryo-electron microscope operating at 120 kV. Images were recorded on a slow scan CCD camera.

Analysis

The libraries that consist of mostly octamers (**Figure S55**, left panels) shows well defined fibers that create large supramolecular networks. The fibers are laterally associated but show no helical or twisting structures. For the libraries that contain mostly hexamers (**Figure S55**, right panels), the observed fibers are not easily distinguishable. They are laterally associated and have a twisted morphology.

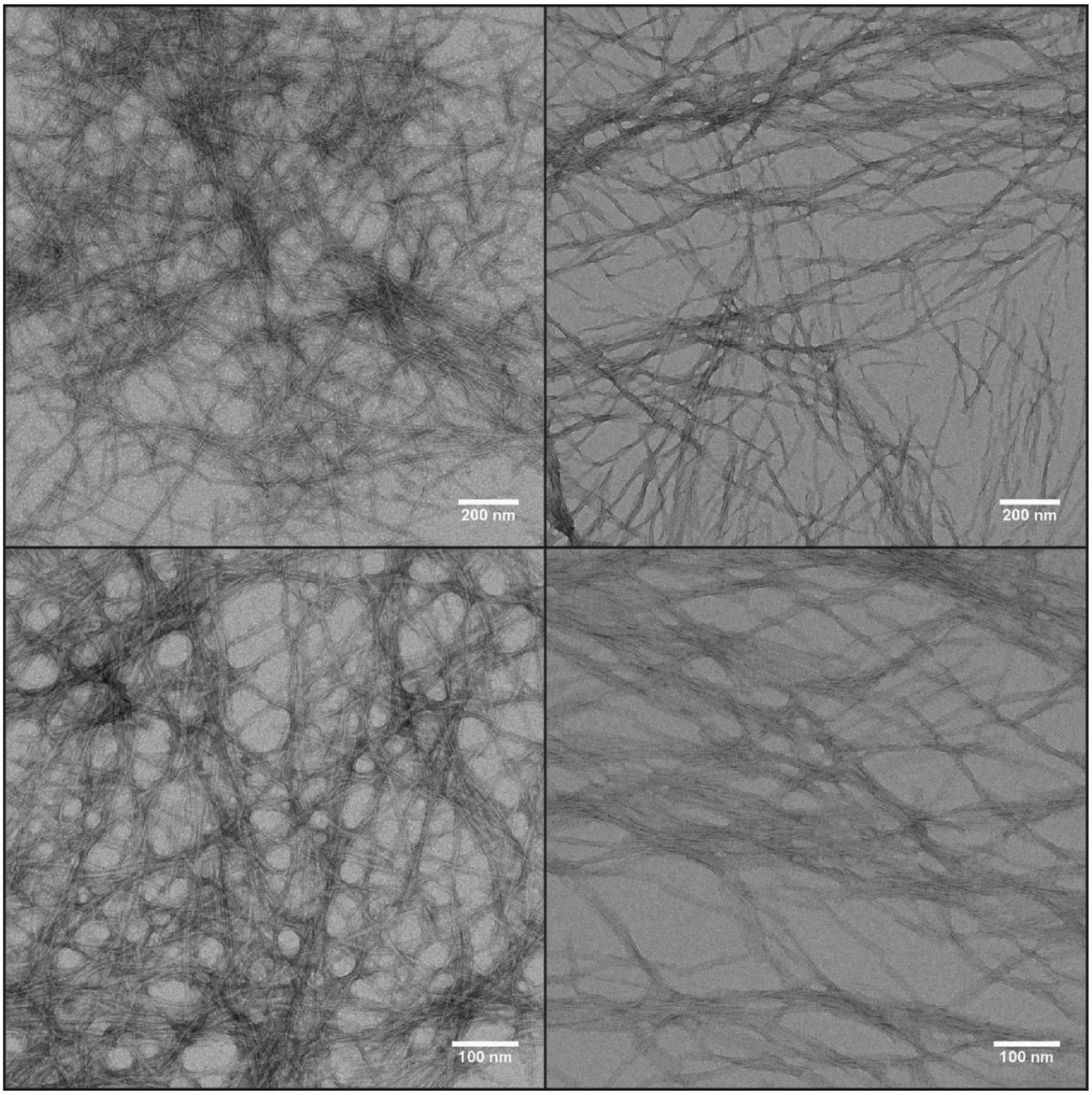

Figure S55. TEM micrographs of libraries made from building block **1** and **2**. Left side: Samples consisting mostly of octamers, with a network-like structure created from well-defined fibers. Samples consisting of mostly hexamers that show a twisted structure of laterally associated fibers.

12. Correlation between UPLC and ThT data

RP-UPLC is the only analytical technique that can distinguish between self-replicators with different macrocycle sizes. The analysis is however indirect, the fibers fall apart in individual macrocycles during analysis. To justify the use of UPLC data as a measure for (fibrous) replicator formation we followed five separate DCLs containing equimolar amounts of **1** and **2** with both ThT and UPLC. Figure S56 shows that the obtained maximum ThT fluorescence intensities (at 490 nm) correlate well with the formation of 6mer and 8mer macrocycles followed by UPLC, which justifies the use of the UPLC data of 6mer and 8mer formation as a measure of replicator formation.

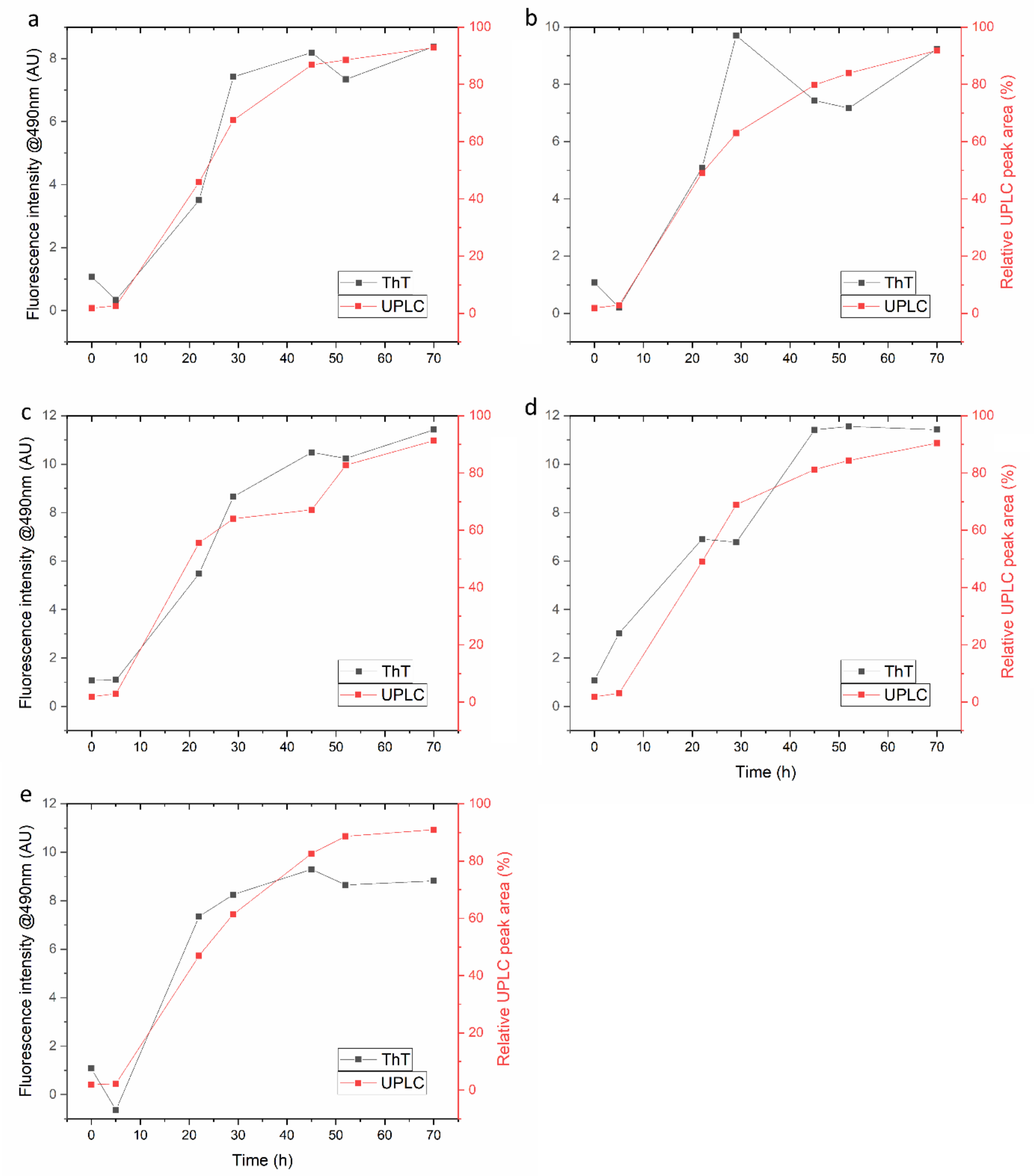

Figure S56. Five repeats (a-e) of replicator emergence in DCLs made from building block **1** and **2**. Total replicator formation (6mer and 8mer), expressed as relative UPLC peak area, is shown on the left axes (black) and ThT fluorescence intensity at 490 nm on the right axes (red).

13. Data Fitting

In our model we have included this nucleation process as free fitting parameters for each hexamer and octamer separately. This means that each replicator nucleation event is fitted with a different nucleation time. The distributions of the found nucleation times by fitting this model to the UPLC traces provides strong indirect evidence that the nucleation events are indeed the cause of the observed stochastic behaviour.

The relative concentration of the different species (hexamer, octamer and trimers/tetramers) was fit to a system of ordinary differential equations (ODEs, **Scheme S1**, see also main text) using Symfit.[1] The fit was performed 1000 times using random initial values for the parameters. The best resulting fit had a regression coefficient of 0.933 and is depicted in **Figure S57**. The corresponding fit parameters are depicted in Figure 4 in the main text, and can be found in **Table S19**. In order to assess the uncertainty in the fit parameters we numerically determined the Hessian matrix using a second order central difference scheme where the matrix was constructed to be symmetric. The covariance matrix was obtained by inverting the Hessian, and the standard deviations of the fit parameters were taken as the square root of the diagonal elements of the covariance matrix.

Table S19. Best fit parameters and associated standard deviations (StdDev). The numbering corresponds to the experiments as follows: 0-8 correspond to the experiment shown in **Figure 2** and **Table S4**; 9-18 correspond to Repeat C (**Figures S48** and **Table S3**); 19-28 correspond to Repeat A (**Figures S46** and **Table S1**).

	Value	StdDev
$t_{0, hex, 0}$	0.10	1.35e-2
$t_{0, oct, 0}$	-0.28	1.35e-2
$t_{0, hex, 1}$	0.26	1.25e-2
$t_{0, oct, 1}$	-0.08	1.25e-2
$t_{0, hex, 2}$	0.40	1.20e-2
$t_{0, oct, 2}$	0.46	1.20e-2
$t_{0, hex, 3}$	-0.07	1.03e-2
$t_{0, oct, 3}$	-0.08	1.03e-2
$t_{0, hex, 4}$	-0.10	1.12e-2
$t_{0, oct, 4}$	-0.31	1.12e-2
$t_{0, hex, 5}$	-0.31	1.16e-2
$t_{0, oct, 5}$	-0.30	1.16e-2
$t_{0, hex, 6}$	-0.58	1.20e-2
$t_{0, oct, 6}$	-0.04	1.20e-2
$t_{0, hex, 7}$	-0.58	7.04e-2
$t_{0, oct, 7}$	-0.04	7.04e-2
$t_{0, hex, 8}$	0.58	4.58e-2
$t_{0, oct, 8}$	0.23	4.58e-2
$t_{0, hex, 9}$	1.15	2.94e-2
$t_{0, oct, 9}$	0.77	2.94e-2
$t_{0, hex, 10}$	-0.68	4.07e-3
$t_{0, oct, 10}$	-0.16	4.09e-3
$t_{0, hex, 11}$	-0.43	1.15e-2
$t_{0, oct, 11}$	0.09	1.15e-2
$t_{0, hex, 12}$	-0.28	1.15e-2
$t_{0, oct, 12}$	0.22	1.16e-2
$t_{0, hex, 13}$	0.67	1.22e-2
$t_{0, oct, 13}$	0.45	1.23e-2
k_{hex}	0.50e-4	1.49e-6

	Value	StdDev
$t_{0, hex, 14}$	1.04	1.45e-2
$t_{0, oct, 14}$	1.11	1.45e-2
$t_{0, hex, 15}$	1.16	1.45e-2
$t_{0, oct, 15}$	1.24	1.45e-2
$t_{0, hex, 16}$	0.90	1.71e-2
$t_{0, oct, 16}$	1.06	1.71e-2
$t_{0, hex, 17}$	-0.58	1.38e-2
$t_{0, oct, 17}$	-0.11	1.38e-2
$t_{0, hex, 18}$	0.19	2.86e-2
$t_{0, oct, 18}$	-0.59	2.87e-2
$t_{0, hex, 19}$	0.05	4.54e-3
$t_{0, oct, 19}$	-0.61	4.56e-3
$t_{0, hex, 20}$	-0.17	2.65e-4
$t_{0, oct, 20}$	-0.29	-1.71e-4
$t_{0, hex, 21}$	-0.06	1.59e-2
$t_{0, oct, 21}$	-0.05	1.59e-2
$t_{0, hex, 22}$	-0.14	3.95e-2
$t_{0, oct, 22}$	-0.01	3.95e-2
$t_{0, hex, 23}$	-0.24	1.28e-2
$t_{0, oct, 23}$	-0.12	1.28e-2
$t_{0, hex, 24}$	-0.13	4.12e-5
$t_{0, oct, 24}$	-0.46	4.57e-5
$t_{0, hex, 25}$	0.15	2.89e-2
$t_{0, oct, 26}$	-0.60	2.89e-2
$t_{0, hex, 26}$	-0.02	2.24e-2
$t_{0, oct, 27}$	-0.70	2.24e-2
$t_{0, hex, 28}$	0.11	1.37e-2
$t_{0, oct, 28}$	-0.50	1.37e-2
k_{oct}	5.5e-4	2.00e-6

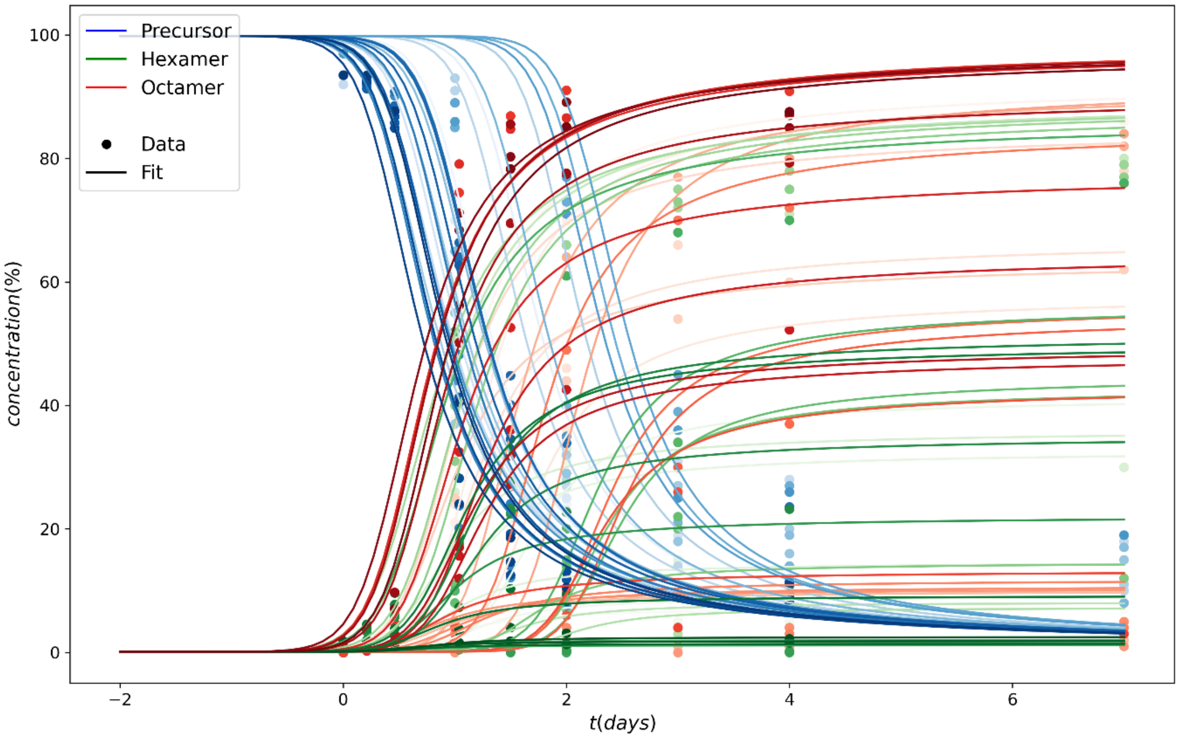

Figure S57. Fit of the ODE model shown in **Scheme S1** to RP-UPLC data. The fits are depicted as solid lines, the RP-UPLC data as points. Precursors are blue, hexamers are green, octamers are red. X axis depicts time (in days), Y axis relative concentration (in %). t=0 was defined to be the time of the first RP-UPLC measurement. The regression coefficient (R^2) is 0.933.

Scheme S1. Simplified model system of ODEs to which the RP-UPLC data is fitted to obtain an estimate of the nucleation times for the self-replicators. The fit was performed using Symfit.[1]

$$f(t, t_0) = \frac{1}{(1 + \exp(-10 * (t - t0)))}$$

$$\frac{\partial C_{hex}}{\partial t} = f(t, t_{0,hex}) * k_{hex} * C_{hex} * C_{precursor}^2$$

$$\frac{\partial C_{oct}}{\partial t} = f(t, t_{0,oct}) * k_{oct} * C_{oct} * C_{precursor}^2$$

$$\frac{\partial C_{precursor}}{\partial t} = \frac{-\partial C_{hex}}{\partial t} - \frac{\partial C_{oct}}{\partial t}$$

$$with$$

$$t_{0,hex} = t_{0,avg} - \Delta t_0/2$$

$$t_{0,oct} = t_{0,avg} + \Delta t_0/2$$

$$C_{hex}(t = -2) = 0.1$$

$$C_{oct}(t = -2) = 0.1$$

$$C_{precursor}(t = -2) = 99.8$$

$$0 \leq k_{hex} \leq 1.10e^{-3}$$

$$0 \leq k_{oct} \leq 1.10e^{-3}$$

$$7 \leq \Delta t_0 \leq 7$$

$$-2 \leq t_{0,avg} \leq 7$$

14. References

(1) Maity, S.; Ottele, J.; Santiago, G. M.; Frederix, P.; Kroon, P.; Markovitch, O.; Stuart, M. C. A.; Marrink, S. J.; Otto, S.; Roos, W. H., Caught in the Act: Mechanistic Insight into Supramolecular Polymerization-Driven Self-Replication from Real-Time Visualization. *J. Am. Chem. Soc.* **2020,** *142*, 13709-13717.

(2) Roelfs, M.; Kroon, P. C. SymFit. *Zenodo*. **2019**. https://doi.org/10.5281/zenodo.1133336
